# Red electroluminescent Cu_4_I_4_ bipyramids with external quantum efficiency beyond 40%

**DOI:** 10.1038/s41467-026-75381-2

**Published:** 2026-07-07

**Authors:** Jie Ren, Xiudan Song, Chunlei Zhong, Jianan Sun, Xinjie Wang, Chunbo Duan, Chunmiao Han, Ying Wei, Hui Xu

**Affiliations:** 1 Department of Applied Chemistry, School of Chemistry and Material Science, Heilongjiang University, Harbin, China; 2 School of Physical Science and Technology, Heilongjiang University, Harbin, China; 3 Department of Electrical Engineering, School of Mechanical and Electrical Engineering, Heilongjiang University, Harbin, China

**Keywords:** Organic LEDs, Organic LEDs, Optical materials

## Abstract

The development of red cluster light-emitting devices (CLED) lags far behind blue and green congeners with respect to efficiencies, since multiple excited states of red cluster molecules are involved in non-radiation during electroluminescence. Herein, we accurately optimize excited states of a red bipyramidal [PXZDPPQ]_2_Cu_4_I_4_, whose ligand integrates conjugation-extended 2-diphenylphosphineylquinoline (DPPQ) and strong electron-donating phenoxazine (PXZ), giving rise to desired excited state locations of the outer intraligand charge transfer (^n^LCT)-featured first singlet (S_1_) and triplet (T_1_) excited states and the inner high-lying metal-ligand charge transfer (^n^MLCT) states. Its outer and highly radiative ^n^LCT states are spatially and energetically advantageous in carrier capture and exciton confinement; meanwhile, its inner and high-lying ^n^MLCT states are protected from collisional quenching, and support cross transitions between ^n^LCT states to realize exactly balanced dual emissions with a ratio of 51/49. [PXZDPPQ]_2_Cu_4_I_4_ achieves eightfold increased photoluminescence quantum yield of 93.6%, tenfold increased external quantum efficiency reaching 43.7% as a new record for all kinds of planar red light-emitting devices, and 20% improved exciton utilization efficiency in comparison to the congener with the reverse excited state location. These results demonstrate the unique merit of cluster materials in exciton engineering and their potential for next-generation full-color displays.

## Abstract (web summary)

Red cluster light-emitting devices suffer low efficiency due to multiple excited states causing nonradiative loss. Ren et al. design a copper iodide cluster with tailored excited states to balance dual emission and enhance radiation, achieving 100% exciton utilization and record efficiency.

## Introduction

**Fig. 1:**
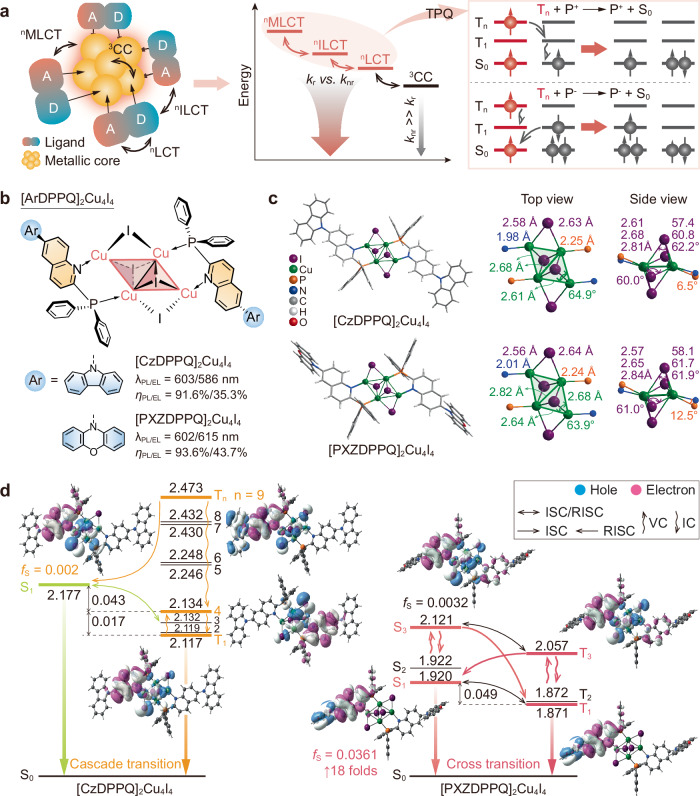
Structures and simulated excited-state characteristics of [ArDPPQ]_2_Cu_4_I_4_. **a** Multi-component charge transfer states in ligands stabilized metallic clusters and their involvement in radiative and nonradiative processes, in which high-lying triplet states accelerate triplet-polaron quenching (TPQ) through coupled charge exchange and internal conversion. “D” and “A” refer to donor and acceptor groups in ligands. MLCT, LCT, ILCT and CC refer to metal-ligand, intra-ligand, inter-ligand and intra-core charge transfer, whose superscripts “n” are l and 3 corresponding to singlet and triplet, respectively. *k* is rate constant, whose subscripts of “r” and “nr” refer to radiative and nonradiative, respectively. “S” and “T” refer to singlet and triplet states, whose subscripts of “0”, “1” and “n” refer to ground, the first excited and high-lying states, respectively. “P” refers to polaron, whose superscripts of “+” and “-” refer to positive and negative, respectively. **b** Chemical structures of [ArDPPQ]_2_Cu_4_I_4_. DPPQ is 2-(diphenylphosphaneyl)quinoline. “Ar” refers to donor groups of carbazole (Cz) and phenoxazine (PXZ) at 6-position of quinoliine. **c** Single crystal structures of [ArDPPQ]_2_Cu_4_I_4_. N,P-coordinated Cu_4_I_4_ skeletons and the related N/P-Cu and Cu-Cu bond lengths in Cu_4_ rhomboids and degrees of bond angles are highlighted. *S*_Cu4_ is the area of Cu_4_ rhomboids. Chemical and single-crystal structures of [DPPQ]_2_Cu_4_I_4_ featuring linear metallic core are presented in supplementary information for comparison. **d** Frontier orbital contours and key parameters of the singlet and triplet excitations for the lowest ^n^ILCT and ^n^MLCT states simulated with natural transition orbital (NTO) analysis. The contours of “hole” and “electron” are distinguished with blue and pink colors, respectively. Values of excited-state energy levels are listed*. f*_S_ is singlet oscillator strength. ISC, RISC, VC and IC refer to intersystem crossing, reverse ISC, vibrational coupling and internal conversion, respectively.

Recently emerged cluster light-emitting diodes (CLED) hold promise as next-generation display and lighting technologies, owing to wide color gamut, high electroluminescent performance and easy manufacturing^1,2^. The employed cluster emitters have the structures of organic ligands stabilized metallic cores (Fig. 1a), and therefore integrate merits of inorganic^3,4^ and organic semiconductors^5,6^ in thermo- and photo- stability^7^, conductivity^8,9^ and optical adjustability^10,11^. Up to present, CLEDs have already realized the external quantum efficiencies (EQE, *η*_EQE_) up to 30% and luminance beyond 10^5^ nits, which were at top performance level among all kinds of electroluminescent technologies^12^. Nonetheless, compared to sky-blue and green counterparts, the development of red CLEDs lags far behind in terms of color purity and efficiencies (Suppl. Fig. 1). This status quo substantially limits the practical display applications.

Despite discrete energy levels similar to mononuclear complexes^13–16^, the hybrid and multinuclear structures of clusters induce multiple intramolecular interactions, and thus generate multi-component excited states, in which ligand-centered (LC) excited states containing metal/counterion-ligand (^n^M/XLCT, *n* = 1 and 3 for singlet and triplet) and intra- (^n^LCT) and inter-ligand charge transfer (^n^ILCT) states are markedly more radiative than cluster-center (^3^CC) triplet states based on *d*-*d* transitions (Fig. 1a)^17^. Besides the competition between LC and CC states in exciton population, the complicated transitions between LC states increase energy loss and non-radiative probabilities, which are more serious in the case of red clusters, according to energy gap rule. Due to synergistically exothermic electron transfer and internal conversion, high-lying LC states of red clusters more easily capture charges and electro-generated excitons to increase the probability of charge-exciton interactions and worsen triplet-polaron quenching (TPQ), which is commonly in direct proportion to current and exciton densities^18^.

It is demonstrated that increasing nuclear number^19,20^ of noble-metal clusters and introducing hetero-metals^21^ can effectively enhance metal-metal interactions for reducing energy levels of ^3^CC and MLCT states and consequently shifting emission to red region. However, ^3^CC predominance limited their photoluminescence quantum yields (PLQY, *η*_PL_) and *η*_EQE_ within 70% and 15%, respectively^21^. To avert this issue, ligands were modified with carrier-transporting groups and/or donor-acceptor (D-A) structures to increase ILCT and LCT components, accompanied by decreasing the nuclear number of hetero-metallic cores to restrain ^3^CC state. By this way, *η*_PL_ of noble-metal clusters can be improved to over 90%, but their *η*_EQE_ values can hardly exceed 20%, corresponding to exciton utilization efficiencies (EUE, *η*_EUE_) less than 70%, due to TPQ during the electroluminescence process^22,23^.

Different to other d^10^ metals, copper in hetero-metallic clusters can finely balance metal-metal interactions and spin-orbital coupling (SOC) and thereby modulate ^3^CC states^24,25^. As a result, copper hybridized silver clusters can simultaneously realize high *η*_PL_ and *η*_EQE_ up to > 90% and 26%, respectively^26^. Obviously, homo-copper clusters are more advantageous in sustainability and environmental friendliness, but their emissions can hardly reach the red region, due to the relatively weak Cu-Cu interactions^27,28^. A feasible solution is introducing counterions to enhance intra-cluster charge transfer, which was verified by an AuCu_4_I_4_ cluster with orange electroluminescence peak wavelength (*λ*_EL_) at 586 nm and *η*_PL_ and *η*_EQE_ reaching 93% and 20.8%^29^.

Actually, moderate SOC endows copper iodide emitters^30–32^ with both thermally activated delayed fluorescence (TADF) and phosphorescence (PH) from their first singlet (S_1_) and triplet (T_1_) states^33–36^. This establishes the basis for constructing dual emissive materials with suitable TADF/PH ratios^37–42^. Dual emission with doubled radiation channels can undoubtedly alleviate exciton accumulation-induced quenching, corresponding to an “ideal” TADF/PH ratio of 50/50. Besides increasing structural rigidity to alleviate John-Teller distortion-induced excited-state relaxation^43–45^, metallic core configurations of Cu_4_I_4_ clusters can be adjusted as cube^46^, chair^47^, or rhomboid^48^ to more delicately modulate excited-state compositions for MLCT enhancement and TADF/PH optimization^49–51^. Furthermore, our previous results verified that ligand engineering provides a powerful toolbox for significantly improving radiative efficiencies, which can not only modify coordination environment^52^ and further strengthen ligand-metallic core interactions^53,54^, but also increase ILCT and LCT components in the S_1_ and T_1_ states^55^, leading to *η*_PL_ and *η*_EQE_ as high as ~100% and ~35%^56^.

Nonetheless, up to now, the reported copper iodide CLEDs can only reach yellow region for the maximum. High-efficiency red electroluminescent Cu_n_I_m_ clusters are still absent, since further enhancing intra-cluster charge transfer to narrow bandgaps would markedly increase polarities of excited states, and consequently worsen exciton quenching, especially TPQ on the high-lying states, inducing sharply decreased *η*_EQE_. In order to simultaneously achieve *λ*_EL_ > 600 nm and *η*_EQE_ > 30%, the clusters should have: (i) D-A ligands with extended conjugation: beside appropriate electron donating and withdrawing effects to support effective charge transfer, conjugation extension of donor and/or acceptor groups can further reduce energy levels of both ILCT and LCT states without increasing excited state polarities; (ii) high-lying excited states embedded in skeletons: besides modulating the excited-state energy levels, the excited-state distributions of the lowest outside and the high-lying inside can increase the probabilities of direct carrier recombination on the S_1_ and T_1_ states, and prevent the high-lying excited states from interactions with excitons and polarons for quenching suppression.

In this contribution, we report the high-efficiency red CLEDs based on two Cu_4_I_4_ bipyramids named [CzDPPQ]_2_Cu_4_I_4_ and [PXZDPPQ]_2_Cu_4_I_4_, whose ternary N,P-hybrid ligands are based on 2-diphenylphosphineylquinoline (DPPQ) respectively substituted with carbazole (Cz) and phenoxazine (PXZ) donor groups (Fig. 1b). We replace conventional pyridine with quinoline to construct conjugation-extended D-A ligands, making *λ*_EL_ of [PXZDPPQ]_2_Cu_4_I_4_ significantly increased by 100 nm to ~620 nm. Simultaneously, the predominant components of the S_1_ and T_1_ states change from ^3^CC of [DPPQ]_2_Cu_4_I_4_, ^n^MLCT of [CzDPPQ]_2_Cu_4_I_4_ to ^n^LCT of [PXZDPPQ]_2_Cu_4_I_4_, in accord with the intensities of electron-donating effect for Cz and PXZ groups. As a result, *η*_PL_ values of both [CzDPPQ]_2_Cu_4_I_4_ and [PXZDPPQ]_2_Cu_4_I_4_ beyond 90% are eight folds of that of [DPPQ]_2_Cu_4_I_4_. On the contrary to the case of [CzDPPQ]_2_Cu_4_I_4_, the MLCT-featured high-lying S_3_ and T_3_ states of [PXZDPPQ]_2_Cu_4_I_4_ are localized on its coordination skeleton and encapsulated by its LCT-featured S_1_ and T_1_ states. This excited-state distribution of [PXZDPPQ]_2_Cu_4_I_4_ accelerates its singlet radiation with eighteen-fold increased oscillator strength (*f*_S_), most balances dual emission with TADF/PH ratio of 51/49 through crossing transitions between the S_n_ and T_n_ states (*n* = 1–3) via intersystem crossing (ISC) and reverse ISC (RISC), and markedly alleviates TPQ for its devices, in contrast to the distinct TPQ of [CzDPPQ]_2_Cu_4_I_4_ based analogs. Consequently, [PXZDPPQ]_2_Cu_4_I_4_ realizes red electroluminescence with the record-high *η*_EQE_ reaching 43.7% among all kinds of planar light-emitting devices and reduced roll-offs. These results manifest the feasibility of ligand engineering in accurate excited-state optimization and consequent advantages of cluster materials for optoelectronic applications.

## Results

### Design and construction of red electroluminescent copper iodide clusters

We chose Cu_4_I_4_ clusters stabilized with N,P-hybrid ligands, e.g., [DPPy]_2_Cu_4_I_4_ (DPPy = diphenylphosphineylpyridine), since their short Cu-Cu distances of ~2.8 Å are beneficial to strengthen Cu-Cu interactions for emission bathochromic shift^54^. As a proof of concept, we used quinoline instead of pyridine in DPPy to construct the coordination unit DPPQ with larger conjugation (Suppl. Figs. 2–8). Despite planar configuration of quinoline, its fused benzene ring still generates steric hindrance at coordination direction to moderately distort the geometries of metallic cores for structural rigidity enhancement and non-radiation reduction. It is believed that the predominance of ^3^CC, ^n^MLCT and ^n^LCT states in the S_1_ and T_1_ states is in direct proportion to their charge transfer intensities^56^. Therefore, Cz and PXZ are further introduced at para-positions of N atom in DPPQ to establish LCT interactions, whose different electron-donating effects are utilized to modulate charge transfer intensities.

Single crystal of [DPPQ]_2_Cu_4_I_4_ contains a Cu_2_I_2_ rhombus, which indirectly connect with the rest Cu^+^ ions through the other two I^-^ ions, because the steric hindrance of DPPQ prevents its N and P atoms from chelating the same Cu^+^ ions (Suppl. Figs. 9, 10). The single crystal structures of [CzDPPQ]_2_Cu_4_I_4_ and [PXZDPPQ]_2_Cu_4_I_4_ are similar, in which four Cu^+^ ions of Cu_4_I_4_ cores form a rhomboid, owing to the steric hindrances on the sides of Cz and PXZ groups counteracting that of quinoline (Fig. 1c and Suppl. Figs. 11, 12)^57^. Besides two *µ*^2^-I atoms on opposite sides, two *µ*^3^-I atoms on top and bottom of the Cu_4_ plane respectively form two opposite pyramids together with three Cu^+^ ions, which have coplanar bases and a common Cu-Cu edge.

Compared to octahedral Cu_4_I_4_ and Cu_4_ square in [DPPy]_2_Cu_4_I_4_^54^, Cu-Cu bond lengths for four sides of Cu_4_ rhomboids in [CzDPPQ]_2_Cu_4_I_4_ and [PXZDPPQ]_2_Cu_4_I_4_ are increased by ~0.1 Å, accompanied by slightly shortened *µ*^3^-I-Cu bonds (Suppl. Table 1). Through bridging with *µ*^3^-I atoms, distances between two *µ*^3^-Cu^+^ ions at diagonal lines of Cu_4_ rhomboids are markedly decreased by ~1.0 Å, giving rise to additional Cu-Cu bonds and more uniform Cu-Cu interactions in whole metallic cores. Consequently, the combination of *µ*^2^-I and *µ*^3^-I bridges strengthens the connectivity and interactions within Cu_4_I_4_ cores^58,59^. Exclusion of lone electron pairs of nitrogen atoms from conjugation induces nearly unchanged N-Cu bond lengths of [CzDPPQ]_2_Cu_4_I_4_ and [PXZDPPQ]_2_Cu_4_I_4_. In contrast, increased lone-pair densities on phosphorus atoms involved in conjugation extension slightly elongates their P-Cu bonds by ~0.05 Å, reflecting weakened charge transfer between diphenylphosphine (DPP) groups and their directly coordinated Cu^+^ ions. Nonetheless, compared to N-coordinated *µ*^2^-Cu^+^ ions, DPP-coordinated *µ*^3^-Cu^+^ ions with markedly stronger Cu-Cu interactions involve whole Cu_4_ rhomboids in Cu-DPP charge transfer, resulting in uniform MLCT on the clusters.

Actually, the predominance of LCT and/or ILCT on outer ligands is crucial for achieving high luminescent efficiencies. Despite nearly equal P-Cu bond lengths, N-Cu bonds in [PXZDPPQ]_2_Cu_4_I_4_ with stronger PXZ donors are longer than those in [CzDPPQ]_2_Cu_4_I_4_, rendering moderately weakened MLCT for the former. Simultaneously, the orthogonality between PXZ and quinoline groups is beneficial to frontier molecular orbital (FMO) separation on the ligands of [PXZDPPQ]_2_Cu_4_I_4_, which can effectively enhance LCT. In addition, more distorted ligand configuration doubles the torsion angle τ(N-Cu-Cu-P) of [PXZDPPQ]_2_Cu_4_I_4_ to 12.5^o^ (Fig. 1d and Suppl. Table 1). In contrast to nearly coplanar coordination geometry of [CzDPPQ]_2_Cu_4_I_4_ with τ(N-Cu-Cu-P) of 6.5^o^, the markedly twisted configuration of [PXZDPPQ]_2_Cu_4_I_4_ undoubtedly enhances its structural rigidity and suppress non-radiative excited-state deactivation by structural relaxation.

Density functional theory simulation indicates that quinoline acceptors predominantly contribute to the lowest unoccupied molecular orbitals (LUMO) of all the clusters (Suppl. Fig. 13). Nonetheless, the D-A interactions on the ligands of [CzDPPQ]_2_Cu_4_I_4_ and [PXZDPPQ]_2_Cu_4_I_4_ strengthen the electron-withdrawing effect of quinoline and therefore reduce their LUMO energy levels, which is consistent with cyclic voltammetrical (CV) analysis (Suppl. Fig. 14). The highest occupied molecular orbital (HOMO) of [DPPQ]_2_Cu_4_I_4_ is distributed on its Cu_4_I_4_ core and phosphorus atoms, which is identical to the HOMO of [CzDPPQ]_2_Cu_4_I_4_ and the HOMO-3 of [PXZDPPQ]_2_Cu_4_I_4_. In contrast to Cz-dispersed HOMO-6 of [CzDPPQ]_2_Cu_4_I_4_, the HOMO of [PXZDPPQ]_2_Cu_4_I_4_ is thoroughly confined on its PXZ donors and ~0.15 eV shallower than those of the other two clusters according to CV results, which demonstrates the stronger electron-donating effect of PXZ than that of Cu_4_I_4_ core.

The situations for excited-state locations of [CzDPPQ]_2_Cu_4_I_4_ and [PXZDPPQ]_2_Cu_4_I_4_ are just opposite (Fig. 1d and Suppl. Figs. 15–17). The ground state (S_0_) → S_1_ and S_0_ → T_1_ excitations of all the clusters are attributed to the HOMO → LUMO transitions, corresponding to ^n^MLCT for [DPPQ]_2_Cu_4_I_4_ and [CzDPPQ]_2_Cu_4_I_4_ and ^n^LCT for [PXZDPPQ]_2_Cu_4_I_4_, respectively. The lowest excited state of [CzDPPQ]_2_Cu_4_I_4_ containing LCT components is the T_9_ state, whose half “hole” is dispersed on Cz donor. On the contrary, the ^n^MLCT featured lowest excited states of [PXZDPPQ]_2_Cu_4_I_4_ are the S_3_ and T_3_ states with Cu_4_I_4_-localized “holes”. Furthermore, the singlet oscillator strength of [PXZDPPQ]_2_Cu_4_I_4_ is more than 18 folds of those of [DPPQ]_2_Cu_4_I_4_ and [CzDPPQ]_2_Cu_4_I_4_, reflecting the superiority of LCT to MLCT states in radiation.

Consequently, in the case of [PXZDPPQ]_2_Cu_4_I_4_, its high-lying but less radiative ^n^MLCT states are embedded and therefore protected by its lowest and highly radiative ^n^LCT states, which are opposite to the situation of [CzDPPQ]_2_Cu_4_I_4_. Simultaneously, the uniformly distributed first nine triplet states of [CzDPPQ]_2_Cu_4_I_4_ induce its LCT-featured T_9_ state internally convert to the lower ^3^MLCT states step by step. The cascade transition mode of [CzDPPQ]_2_Cu_4_I_4_ can further involve in more high-lying singlet and triplet states, making peripheral ligands incorporated into exciton formation and conversion (Suppl. Fig. 18). In contrast, owing to their appropriate energy gaps of ~0.2 eV and different wave functions, the S_3_/T_3_ and S_1_/T_1_ states of [PXZDPPQ]_2_Cu_4_I_4_ establish the unique cross transition mode based on mutually internal conversion, vibrational coupling and three complementary ISC/RISC circles for energy convergence and population balance to its S_1_ and T_1_ states. Obviously, the transition modes are determined by spatial and energetic distributions of excited states. The case of [PXZDPPQ]_2_Cu_4_I_4_ is more beneficial to suppressing TPQ on high-lying ^n^MLCT states and utilizing 100% electro-generated excitons for dual emission from high-radiation ^n^LCT states.

### Red photoluminescence from solution-processed films of Cu_4_I_4_ bipyramids

**Fig. 2:**
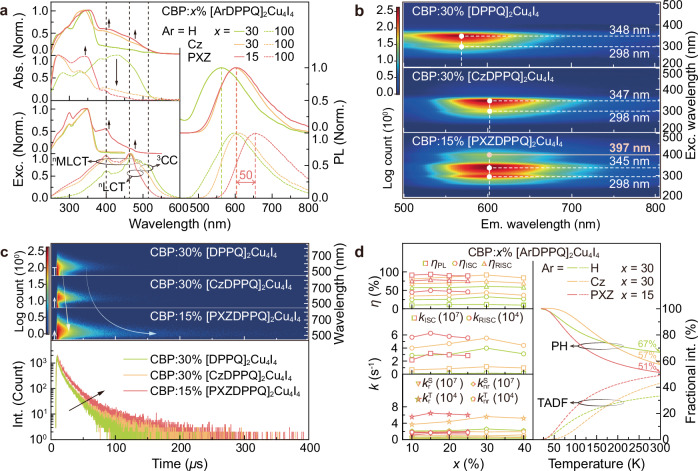
Photophysical properties of [ArDPPQ]_2_Cu_4_I_4_ based films. **a** Absorption (Abs.), excitation (Exc.) and photoluminescence (PL) spectra of CBP:*x*% [ArDPPQ]_2_Cu_4_I_4_ films (x = 100 for neat films, and 30 or 15, respectively). CBP is 4,4’-bis(9H-carbazol-9-yl)biphenyl. The spectral differences between the clusters are marked with arrows. **b** Emission-excitation mapping for [ArDPPQ]_2_Cu_4_I_4_ doped CBP films. The excitation wavelengths for emission peaks are highlighted. **c** Time-resolved emission spectra (TRES) and time decays at peak wavelengths of CBP:*x*% [ArDPPQ]_2_Cu_4_I_4_ films. The variation tendencies are marked with arrows. **d** Dependences of transition rate constants (*k*) and quantum efficiencies (*η*) on doping concentrations for CBP:*x*% [ArDPPQ]_2_Cu_4_I_4_ films in the ranges of *x* = 10–40, and temperature dependences of fractional intensities for phosphorescence (PH) and thermally activated delayed fluorescence (TADF) components of the films with optimal *x*% in the range of 20–300 K. PH proportions are presented and marked with arrows. Superscripts of “S” and “T” refer to singlet and triplet, respectively. Subscripts of “r” and “nr” refer to radiation and non-radiation, respectively.

Besides *π* → π* and n→π* absorption bands from ligands at 200–380 nm in their solutions (Suppl. Fig. 19), the neat films of the clusters exhibit additional bands from ^n^MLCT, ^n^LCT, and CC states at 400–550 nm (Fig. 2a and Suppl. Table 2). In contrast to [DPPQ]_2_Cu_4_I_4_, the charge transfer bands of [CzDPPQ]_2_Cu_4_I_4_ and [PXZDPPQ]_2_Cu_4_I_4_ neat films are inferior. But, after doped in 4,4’-bis(9H-carbazol-9-yl)biphenyl (CBP) matrix, ^n^LCT-featured bands of [PXZDPPQ]_2_Cu_4_I_4_ are stronger than ^n^MLCT bands of [CzDPPQ]_2_Cu_4_I_4_, and far beyond ^3^CC bands of [DPPQ]_2_Cu_4_I_4_. Excitation spectra of the neat films further demonstrate the predominance of CC, MLCT, and LCT states to emissions of the clusters, respectively (Suppl. Fig. 20). However, in CBP matrix, excitations on [CzDPPQ]_2_Cu_4_I_4_ and [DPPQ]_2_Cu_4_I_4_ are thoroughly based on high-lying states rather than their MLCT and CC states. On the contrary, because of its LCT-featured transition characteristics similar to CBP matrix, the S_1_ and T_1_ states of [PXZDPPQ]_2_Cu_4_I_4_ dominate the additional low-energy bands to excitation of its 15%-doped film.

Neat cluster films reveal orange-to-red emissions with peak wavelengths red shifted from 597 nm of [DPPQ]_2_Cu_4_I_4_ over 610 nm of [CzDPPQ]_2_Cu_4_I_4_ to 653 nm of [PXZDPPQ]_2_Cu_4_I_4_, in accord with the intensities of their intramolecular charge transfer interactions (Fig. 2a and Suppl. Table 2). The CC-predominant radiation of neat [DPPQ]_2_Cu_4_I_4_ film is enhanced by increasing temperature, because of MLCT-to-CC energy transfer through thermal relaxation (Suppl. Fig. 21)^60,61^. In contrast, emissions of [CzDPPQ]_2_Cu_4_I_4_ and [PXZDPPQ]_2_Cu_4_I_4_ based films are almost independent on temperature, evidencing their LC states predominant in radiation. Among CBP:*x*% clusters (*x* = 100 for neat film; < 100 for doped films), [PXZDPPQ]_2_Cu_4_I_4_ based films exhibit the largest full widths at half maximum (FWHM) more than 150 nm at *x* = 100 and the biggest bathochromic shift beyond 50 nm when increasing *x* from 10 to 100, since its outer LCT-featured S_1_ and T_1_ states facilitate the intermolecular charge transfer interactions in aggregation (Suppl. Fig. 22).

Despite identical CBP-predominant excitation characteristics, the markedly narrowed emission contour of CBP:30% [CzDPPQ]_2_Cu_4_I_4_ manifests its single-component ^n^MLCT states for radiation, in contrast to CC-predominant and MLCT-mixed states of [DPPQ]_2_Cu_4_I_4_ doped film (Fig. 2b). On the contrary, the incorporation of the high-lying states in excitation of [PXZDPPQ]_2_Cu_4_I_4_ markedly enhances photoluminescent intensities of its 15%-doped CBP film in the excitation range of 250–350 nm. More importantly, under excitation beyond 350 nm, an additional contour displays exactly the same emission peak wavelengths and profiles from [PXZDPPQ]_2_Cu_4_I_4_, corresponding to the direct excitation and radiation of its low-lying LC states. Thus, compared to its congeners, the rational excited-state composition and distribution of [PXZDPPQ]_2_Cu_4_I_4_ make its high-lying and low-lying states synergistic in radiative transitions.

Although the exposure of its LCT-featured S_1_ and T_1_ states to collisional quenching induces the shortest photoluminescent lifetimes of [PXZDPPQ]_2_Cu_4_I_4_ among the neat films, it still achieves the highest *η*_PL_, owing to the superiority of its ^n^LCT states in radiation to MLCT and CC states of its congeners (Suppl. Fig. 23 and Suppl. Table 2). CBP matrix effectively suppresses concentration quenching, markedly elongates time decays of CBP:*x*% clusters and sharply improves *η*_PL_ of [PXZDPPQ]_2_Cu_4_I_4_ doped films up to 93.6% at *x* = 15, which is record high among red cluster emitters, beyond 91.6% of [CzDPPQ]_2_Cu_4_I_4_ at *x* = 30 for the maximum, and more than 8 folds of 11.5% for [DPPQ]_2_Cu_4_I_4_ at *x* = 30 (Fig. 2c and Suppl. Fig. 23 and Suppl. Table 3). Time resolved emission spectra (TRES) further demonstrates both concentrated and prolonged components of CBP:15% [PXZDPPQ]_2_Cu_4_I_4_ are enhanced to support state-of-the-art *η*_PL_.

Singlet-triplet splitting energies (Δ*E*_ST_) of all the clusters are within 0.1 eV, which are less enough for RISC and subsequent TADF (Suppl. Fig. 24 and Suppl. Table 2). Nonetheless, compared to [DPPQ]_2_Cu_4_I_4_, donor functionalization comprehensively improves the rate constants of singlet (${k}_{{{{\rm{r}}}}}^{{{{\rm{S}}}}}$) and triplet (${k}_{{{{\rm{r}}}}}^{{{{\rm{T}}}}}$) radiations, ISC (*k*_ISC_) and RISC (*k*_RISC_) by more than about 40, 8, 6 and 2 folds for [CzDPPQ]_2_Cu_4_I_4_ and [PXZDPPQ]_2_Cu_4_I_4_ doped films. Simultaneously, in opposite to nonradiative predominance for [DPPQ]_2_Cu_4_I_4_, the ratios of ${k}_{{{{\rm{r}}}}}^{{{{\rm{S}}}}}$ to singlet nonradiative rate constant (${k}_{{{{\rm{nr}}}}}^{{{{\rm{S}}}}}$) and ${k}_{{{{\rm{r}}}}}^{{{{\rm{T}}}}}$ to triplet nonradiative rate constant (${k}_{{{{\rm{nr}}}}}^{{{{\rm{T}}}}}$) are markedly increased by at least one order of magnitude after donor modificaton (Fig. 2d and Suppl. Table 3). Compared with CBP:30% [CzDPPQ]_2_Cu_4_I_4_, the markedly larger ${k}_{{{{\rm{r}}}}}^{{{{\rm{S}}}}}$, ${k}_{{{{\rm{r}}}}}^{{{{\rm{T}}}}}$ and ${k}_{{{{\rm{r}}}}}^{{{{\rm{S}}}}}$/${k}_{{{{\rm{nr}}}}}^{{{{\rm{S}}}}}$ ratio and smaller ${k}_{{{{\rm{nr}}}}}^{{{{\rm{T}}}}}$ of CBP:15% [PXZDPPQ]_2_Cu_4_I_4_ manifest the combined radiation enhancement and quenching suppression. Furthermore, the rate constants and quantum efficienceis of both ISC and RISC transitions for CBP:15% [PXZDPPQ]_2_Cu_4_I_4_ are simultaneously the largest among its congeners. Obviously, [PXZDPPQ]_2_Cu_4_I_4_ endows its films with more efficient and balanced dual-emission characteristics.

Increasing temperature shortens time decays of CBP:*x*% clusters (*x* = 30 or 15) (Suppl. Figs. 25–27). However, the variations of [CzDPPQ]_2_Cu_4_I_4_ and [PXZDPPQ]_2_Cu_4_I_4_ doped films are markedly larger than those of [DPPQ]_2_Cu_4_I_4_ based analog, reflecting the gradual evolution from spin-forbidden PH to spin-allowed TADF for the former (Suppl. Figs. 28, 29). The largest reduction of microsecond-scale lifetimes for CBP:15% [PXZDPPQ]_2_Cu_4_I_4_ is consistent with its biggest *k*_RISC_. Furthermore, photoluminescent lifetimes of CBP:15% [PXZDPPQ]_2_Cu_4_I_4_ are always shorter than those of CBP:30% [CzDPPQ]_2_Cu_4_I_4_ at 50–300 K, manifesting the superiority of cross-transition mode for the former to cascade transition mode of the latter in simultaneous acceleration of singlet and triplet radiations.

PH and TADF proportions of CBP:*x*% clusters (*x* = 30 or 15) are quantitatively evaluated as functions of temperature, all of which are more than one third at 300 K in accord with typical dual-emissive characteristics (Fig. 2d)^37^. Since TADF from [DPPQ]_2_Cu_4_I_4_ and [CzDPPQ]_2_Cu_4_I_4_ originates from their ^1^MLCT states, ^3^CC-predominant state of [DPPQ]_2_Cu_4_I_4_ renders the biggest PH/TADF ratio of 67/33 for its doped film; while, [CzDPPQ]_2_Cu_4_I_4_ featuring ^n^MLCT predominance supports its doped film with decreased PH/TADF ratio of 57/43. In comparison, cross transition characteristics and multiple ISC-RISC circles of [PXZDPPQ]_2_Cu_4_I_4_ exactly balance singlet and triplet radiations from its ^n^LCT states, giving rise to nearly “ideal” PH/TADF ratio of 51/49 for its doped film.

It demonstrates that the state-of-the-art *η*_PL_ and equal dual-emissive characteristics of CBP:15% [PXZDPPQ]_2_Cu_4_I_4_ are ascribed to the highly radiative LCT-featured S_1_ and T_1_ states and the synergy from ^n^MLCT states in cross transition mode, which accelerate MLCT → LCT conversion and ^n^LCT radiation, and simultaneously alleviate exciton quenching on outer ^n^LCT and high-lying ^n^MLCT states.

### Red CLEDs based on Cu_4_I_4_ bipyramids

**Fig. 3:**
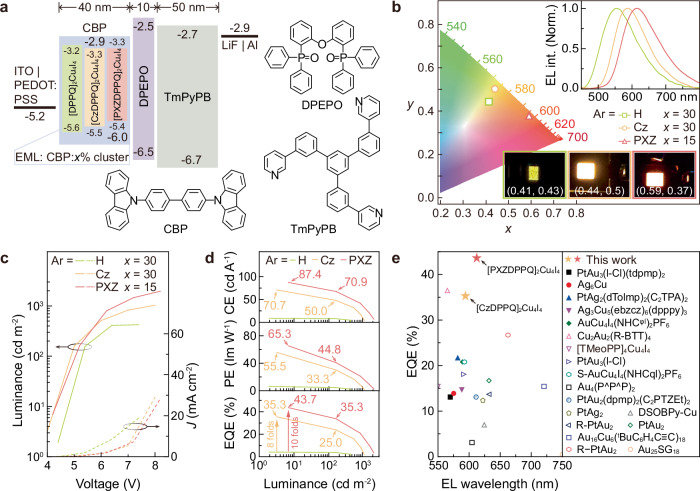
Electroluminescent (EL) performances of [ArDPPQ]_2_Cu_4_I_4_ based cluster light-emitting diodes (CLED). **a** Structures and energy level diagrams of CLEDs based on emissive layers (EML) of CBP:*x*% [ArDPPQ]_2_Cu_4_I_4_ and chemical structures of CBP, bis[2-(diphenylphosphino)phenyl]ether oxide (DPEPO), and 1,3,5-tris(3-pyridyl-3-phenyl)benzene (TmPyPB) used as host, exciton-blocking, and electron-transporting layers. **b** Commission Internationale de lEclairage (CIE) coordinates on chromatic panel of CLEDs and corresponding EL spectra and device photos at 5 V (insets). **c** Current density (*J*)-voltage-luminance characteristics of CLEDs at the optimal *x*. **d** Efficiencies*-*luminance relationships for the devices. CE, PE and EQE refer to current efficiency, power efficiency and external quantum efficiency, respectively. Arrows indicate efficiency values at the maxima and ~100 nits, respectively. **e** Comparison on the maximum EQE values of the representative EL clusters featuring orange to red emissions.

We employed CBP:*x*% clusters as emissive layers (EML) to fabricate spin-coated trilayer CLEDs (Fig. 3a). The doping concentrations were adjusted to achieve the optimal electroluminescent performances. CBP matrix and electron-transporting materials were chosen with the deeper HOMOs, the shallower LUMOs and the higher excited-state energy levels to confine the excitons on the cluster dopants, leading to the greenish yellow, orange, and red emissions respectively originated from [DPPQ]_2_Cu_4_I_4_, [CzDPPQ]_2_Cu_4_I_4_ and [PXZDPPQ]_2_Cu_4_I_4_ for their devices (Fig. 3b, Suppl. Figs. 30–32 and Suppl. Table 4). It is noted that compared to photoluminescence spectra, emission bands attributed to CBP matrix in electroluminescence spectra were markedly weakened and even disappeared, demonstrating the exciton formation on the clusters by direct carrier capture, in addition to CBP-cluster exciton transport.

Owing to their carrier injecting abilities improved by donor functionalization, [CzDPPQ]_2_Cu_4_I_4_ and [PXZDPPQ]_2_Cu_4_I_4_ reduced the turn-on voltages of their devices by 0.2–0.7 V, in comparison with [DPPQ]_2_Cu_4_I_4_ based analogs (Fig. 3c, Suppl. Figs. 30–32 and Suppl. Table 4). [PXZDPPQ]_2_Cu_4_I_4_ further endowed its devices with the maximum luminance of ~2000 nits at *x* = 15, which was about two and four folds of those of [CzDPPQ]_2_Cu_4_I_4_ and [DPPQ]_2_Cu_4_I_4_ based analogs, respectively. On account of the comparable *η*_PL_ values of [CzDPPQ]_2_Cu_4_I_4_ and [PXZDPPQ]_2_Cu_4_I_4_ and similar current densities (*J*) of their devices, the doubled maximum luminance of CBP:15% [PXZDPPQ]_2_Cu_4_I_4_ reflects the more efficient exciton formation and utilization.

More Inspiringly, besides current (CE, *η*_CE_) and power efficiency (PE, *η*_PE_) respectively up to 87.4 cd A^−^^1^ and 65.3 lm W^−1^, CBP:15% [PXZDPPQ]_2_Cu_4_I_4_ endowed its device with the state-of-the-art *η*_EQE_ as high as 43.7% (Fig. 3d, Suppl. Figs. 32, 33 and Suppl. Table 4). This result not only significantly boosted the largest *η*_EQE_ by more than one quarter for all CLEDs reported so far^62^, but also updated the *η*_EQE_ record for all kinds of red planar light-emitting devices without additional out-coupling enhancement (Fig. 3e and Suppl. Table 5)^63^. Compared to [DPPQ]_2_Cu_4_I_4_ based analogs, [PXZDPPQ]_2_Cu_4_I_4_ supported its devices with tenfold increased *η*_EQE_. Meanwhile, despite the top-rank efficiencies of CBP:30% [CzDPPQ]_2_Cu_4_I_4_ reaching 70.7 cd A^−1^, 55.5 lm W^−1^ and 35.3%, its maximum *η*_EQE_ was still one-fifth lower than that of CBP:15% [PXZDPPQ]_2_Cu_4_I_4_. According to energy gap rule, red electroluminescent materials often suffered from more serious triplet quenching. Nonetheless, [PXZDPPQ]_2_Cu_4_I_4_ effectively improved the efficiency stability of its devices. The *η*_EQE_ of CBP:15% [PXZDPPQ]_2_Cu_4_I_4_ at 100 nits remained as high as 35.3%, which was equal to the maximum value of [CzDPPQ]_2_Cu_4_I_4_ based analogs. In contrast, *η*_EQE_ of CBP:30% [CzDPPQ]_2_Cu_4_I_4_ sharply decreased to 25.0% at 100 nits, corresponding to the EQE roll-off 50% larger than that of CBP:15% [PXZDPPQ]_2_Cu_4_I_4_.

Because *η*_PL_ values and electrical properties of CBP:30% [CzDPPQ]_2_Cu_4_I_4_ and CBP:15% [PXZDPPQ]_2_Cu_4_I_4_ were similar, it is convincing that accurately optimized excited state characteristics of the latter should be one of the determinants resulting in the sharply increased electroluminescent efficiencies. Actually, compared to other kinds of red electroluminescent materials (Suppl. Table 6), the merit of cluster emitters is fully embodied by [PXZDPPQ]_2_Cu_4_I_4_ in synergy and complementarity between multiple excited states involved in exciton formation and utilization.

### Exciton kinetics in CLEDs

**Fig. 4:**
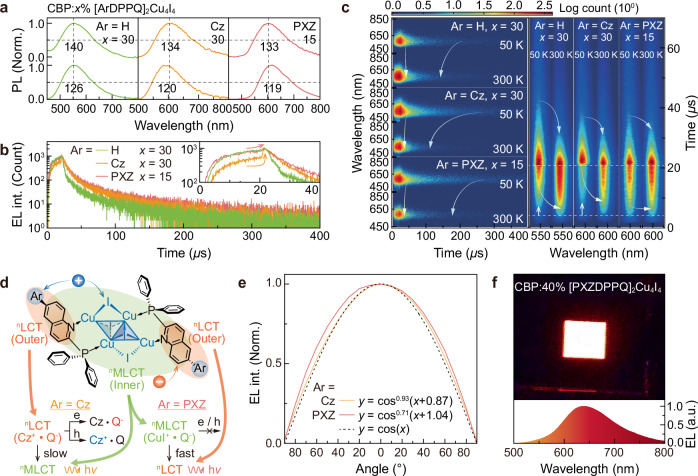
Exciton kinetics of [ArDPPQ]_2_Cu_4_I_4_ based CLEDs. **a** Comparison on PL and EL spectra of CBP:*x*% [ArDPPQ]_2_Cu_4_I_4_ in film and EML. The variations of full widths at half maximum (FWHM) are presented to indicate the influences of excited states in exciton harvesting. **b** EL time decays of the devices. The turning periods from carrier recombination (0–20 *µ*s) to exciton decay (20–40 *µ*s) are highlighted in inset. Arrows indicate the intensity variation tendencies at the ends of electrical pulse. **c** Time-resolved EL emission spectra (TREES, left) and sliced contours covering turning periods in the range of 0–40 *µ*s at 50 and 300 K. Arrows indicate the variations after temperature increasing from 50 to 300 K. **d** Proposed EL mechanisms of exciton-polaron interactions at [ArDPPQ]_2_Cu_4_I_4_ based on the different characteristics of their S_1_ and T_1_ states and the different locations of ^n^MLCT and ^n^ILCT states. **e** Angular distributions of electroluminescence intensity for the devices fitted with *y* = cos^n^(*x* + *a*). *y* and *x* refer to intensity and measuring angle. *a* is a constant. *n* = 1 for Lambertian emission, < 1 for super-Lambertian emission. **f** EL spectrum and photo at 5 V of a deep-red device based on CBP:40% [PXZDPPQ]_2_Cu_4_I_4_.

The excited states of the clusters predominant in electroluminescence are basically consistent with those in photoluminescence, since similar emission bathochromic shifts of CBP:*x*% [PXZDPPQ]_2_Cu_4_I_4_ markedly larger than those of [DPPQ]_2_Cu_4_I_4_ and [CzDPPQ]_2_Cu_4_I_4_ based analogs, when *x*% increasing (Suppl. Figs. 30–32 and Suppl. Table 4). Furthermore, electroluminescent FWHM values of CBP:*x*% clusters were reduced by ~15 nm in comparison to their photoluminescence, reflecting less excited states contributing to electroluminescence (Fig. 4a and Suppl. Fig. 34). Simultaneously, different to CBP:30% [DPPQ]_2_Cu_4_I_4_ with the same photoluminescent and electroluminescent peak wavelengths, CBP:30% [CzDPPQ]_2_Cu_4_I_4_ displayed a blue shift of 17 nm for its electroluminescence. In contrast, compared to its photoluminescence, electroluminescent peak of CBP:15% [PXZDPPQ]_2_Cu_4_I_4_ shifted red by 13 nm, making [PXZDPPQ]_2_Cu_4_I_4_ more superior in improving color purity of red CLEDs.

It is rational that the reduced contributions from low-lying CC states of [DPPQ]_2_Cu_4_I_4_ and [CzDPPQ]_2_Cu_4_I_4_ at longer wavelengths and high-lying MLCT states of [PXZDPPQ]_2_Cu_4_I_4_ at shorter wavelengths resulted in their electroluminescent spectral narrowing and shifts, because the former were more sensitive than primary radiative MLCT states of [DPPQ]_2_Cu_4_I_4_ and [CzDPPQ]_2_Cu_4_I_4_ to complicated carrier-exciton-incorporated quenching effects during electroluminescence; while, the conversion of the latter to LCT states of [PXZDPPQ]_2_Cu_4_I_4_ was accelerated by carrier capture^39^. Nonetheless, because the T_1_ energy level of ~2.6 eV for CBP is 0.3-0.5 eV higher than those of the clusters, let alone ~3.0 eV of the electron transporting materials, not only the S_1_ and T_1_ states but also the high-lying excited states of the cluster dopants, especially LCT states of [CzDPPQ]_2_Cu_4_I_4_ and MLCT states of [PXZDPPQ]_2_Cu_4_I_4_, would be involved in exciton formation and transport.

We performed transient electroluminescence measurement to figure out the correlations between excited state characteristics of the clusters and exciton quenching mechanisms in their devices (Fig. 4b and Suppl. Fig. 35). The electroluminescence intensities of CBP:*x*% clusters generated by bias pulses (5 V, 20 ms) was collected as functions of time. Different to immediately decreased intensities after removing voltage for [DPPQ]_2_Cu_4_I_4_ and [PXZDPPQ]_2_Cu_4_I_4_ based analogs, a significant delayed spike at the end of the bias pulse on CBP:30% [CzDPPQ]_2_Cu_4_I_4_ corresponded to fivefold increased intensity (inset in Fig. 4b), which was attributed to electroluminescence recovery under zero current and manifested the more marked TPQ effect in [CzDPPQ]_2_Cu_4_I_4_ based devices.

Time-resolved electroluminescence emission spectra (TREES) further indicate that CBP:30% [CzDPPQ]_2_Cu_4_I_4_ revealed sharply reduced emission intensities at the carrier recombination stage when decreasing temperature; while the limited temperature influence on emission intensities of CBP:15% [PXZDPPQ]_2_Cu_4_I_4_ at this stage were the smallest (Fig. 4c and Suppl. Figs. 36 and 37). This verifies that excitons were mainly formed on the thermodynamically advantageous S_1_ and T_1_ states of [PXZDPPQ]_2_Cu_4_I_4_, but the dynamically advantageous high-lying states of [CzDPPQ]_2_Cu_4_I_4_. Furthermore, at 300 K, carrier recombination in CBP:30% [CzDPPQ]_2_Cu_4_I_4_ was delayed by ~1 *µ*s in comparison to [DPPQ]_2_Cu_4_I_4_ and [PXZDPPQ]_2_Cu_4_I_4_ based analogs. But, the carrier recombination time in CBP:15% [PXZDPPQ]_2_Cu_4_I_4_ was temperature independent, and ~3 and 5 *µ*s (at ≤ 50 K) earlier than that in [DPPQ]_2_Cu_4_I_4_ and [CzDPPQ]_2_Cu_4_I_4_ based devices, respectively, reflecting the combined advantages of [PXZDPPQ]_2_Cu_4_I_4_ in facile carrier recombination and prompt exciton radiation.

So, the integrated spatial and energetic characteristics of the excited states for the clusters result in the exciton kinetic features of their devices (Fig. 4d). Outer ^n^LCT states of [PXZDPPQ]_2_Cu_4_I_4_ are the lowest S_1_ and T_1_ excited states beneficial to carrier recombination and radiation acceleration; meanwhile, its inner and high-lying ^n^MLCT states are encapsulated by ^n^LCT states to prevent interactions with polarons for TPQ suppression, and incorporated into cross transitions for efficient and balanced dual emissions. On the contrary, outer and high-lying ^n^LCT states of [CzDPPQ]_2_Cu_4_I_4_ are non-radiative, but predominant in carrier recombination and the subsequent energy transfer to its inner and ^n^MLCT-featured S_1_ and T_1_ states, leading to its delayed radiation and more pronounced TPQ.

In addition, the angular electroluminescence distributions of [PXZDPPQ]_2_Cu_4_I_4_ and [CzDPPQ]_2_Cu_4_I_4_ based devices were in accord with forward-dominant super-Lambertian emission (Fig. 4e), which was consistent with the increased horizontal dipole orientation ratios of the corresponding films (Suppl. Fig. 38). Nonetheless, the forward out-coupling proportion of CBP:15% [PXZDPPQ]_2_Cu_4_I_4_ was markedly larger than that of CBP:30% [CzDPPQ]_2_Cu_4_I_4_, reflecting the higher out-coupling ratio of the former. On account of nearly the same *η*_PL_ values and comparable electrical properties for [CzDPPQ]_2_Cu_4_I_4_ and [PXZDPPQ]_2_Cu_4_I_4_ doped films, the one-fifth larger *η*_EQE_ of [PXZDPPQ]_2_Cu_4_I_4_ based devices should be attributed to the combined superiorities in exciton utilization and light extraction. The duration of CBP:15% [PXZDPPQ]_2_Cu_4_I_4_ based devices was sixfold longer than that of commercial Ir^3+^ phosphor, further verifying the potential for practical applications (Suppl. Fig. 39). The competence of Cu_4_I_4_ clusters for deep-red devices with higher color purity was also demonstrated by CBP:40% [PXZDPPQ]_2_Cu_4_I_4_ with electroluminescent peak wavelength beyond 630 nm (Fig. 4f).

## Discussion

We break through the bottleneck in red CLEDs with [PXZDPPQ]_2_Cu_4_I_4_ containing Cu_4_I_4_ bipyramid and conjugation-extended D-A ligands, which realized the red electroluminescence peaked at > 600 nm and the new *η*_EQE_ record of 43.7% for all kinds of planar red light-emitting devices. Compared to [CzDPPQ]_2_Cu_4_I_4_ with the similar *η*_PL_ ( > 90%) and electrical properties but reverse excited-state location, the outer LCT-featured S_1_ and T_1_ states of [PXZDPPQ]_2_Cu_4_I_4_ accelerate carrier recombination and exciton radiation; meanwhile, its inner high-lying MLCT states are protected from TPQ effect, and further provide cross transition channels to achieve the exactly balanced dual emissions (TADF/PH = 51/49). As a result, [PXZDPPQ]_2_Cu_4_I_4_ markedly increased *η*_EUE_ of its device by one-fifth. The results demonstrate the unique advantages of cluster molecules in excited-state and exciton engineering and the potential of cluster materials for next-generation full-color displays and high-quality light source.

## Methods

### Electroluminescence analysis

The devices were fabricated through firstly spin-coating CBP:*x*% clusters as emissive layers, and then respectively vacuum depositing electron transporting and injecting layers and aluminum cathodes. After then, the devices were encapsulated with glass covers and UV glue for measurement. Electroluminescence spectra were recorded with FLS 1000 equipped with a Tektronix AFG3022G function generator and a temperature controller for 11–500 K variation. Time-resolved emission spectra were measured with Time-Correlated Single Photon Counting (TCSPC) method.

### Reporting summary

Further information on research design is available in the Nature Portfolio Reporting Summary linked to this article.

## Supplementary information

Supplementary Information

Reporting summary

Transparent Peer Review file

## Source data

Source data

## Data Availability

The experimental data supporting the findings of this study are available within the Article and its Supplementary Information. The underlying data for all plots and graphs presented in Figs. 2–4 and Supplementary Figs. are provided in the Source Data file with this paper. Any additional data are available from the corresponding author upon reasonable request. The X-ray crystallographic data of [DPPQ]_2_Cu_4_I_4_, [CzDPPQ]_2_Cu_4_I_4_ and [PXZDPPQ]_2_Cu_4_I_4_ have been deposited in the Cambridge Crystallographic Data Centre (CCDC) under deposition number 2535926 [10.5517/ccdc.csd.cc2r3v2t], 2494716 [10.5517/ccdc.csd.cc2pqyq3] and 2494717 [10.5517/ccdc.csd.cc2pqyr4]. Source data are provided with this paper.
