# Supplementary material for: Red electroluminescent Cu4I4 bipyramids with external quantum efficiency beyond 40%
Source: Nat Commun. 2026 Jul 7;17:8380. doi: 10.1038/s41467-026-75381-2 (PMC13473432; doi:10.1038/s41467-026-75381-2)
Supplement: Supplementary file 1 [file 41467_2026_75381_MOESM1_ESM.pdf]

Supplementary Information

Red Electroluminescent Cu_4I_4 Bipyramids with External Quantum Efficiency beyond 40%

Jie Ren,[1] Xiudan Song,[2] Chunlei Zhong,[1] Jianan Sun,[1] Xinjie Wang,[1] Chunbo Duan,[1] Chunmiao Han,[1] Ying Wei,[3] and Hui Xu[1,*]

[1]Department of Applied Chemistry, School of Chemistry and Material Science, Heilongjiang University, 74 Xuefu Road, Harbin 150080, China.

[2]School of Physical Science and Technology, Heilongjiang University, 74 Xuefu Road, Harbin 150080, China.

[3]Department of Electrical Engineering, School of Mechanical and Electrical Engineering, Heilongjiang University, 74 Xuefu Road, Harbin 150080, China.

*Correspondence to: hxu@hlju.edu.cn (HX).

Contents

Supplementary Note 1. Materials and Synthesis Method S2

Supplementary Note 2. Physical Measurements and Structures..................................... S8

Supplementary Note 3. Theoretical Simulation S15

Supplementary Note 4. Photophysical Analysis S21

Supplementary Note 5. Electroluminescence Analysis S35

Supplementary References S56

Supplementary Note 1. Materials and Synthesis Method

Reagents and solvents for syntheses were purchased from Aldrich and Alfa-Aesar. All chemicals were used without further purification.

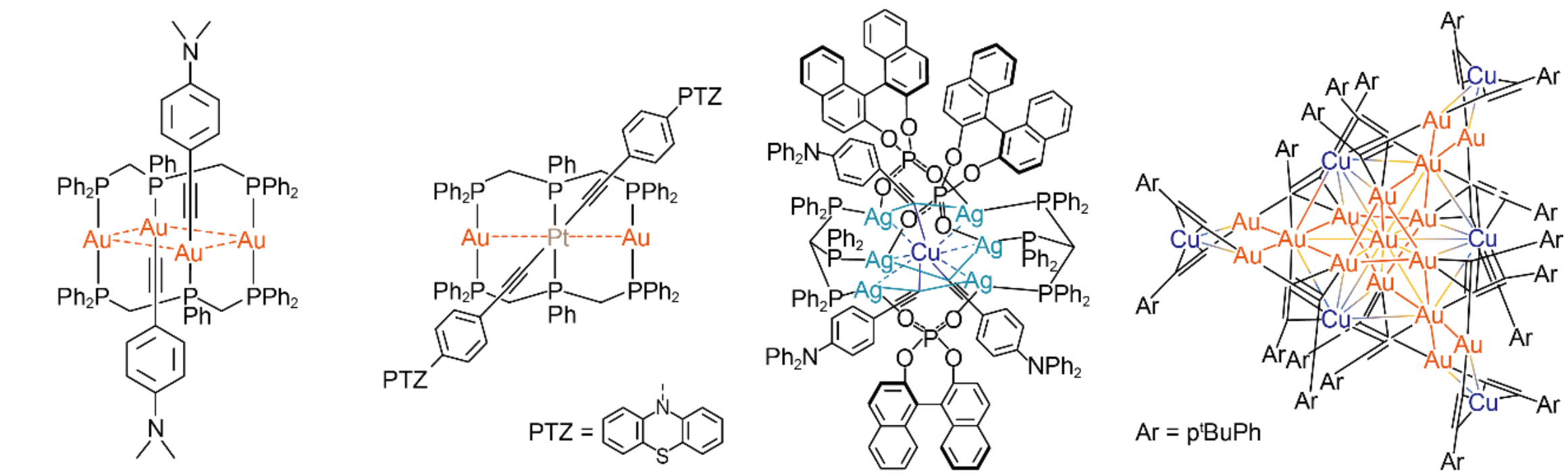

$[Au_4(P\text{^}P\text{^}P)_2(C_2PhNMe_2)_2]^{2+}$	$PtAu_2(dpmp)_2(C{\equiv}C\text{-PTZ-Et})_2$	R-Au_6Cu	$Au_{16}Cu_6(tBuC_6H_4C{\equiv}C)_{18}$
IC **2014**, *53*, 12720.	*JMCC* **2016**, *4*, 6096.	*AC* **2025**, *64*, e202417934.	*AC* **2025**, *64*, *e202508578.*
$\lambda_{PL/EL}$ = 676/600 nm	$\lambda_{PL/EL}$ = 684/606 nm	$\lambda_{PL/EL}$ = 666/653 nm	$\lambda_{PL/EL}$ = 706/706 nm
$\eta_{PL/EL}$ = 90.3%/3.1%	$\eta_{PL/EL}$ = 73.7%/13.1%	$\eta_{PL/EL}$ = 90.3%/26.7%	$\eta_{PL/EL}$ = 76.9%/15.4%

Supplementary Fig. 1 | Photoluminescence (PL) and electroluminescence (EL) peak wavelengths (λ) and quantum efficiencies (η) of representative red cluster emitters.

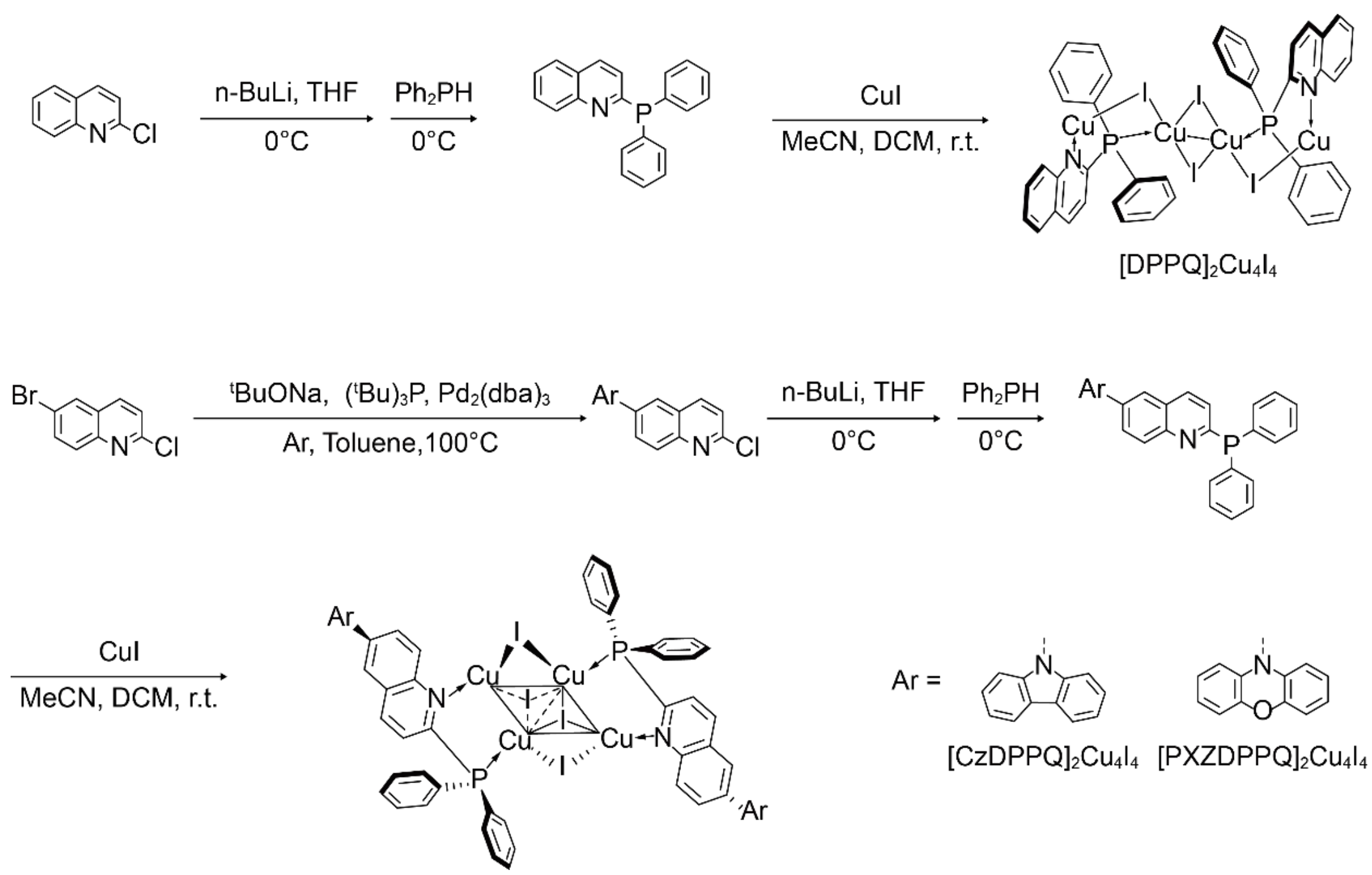

Supplementary Fig. 2 | Synthetic procedures of $[DPPQ]_2Cu_4I_4$, $[CzDPPQ]_2Cu_4I_4$ and $[PXZDPPQ]_2Cu_4I_4$.

9-(2-Chloroquinolin-6-yl)-9H-carbazole: 2-chloro-6-bromoquinoline (0.218 g, 0.9 mmol), carbazole (0.167 g, 1.0 mmol), sodium tert-butoxide (0.125 g, 1.3 mmol), $Pd(dba)_2$ (dba = dibenzylideneacetone, 9.2 mg, 1.0 mmol) and tri-tert-butylphosphine (0.235 mL, 1.2 mmol) were dissolved in toluene (5 mL), and the solution was stirred for 12 h at 100 ^oC under argon atmosphere. Then, the system was naturally cooled to room temperature, and the reaction was quenched by water addition. The mixture was extracted with dichloromethane, and purified with column chromatography to obtain white solid of 0.25 g with a yield of 85%. ^1H NMR (400 MHz, $CDCl_3$): δ = 8.24 (d, *J* = 8.9 Hz, 1H), 8.16 (d, *J* = 7.5 Hz, 3H), 8.02 (s, 1H), 7.96 (d, *J* = 8.9 Hz, 1H), 7.48 (d, *J* = 8.4 Hz, 1H), 7.44 (t, *J* = 7.0 Hz, 4H), 7.32 ppm (t, *J* = 7.0 Hz, 2H).

10-(2-Chloroquinolin-6-yl)-10H-phenoxazine: the synthetic procedure was the same with 9-(2-chloroquinolin-6-yl)-9H-carbazole, except for using phenoxyazine (0.183 g, 1.0 mmol) instead of carbazole. The residue was purified with column chromatography to obtain white solid of 0.30 g with a yield of 87%. ^1H NMR (400 MHz, $CDCl_3$): δ = 8.24 (d, *J* = 8.9 Hz, 1H), 8.13 (d, *J* = 8.6 Hz, 1H), 7.86 (d, *J* = 1.8 Hz, 1H), 7.68 (dd, *J* = 8.8, 2.0 Hz, 1H), 7.46 (d, *J* = 8.6 Hz, 1H), 6.74 – 6.70 (m, 2H), 6.67 (t, *J* = 7.5 Hz, 2H), 6.57 (dd, *J* = 11.0, 4.2 Hz, 2H), 5.89 (d, *J* = 7.8 Hz, 2H).

2-Diphenylphosphine (DPPQ): In Ar, n-butyllithium (2.5 M in hexane, 0.54 mL, 1.0 mmol) was added into a tetrahydrofuran solution (5 mL) of diphenylphosphine (0.20 mL, 3.5 mmol) at 0 ^oC in dropwise. The system was reacted under the same temperature for 0.5 h, and then the resulting solution was slowly added to a tetrahydrofuran solution (5 mL) of 2-chloroquinoline (0.16 g, 1.0 mmol) at 0 °C under an argon atmosphere and stirred for 4 h. The system was then allowed to warm to room temperature naturally. After adding water, the reaction was quenched. The system was extracted with dichloromethane, and purified by column chromatography to obtain a white solid of 0.25 g with a yield of 86%. ^1H NMR (400 MHz, $CDCl_3$): δ = 8.17 (d, J = 8.5 Hz, 1H), 8.03 (d, J = 8.4 Hz, 1H), 7.80 (d, J = 8.1 Hz, 1H), 7.73 (t, J = 7.3 Hz, 1H), 7.55 (t, J = 7.4 Hz, 1H), 7.50 – 7.44 (m, 4H), 7.39 (d, J = 4.8 Hz, 6H), 7.21 ppm (d, J = 8.4 Hz, 1H).

2-Diphenylphosphine-6-carbazolequinoline (CzDPPQ): the synthetic procedure was the same with DPPQ, except for using 9-(2-chloroquinolin-6-yl)-9H-carbazole (0.330 g, 1.0 mmol) instead of 2-chloroquinoline. The residue was purified by column

chromatography to obtain a white solid of 0.35 g with a yield of 73%. ^{1}H NMR (400 MHz, $CDCl_3$): δ = 8.26 (d, J = 8.9 Hz, 1H), 8.09 (d, J = 7.6 Hz, 2H), 7.97 (d, J = 8.5 Hz, 1H), 7.90 (d, J = 2.2 Hz, 1H), 7.83 (dd, J = 8.9, 2.3 Hz, 1H), 7.41 (ddd, J = 11.8, 7.0, 3.4 Hz, 7H), 7.37 – 7.30 (m, 9H), 7.24 (s, 1H), 7.23 – 7.20 (m, 1H), 7.18 ppm (d, J = 5.1 Hz, 1H).

2-Diphenylphosphine-6- pyrazinoquinoline (PXZDPPQ): the synthetic procedure was the same with DPPQ, except for using 10-(2-chloroquinolin-6-yl)-10H-phenoxazine (0.345 g, 1.0 mmol) instead of 2-chloroquinoline. The residue was purified by column chromatography to obtain a white solid of 0.35 g with a yield of 71%. ^{1}H NMR (400 MHz, $CDCl_3$): δ = 8.25 (d, J = 8.9 Hz, 1H), 7.94 (d, J = 7.5 Hz, 1H), 7.74 (d, J = 2.1 Hz, 1H), 7.56 (dd, J = 8.9, 2.2 Hz, 1H), 7.44 – 7.38 (m, 4H), 7.32 (dd, J = 3.7, 2.8 Hz, 6H), 7.17 (d, J = 8.1 Hz, 2H), 6.67 – 6.63 (m, 2H), 6.59 (td, J = 7.6, 1.4 Hz, 2H), 6.50 (td, J = 7.7, 1.6 Hz, 2H), 5.87 ppm (dd, J = 7.9, 1.3 Hz, 2H).

$[DPPQ]_2Cu_4I_4$: CuI (0.38 g, 2 mmol) was dissolved in 5 ml of MeCN, and then transferred to a test tube after filtration. Then, 5 ml of blank MeCN was added in dropwise to form a buffer layer. Finally, a layer of DPPQ (0.29 g, 1 mmol) in 5 mL of CH_2Cl_2 was added in dropwise. The system was standing for several days to afford single crystals of the title compound. ^{1}H NMR (400 MHz, $CDCl_3$): δ = 8.03 (d, J = 8.8 Hz, 16H), 7.95 – 7.90 (m, 4H), 7.40 ppm (s, 12H). CCDC No. 2535926.

$[CzDPPQ]_2Cu_4I_4$: In Ar, CzDPPQ (0.48 g, 1.0 mmol) and CuI (0.38 g, 2.0 mmol) were dispersed in 5 mL of CH_2Cl_2. The mixture was stirred for 4 h at room temperature. Then, the solvent was evaporated to obtain crude product, which was further recrystallized from CH_2Cl_2/methanol solution to afford yellowish crystals of 1.5 g with a yield of 88%. ^{1}H NMR (400 MHz, $CDCl_3$): δ = 8.32 (d, J = 8.7 Hz, 2H), 8.09 (d, J = 7.3 Hz, 6H), 8.02 (s, 4H), 7.99 – 7.94 (m, 4H), 7.45 – 7.36 (m, 28H), 7.29 – 7.26 (m, 6H). CCDC No. 2494716.

$[PXZDPPQ]_2Cu_4I_4$: the synthetic procedure was the same with $[CzDPPQ]_2Cu_4I_4$, except for using PXZDPPQ (0.49 g, 1.0 mmol) instead of CzDPPQ. The crude product was further recrystallized from CH_2Cl_2/ether solution to afford yellow crystals of 1.6 g with a yield of 91%. ^{1}H NMR (400 MHz, $CDCl_3$): δ = 8.17 (s, 2H), 7.84 (s, 2H), 7.63 (s, 8H), 7.40 (s, 12H), 6.70 – 6.58 (m, 12H), 6.51 (dd, J = 16.6, 9.4 Hz, 6H), 5.88 (t, J = 10.2 Hz, 6H). CCDC No. 2494717.

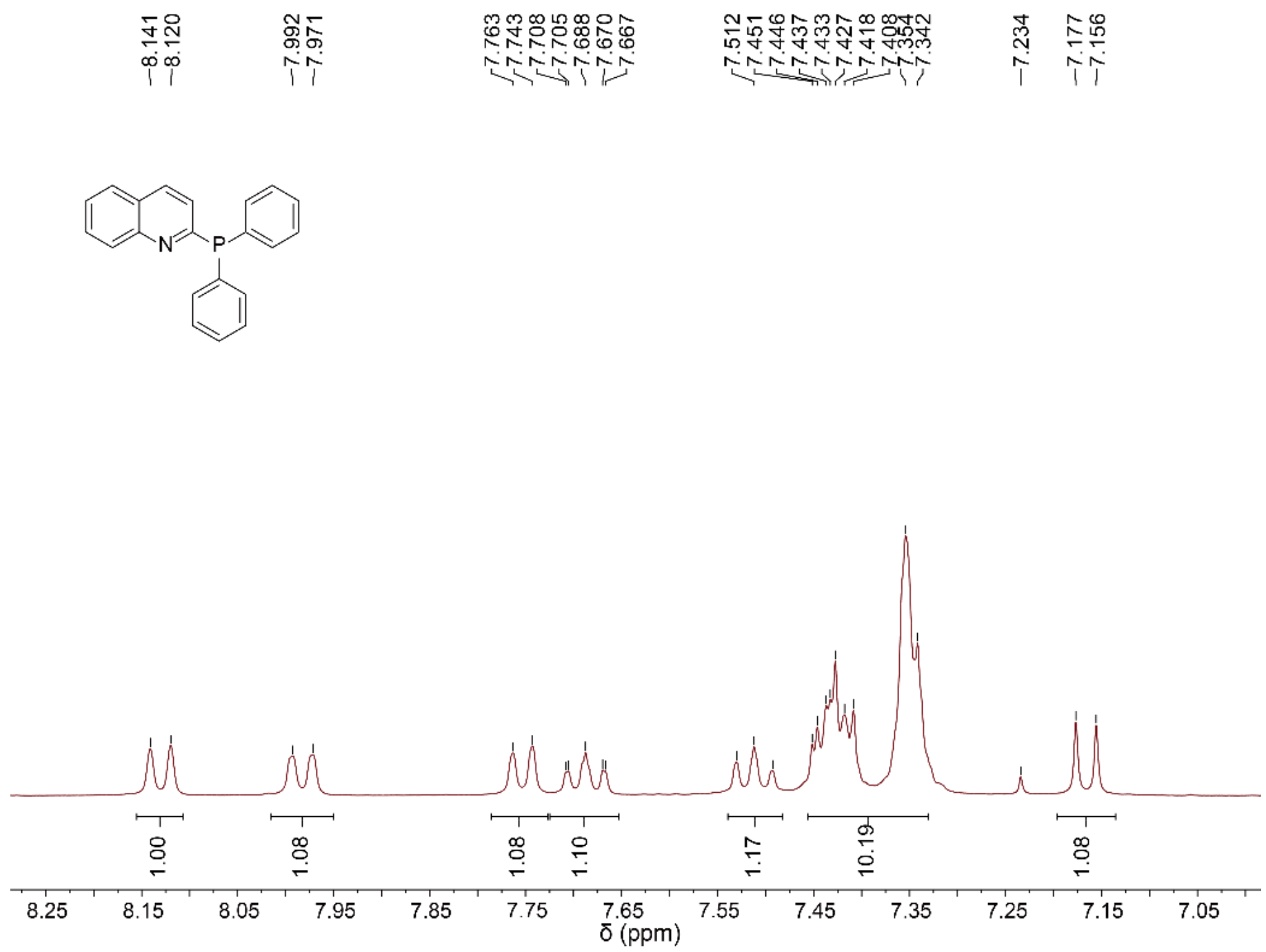

Supplementary Fig. 3 | ^{1}H NMR spectrum of DPPQ in $CDCl_3$.

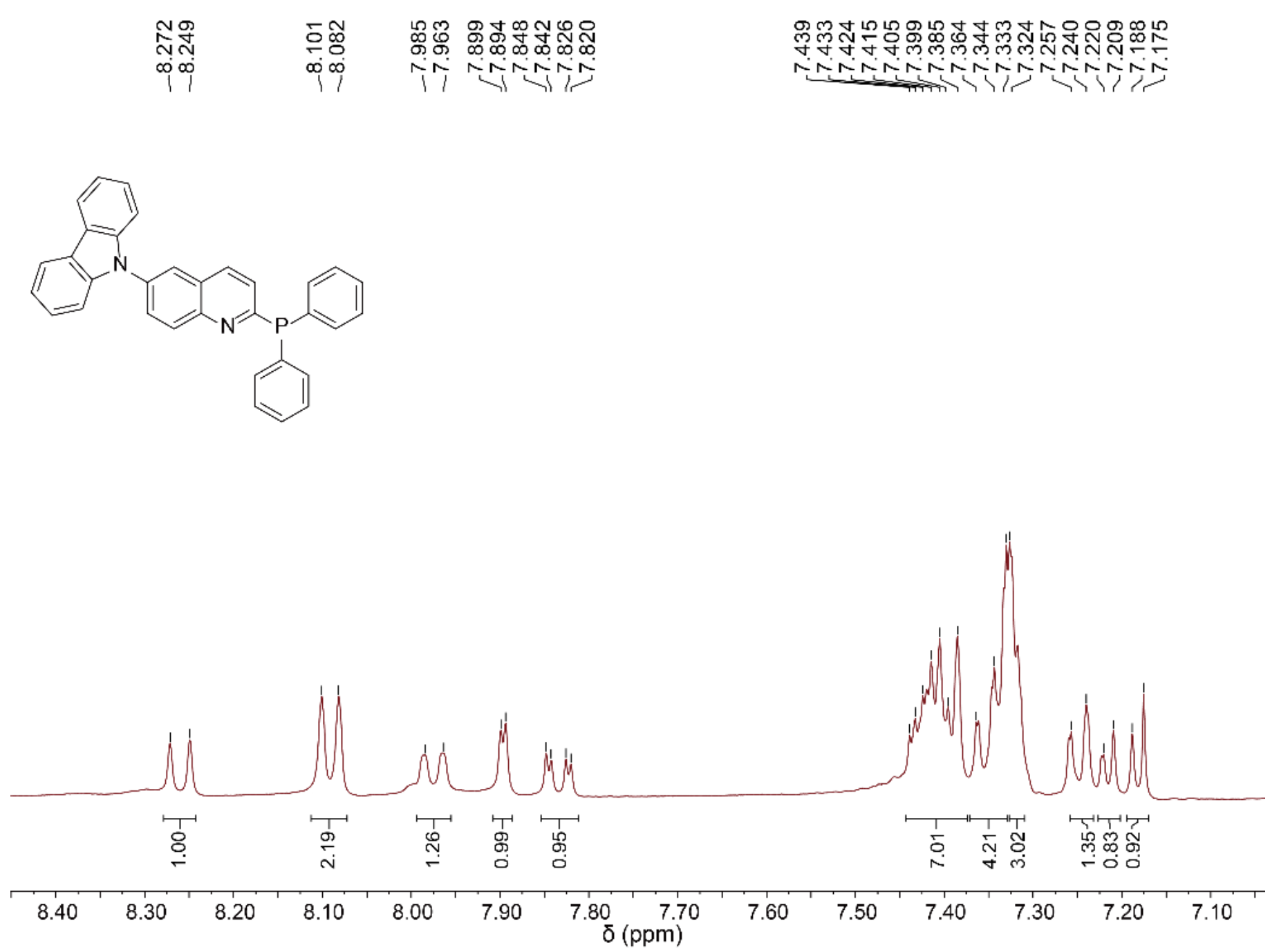

Supplementary Fig. 4 | ^{1}H NMR spectrum of CzDPPQ in $CDCl_3$.

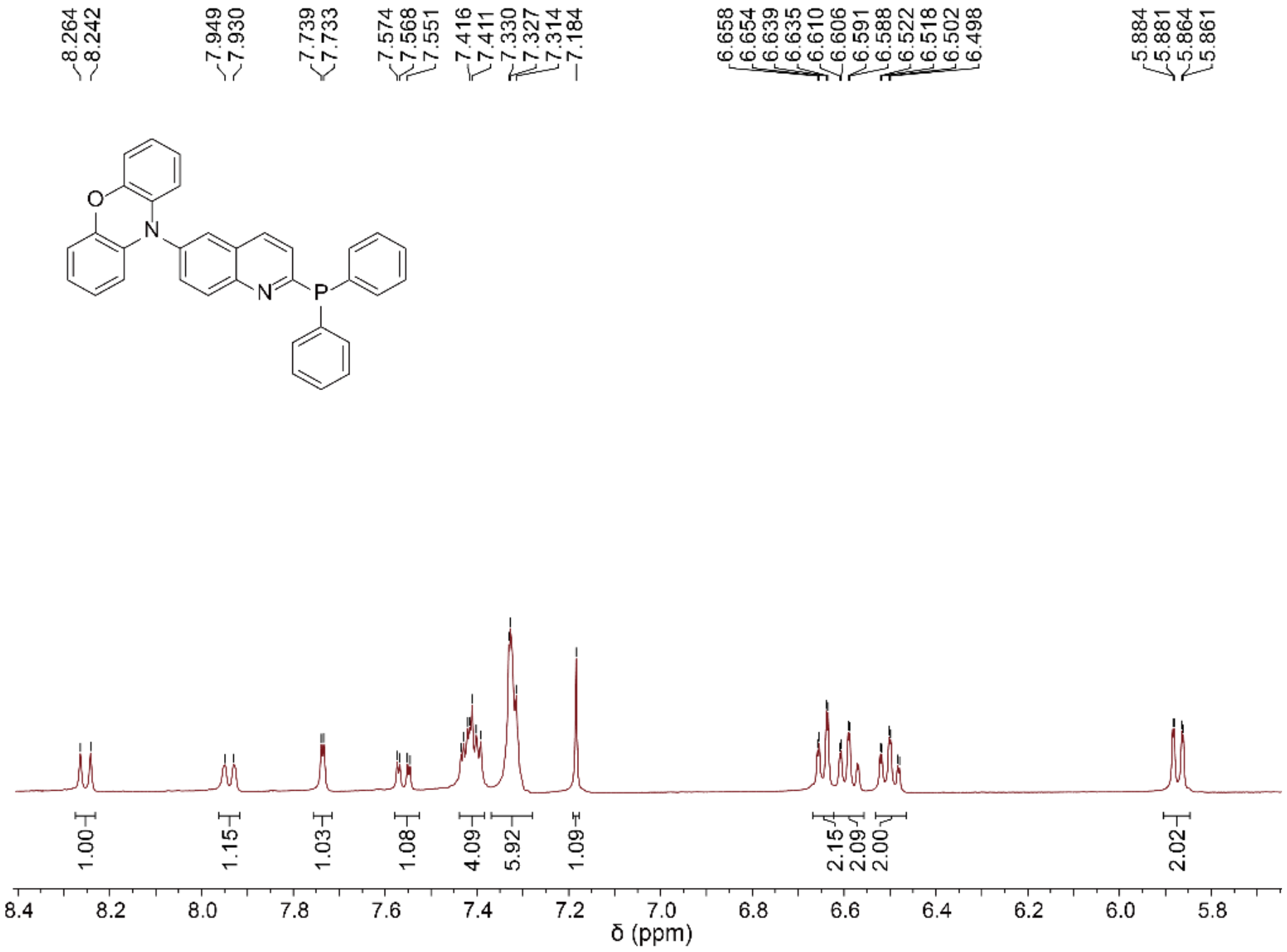

Supplementary Fig. 5 | ^{1}H NMR spectrum of PXZDPPQ in $CDCl_3$.

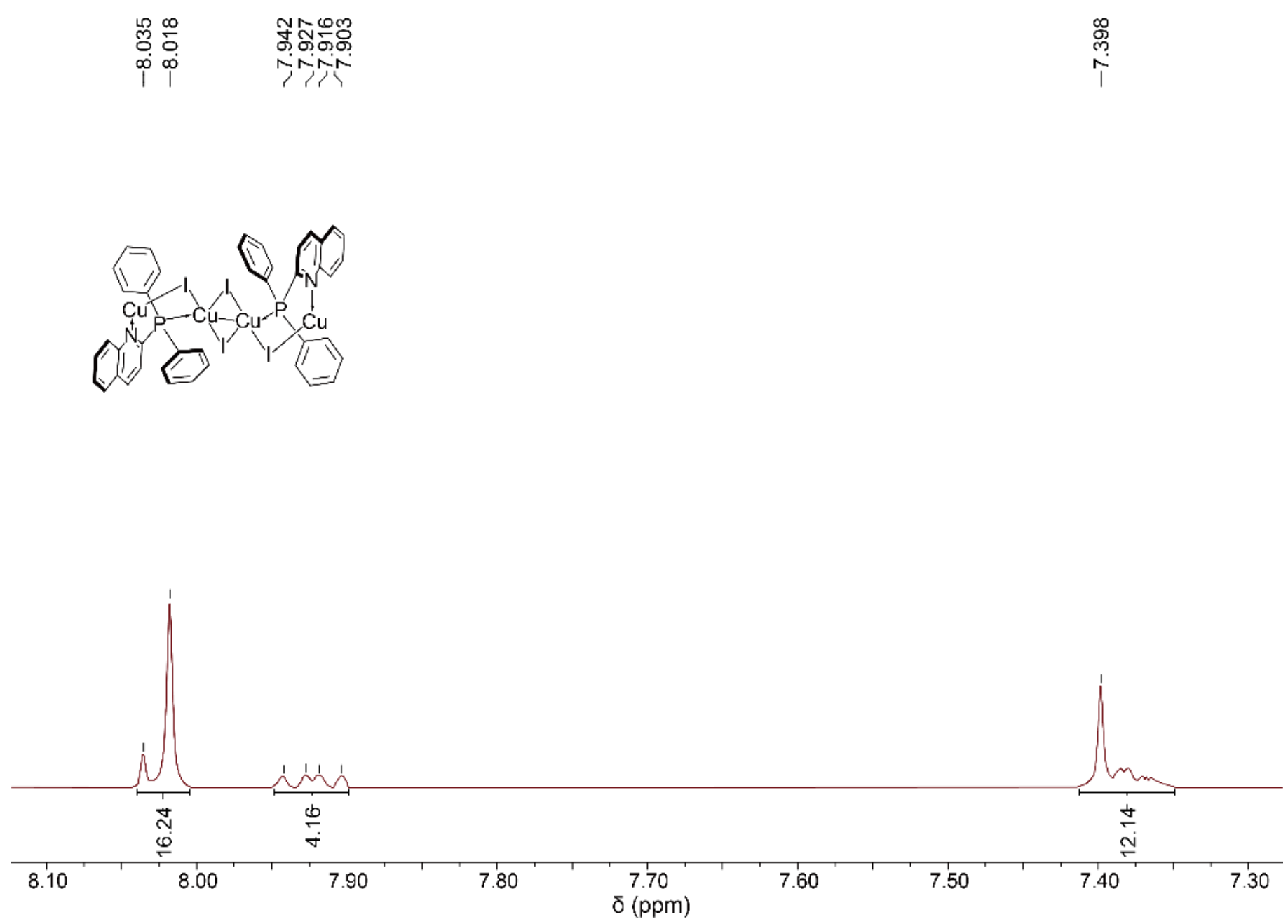

Supplementary Fig. 6 | ^{1}H NMR spectrum of $[DPPQ]_2Cu_4I_4$ in $CDCl_3$.

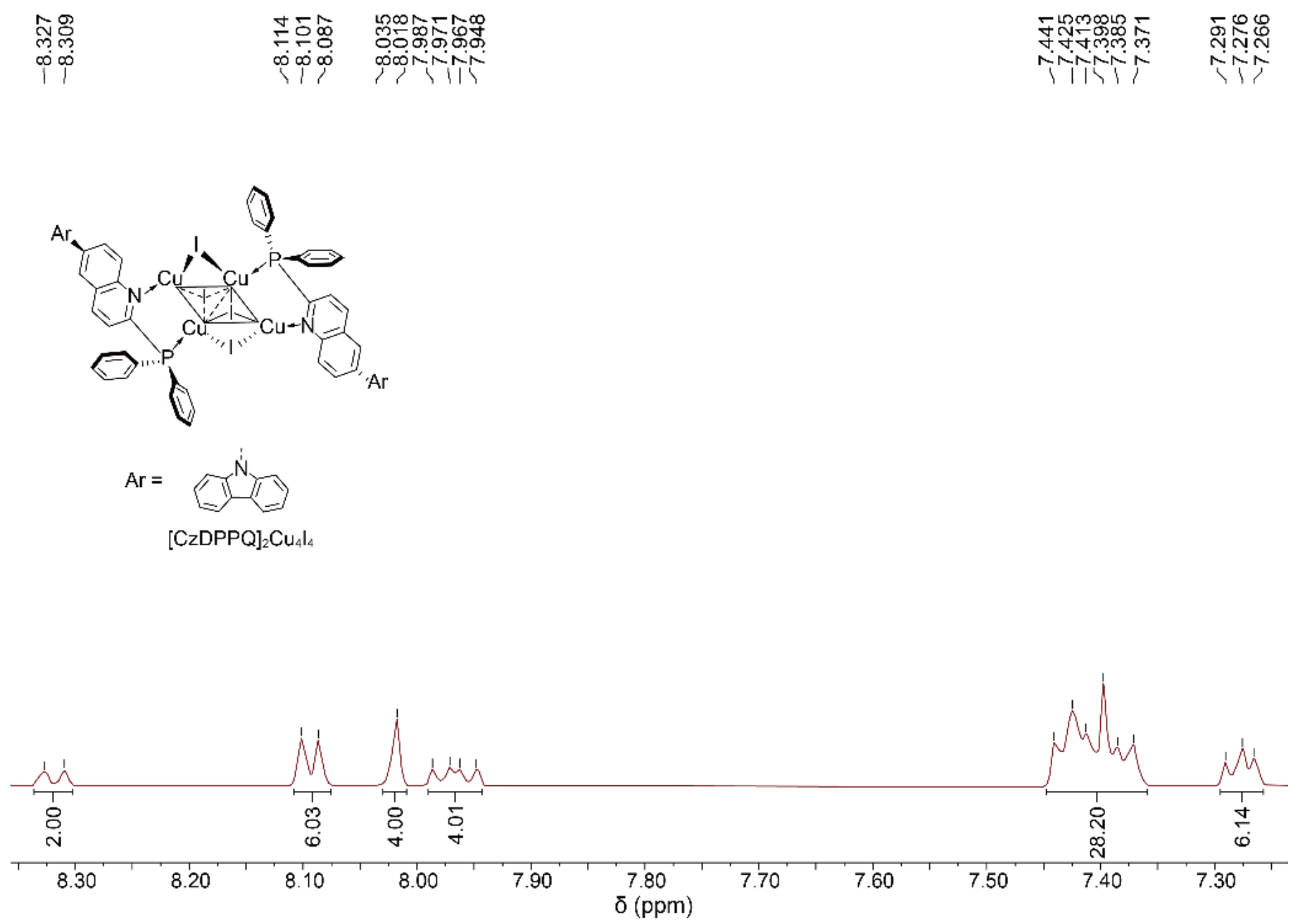

Supplementary Fig. 7 | ^{1}H NMR spectrum of $[CzDPPQ]_2Cu_4I_4$ in $CDCl_3$.

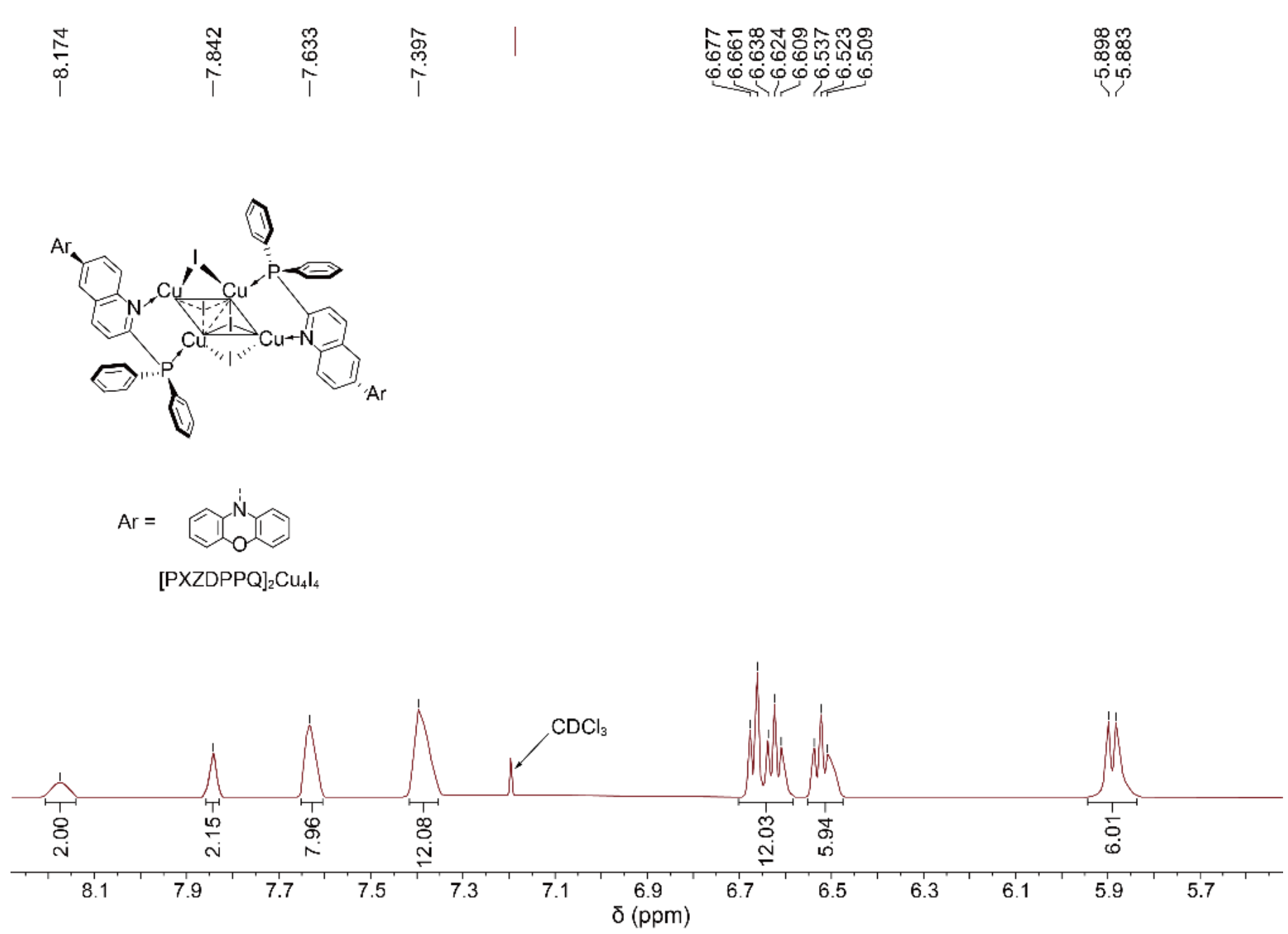

Supplementary Fig. 8 | ^{1}H NMR spectrum of $[PXZDPPQ]_2Cu_4I_4$ in $CDCl_3$.

Supplementary Note 2. Physical Measurements and Structures

^{1}H NMR spectra were recorded using a Varian Mercury plus 400NB spectrometer relative to tetramethylsilane (TMS) as internal standard. Molecular masses were determined by a FINNIGAN LCQ Electro-Spraying Ionization-Mass Spectrometry (ESI-MS), or a MALDI-TOF-MS. Elemental analyses were performed on a Vario EL III elemental analyzer. The crystals suitable for single-crystal XRD analysis were obtained through slowly diffusing ether into dichloromethane solution of the clusters at room temperature. All diffraction data were collected at 295 K on a Rigaku Xcalibur E diffractometer with graphite monochromatized Mo Kα (λ = 0.71073 Å) radiation in ω scan mode. All structures were solved by direct method and difference Fourier syntheses. Non-hydrogen atoms were refined by full-matrix least-squares techniques on F2 with anisotropic thermal parameters. The hydrogen atoms attached to carbons were placed in calculated positions with C−H = 0.93 Å and U(H) = 1.2Ueq(C) in the riding model approximation. All calculations were carried out with the SHELXL97 program. Cyclic voltammetric (CV) studies were conducted using an Eco Chemie B. V. AUTOLAB potentiostat in a typical three-electrode cell with a glassy carbon working electrode, a platinum wire counter electrode, and a silver/silver chloride (Ag/AgCl) reference electrode. Absorption spectra were measured using a SHIMADZU UV-3150 spectrophotometer.

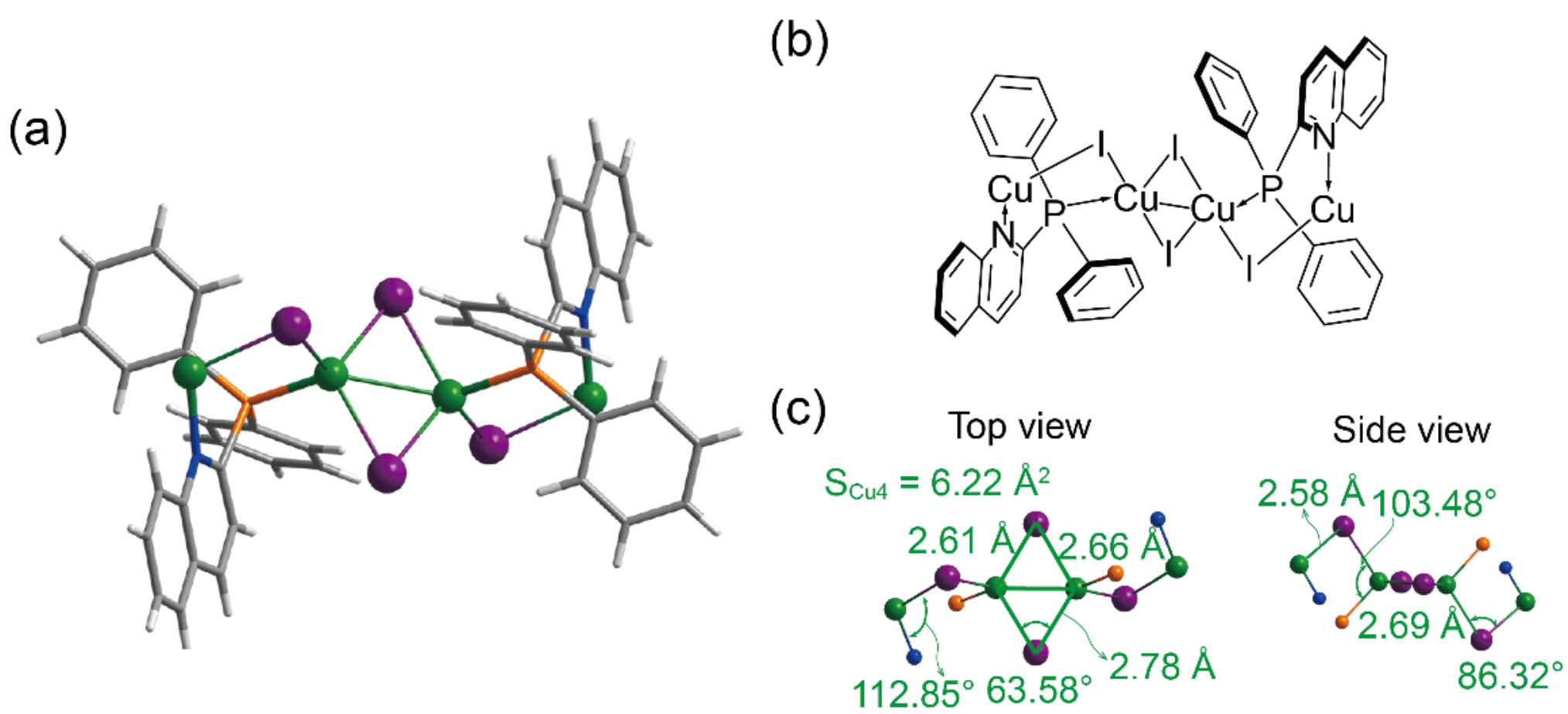

Supplementary Fig. 9 | Single-crystal X-ray diffraction results of $[DPPQ]_2Cu_4I_4$. (a) Molecular and (b) chemical structures of $[DPPQ]_2Cu_4I_4$. (c) The bond lengths and the bond angles of Cu_4I_4 in coordination skeletons are highlighted from top and side views.

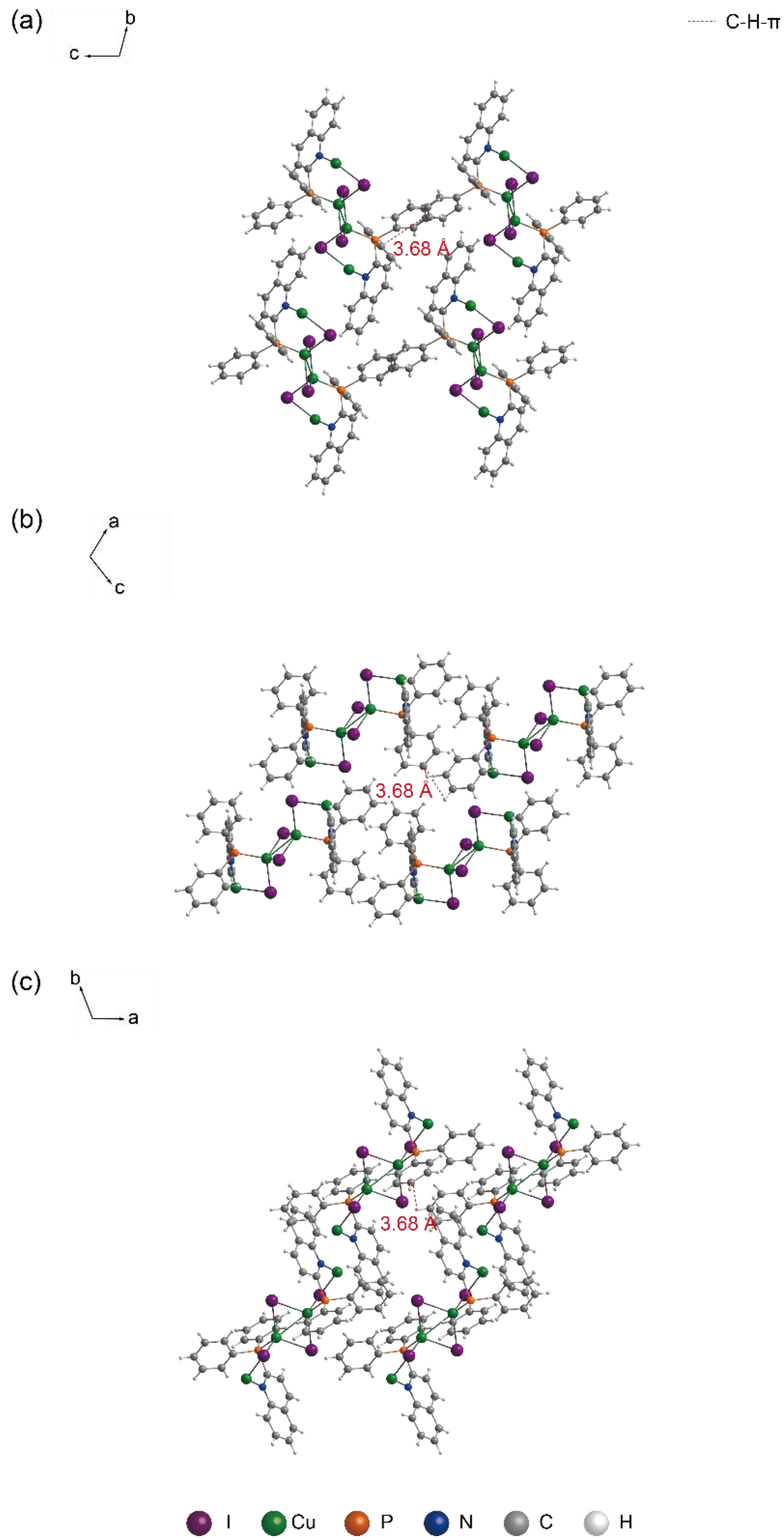

Supplementary Fig. 10 | Single crystal packing diagrams of $[DPPQ]_2Cu_4I_4$ viewed along *a*, *b* and *c* axes.

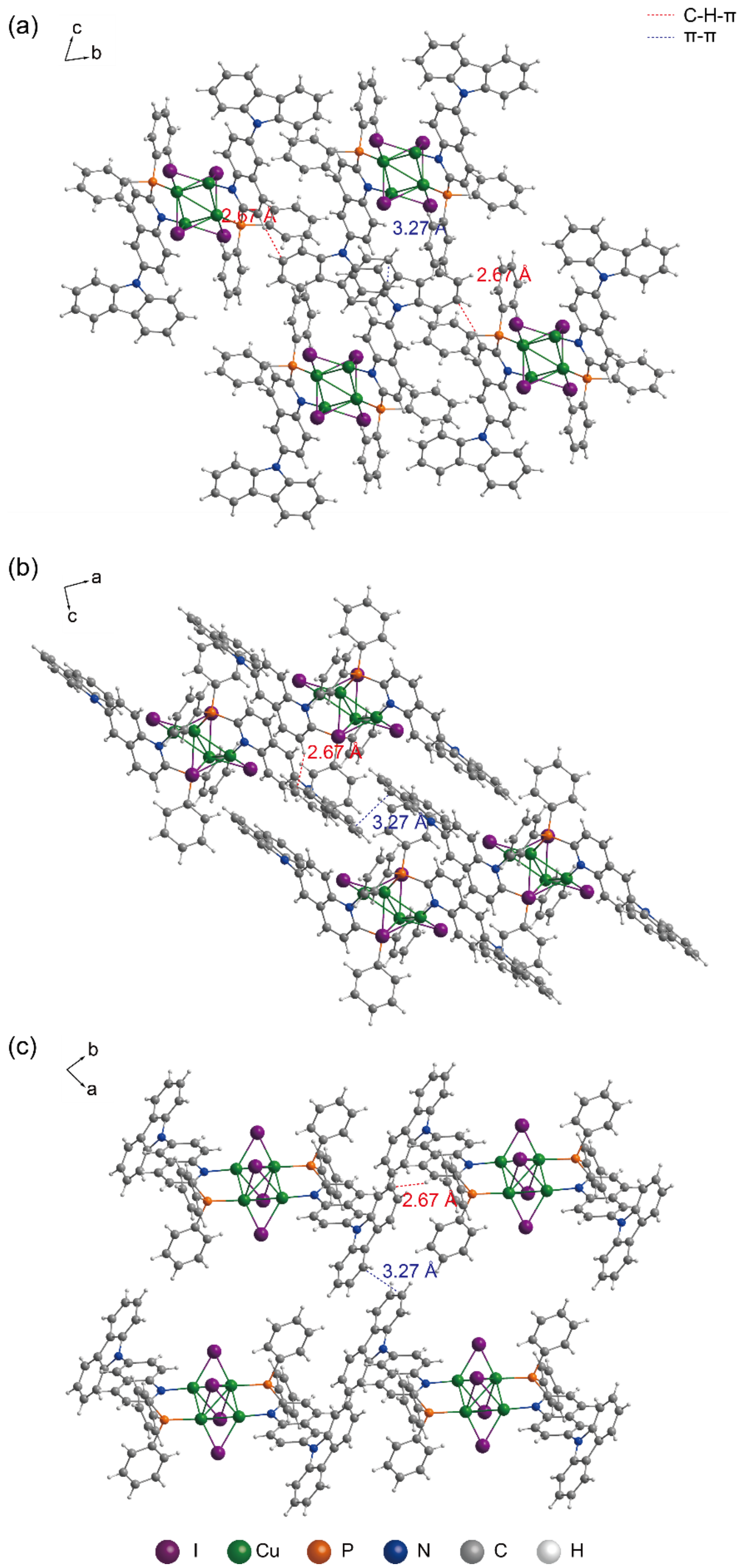

Supplementary Fig. 11 | Single crystal packing diagrams of $[CzDPPQ]_2Cu_4I_4$ viewed along *a*, *b* and *c* axes.

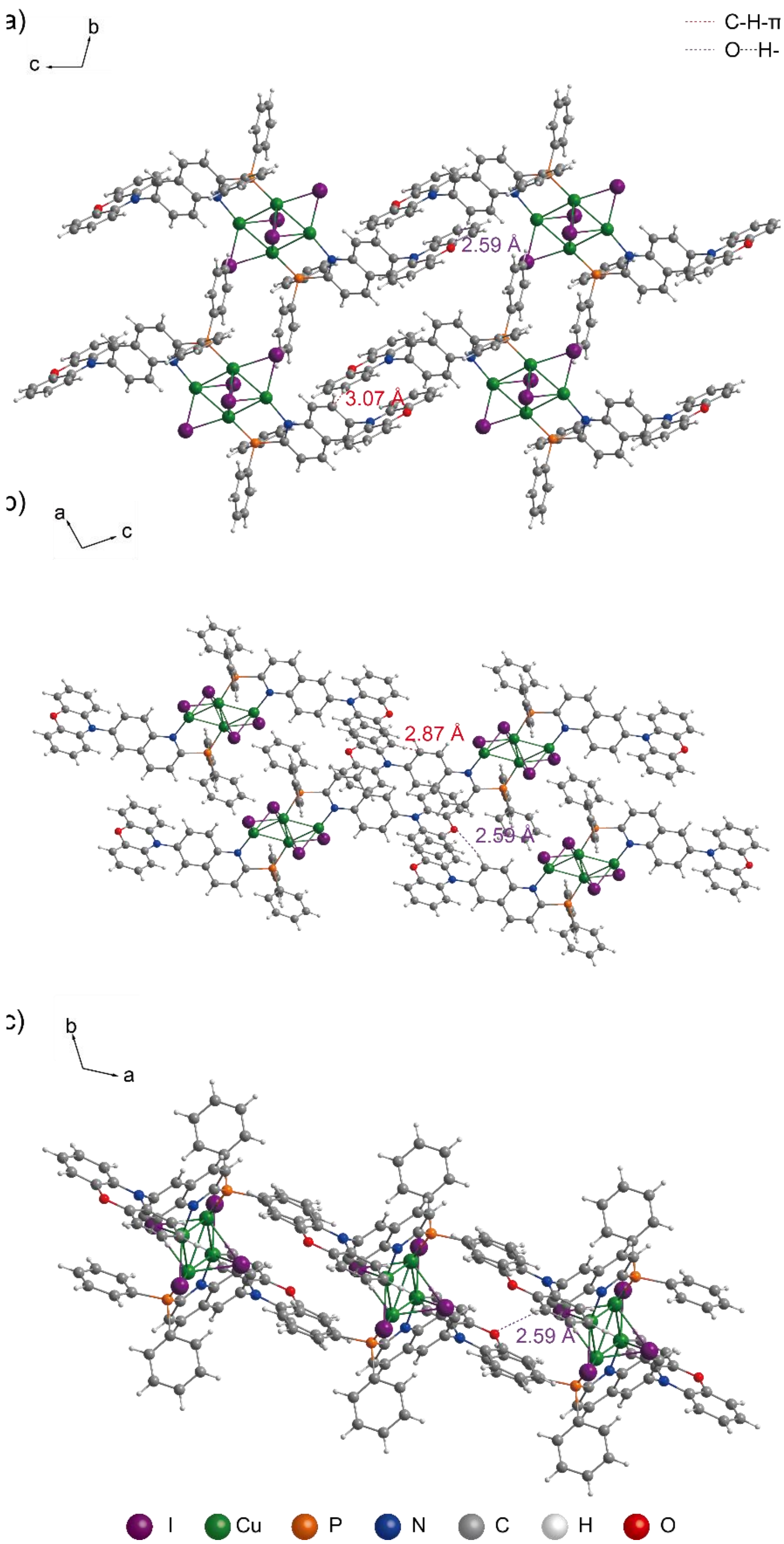

Supplementary Fig. 12 | Single crystal packing diagrams of $[PXZDPPQ]_2Cu_4I_4$ viewed along *a*, *b* and *c* axes. The single-crystal packing diagrams show that the bulky and peripheral Cz and PXZ groups effectively hinder molecular packing of $[CzDPPQ]_2Cu_4I_4$

and $[PXZDPPQ]_2Cu_4I_4$, thereby alleviating the aggregation-induced quenching (**Supplementary Figs 4** and **5**). Nonetheless, the limited dihedral angles between Cz and quinoline still render π-π stacking for $[CzDPPQ]_2Cu_4I_4$. On the contrary, the markedly distorted structure of PXZDPPQ almost eliminates π-π interactions, but provides O···H-C intermolecular hydrogen bonds for establishing inter-ligand charge transfer.

Supplementary Table 1 | The selected bond lengths and angles of $[DPPQ]_2Cu_4I_4$, $[CzDPPQ]_2Cu_4I_4$ and $[PXZDPPQ]_2Cu_4I_4$.

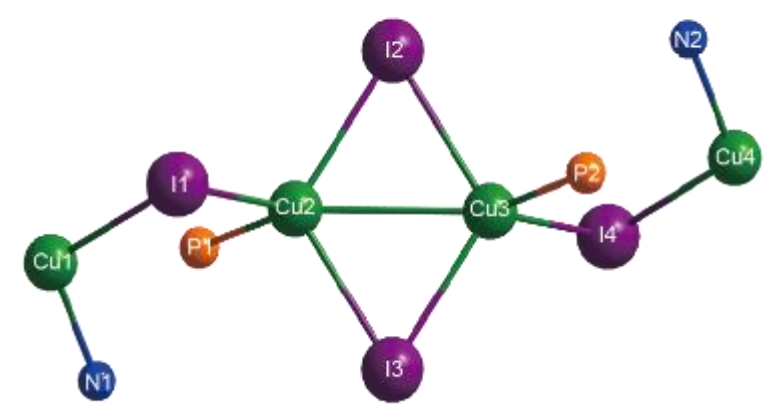

$[DPPQ]_2Cu_4I_4$

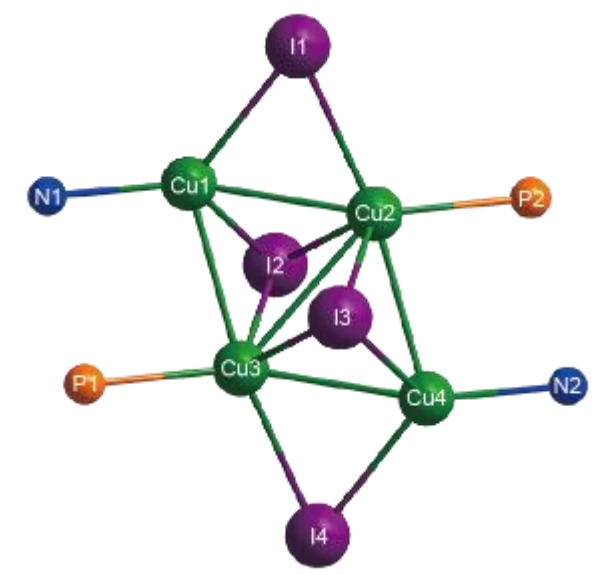

$[CzDPPQ]_2Cu_4I_4$

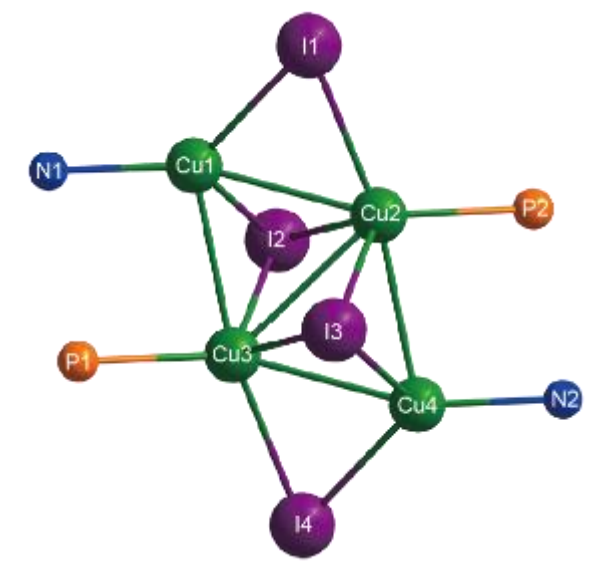

$[PXZDPPQ]_2Cu_4I_4$

Cluster	Bond length (Å)				Angle (°)			
	Cu1 - N1	2.005	Cu3 - I2	2.661	N1 - Cu1 - I1	112.90	P1 - Cu2 - I3	106.61
	Cu1 - I1	2.578	Cu3 - I3	2.612	Cu1 - I1 - Cu2	86.31	P1 - Cu2 - Cu3	129.97
	Cu2 - I1	2.687	Cu3 - P2	2.246	I1 - Cu2 - P1	103.49	I2 - Cu2 - I3	116.38
$[DPPQ]_2Cu_4I_4$	Cu1 - P1	2.246	Cu3 - I4	2.687	I1 - Cu2 - I2	110.91	I2 - Cu2 - Cu3	59.05
	Cu2 - Cu3	2.779	Cu4 - I4	2.578	I1 - Cu2 - I3	105.29	Cu2 - I2 - Cu3	63.62
	Cu2 - I2	2.612	Cu4 - N2	2.005	I1 - Cu2 - Cu3	126.00	I2 - Cu3 - Cu2	57.33
	Cu2 - I3	2.661			P1 - Cu2 - I2	113.10		
	Cu1 - N1	1.985	Cu4 - I3	2.612	N1 - Cu1 - I1	124.88	Cu1 - Cu3 - Cu4	115.12
$[CzDPPQ]_2Cu_4I_4$	Cu1 - I1	2.582	Cu3 - Cu4	2.606	N1 - Cu1 - Cu3	99.25	Cu1 - Cu2 - I2	57.56
	Cu1 - Cu2	2.606	Cu3 - I4	2.631	I1 - Cu1 - Cu2	60.94	I2 - Cu1 - Cu3	60.85
	Cu1 - Cu3	2.676	Cu4 - I4	2.582	I1 - Cu1 - Cu3	122.67	Cu1 - I2 - Cu3	60.75

	Cu1 - I2	2.612	Cu2 - Cu4	2.676	Cu2 - Cu1 - I2	65.10	I2 - Cu3 - Cu1	58.40
	Cu2 - I1	2.631	Cu2 - P2	2.252	Cu2 - Cu1 - Cu3	64.88	I2 - Cu2 - Cu3	56.69
	Cu2 - I2	2.808	Cu4 - N2	1.985	Cu1 - I1 - Cu2	59.99	I2 - Cu3 - Cu2	61.16
	Cu3 - P1	2.252			I1 - Cu2 - Cu1	59.07	Cu2 - I2 - Cu3	62.15
	Cu2 - I3	2.679			I1 - Cu2 - P2	110.96	Cu1 - I2 - Cu2	57.35
	Cu2 - Cu3	2.834			I1 - Cu2 - Cu4	161.87	P2 - Cu2 - Cu4	81.97
	Cu3 - I2	2.679			Cu1 - Cu2 - Cu3	58.76	P2−Cu2−Cu4−N2[a]	6.5
	Cu3 - I3	2.808			Cu1 - Cu3 - Cu2	56.37		
	Cu1 - N1	2.006	Cu4 - I3	2.574	N1 - Cu1 - I1	125.46	Cu1 - Cu3 - Cu4	116.08
	Cu1 - I1	2.561	Cu3 - Cu4	2.643	N1 - Cu1 - Cu3	99.34	Cu1 - Cu2 - I2	57.80
	Cu1 - Cu2	2.643	Cu3 - I4	2.642	I1 - Cu1 - Cu2	61.00	I2 - Cu1 - Cu3	60.40
	Cu1 - Cu3	2.684	Cu4 - I4	2.561	I1 - Cu1 - Cu3	121.42	Cu1 - I2 - Cu3	61.86
	Cu1 - I2	2.574	Cu2 - Cu4	2.684	Cu2 - Cu1 - I2	66.07	I2 - Cu3 - Cu1	57.74
$[PXZDPPQ]_2Cu_4I_4$	Cu2 - I1	2.642	Cu2 - P2	2.238	Cu2 - Cu1 - Cu3	63.92	I2 - Cu2 - Cu3	55.70
	Cu2 - I2	2.845	Cu4 - N2	2.006	Cu1 - I1 - Cu2	61.04	I2 - Cu3 - Cu2	62.62
	Cu3 - P1	2.238			I1 - Cu2 - Cu1	57.96	Cu2 - I2 - Cu3	61.68
	Cu2 - I3	2.647			I1 - Cu2 - P2	113.59	Cu1 - I2 - Cu2	58.13
	Cu2 - Cu3	2.821			I1 - Cu2 - Cu4	160.92	P2 - Cu2 - Cu4	81.14
	Cu3 - I2	2.647			Cu1 - Cu2 - Cu3	58.75	P2−Cu2−Cu4−N2[a]	12.5
	Cu3 - I3	2.845			Cu1 - Cu3 - Cu2	57.33		

[a] Torsion angle of coordination geometry.

Supplementary Note 3. Theoretical Simulation

Density functional theory (DFT) and time-dependent DFT (TDDFT) computations were carried out with different parameters for structure optimizations and vibration analyses. The ground state (S_0) configuration was established according to single crystal data. The S_0, singlet and triplet states in vacuum were simulated by the restricted and unrestricted formalism of Beck's three-parameter hybrid exchange functional[1] and Lee, and Yang and Parr correlation functional[2] B3LYP/6-31G(d,p) for ligands and double-ζ LANL2DZ basis sets for CuI, respectively. The fully optimized stationary points were further characterized by harmonic vibrational frequency analysis to ensure that real local minima had been found without imaginary vibrational frequency. The total energies were also corrected by zero-point energy both for the ground state and triplet state. Natural transition orbital (NTO) analysis was performed on the basis of optimized ground-state geometries at the same level.[3] The contours were visualized with Gaussview 5.0. All computations were performed using the Gaussian 09 package.

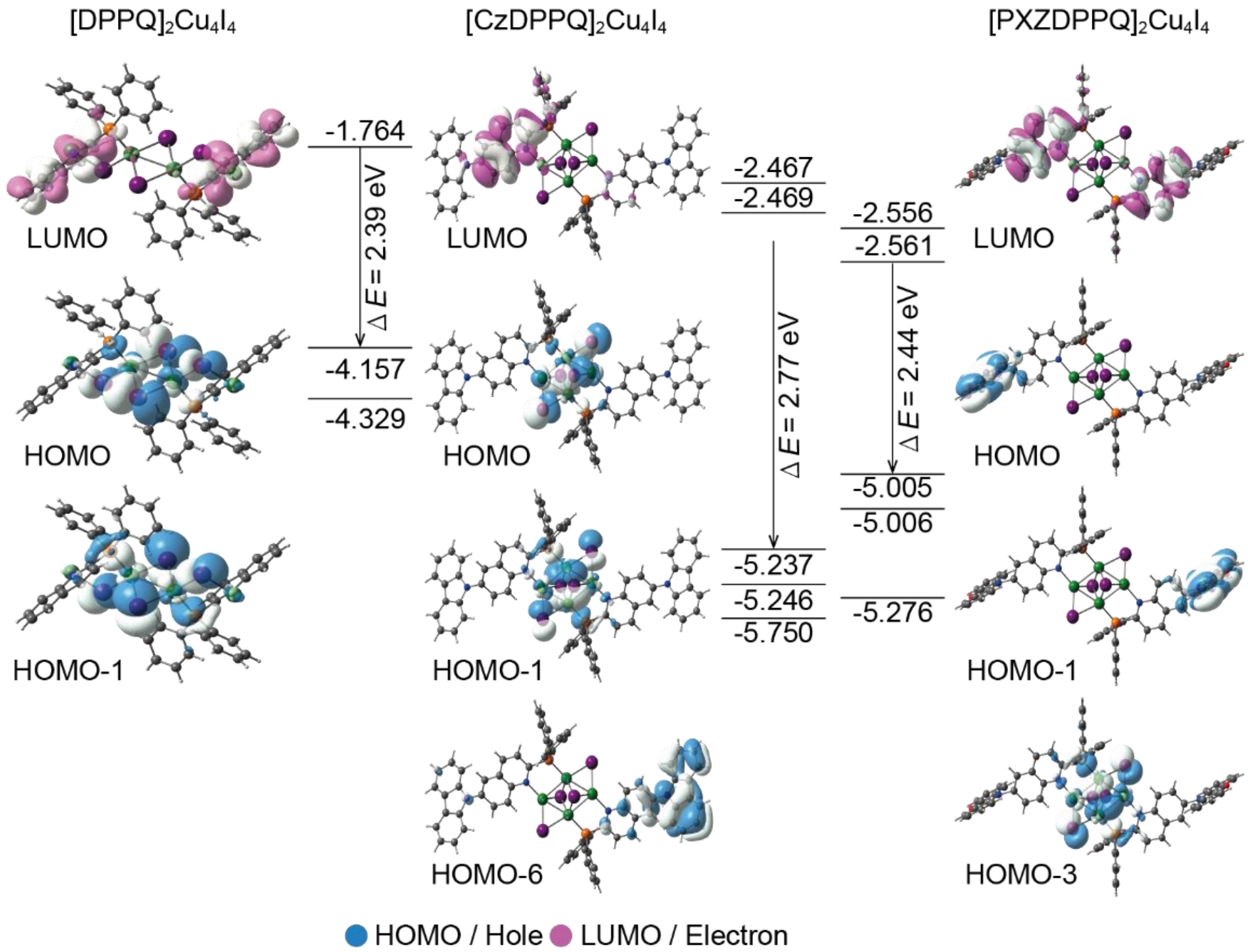

Supplementary Fig. 13 | FMO energy levels and contours of $[DPPQ]_2Cu_4I_4$ $[CzDPPQ]_2Cu_4I_4$ and $[PXZDPPQ]_2Cu_4I_4$ simulated at the level of B3LYP/6-31G*. Energy gap between HOMO and HOMO-5 of $[CzDPPQ]_2Cu_4I_4$ is within 0.4 eV.

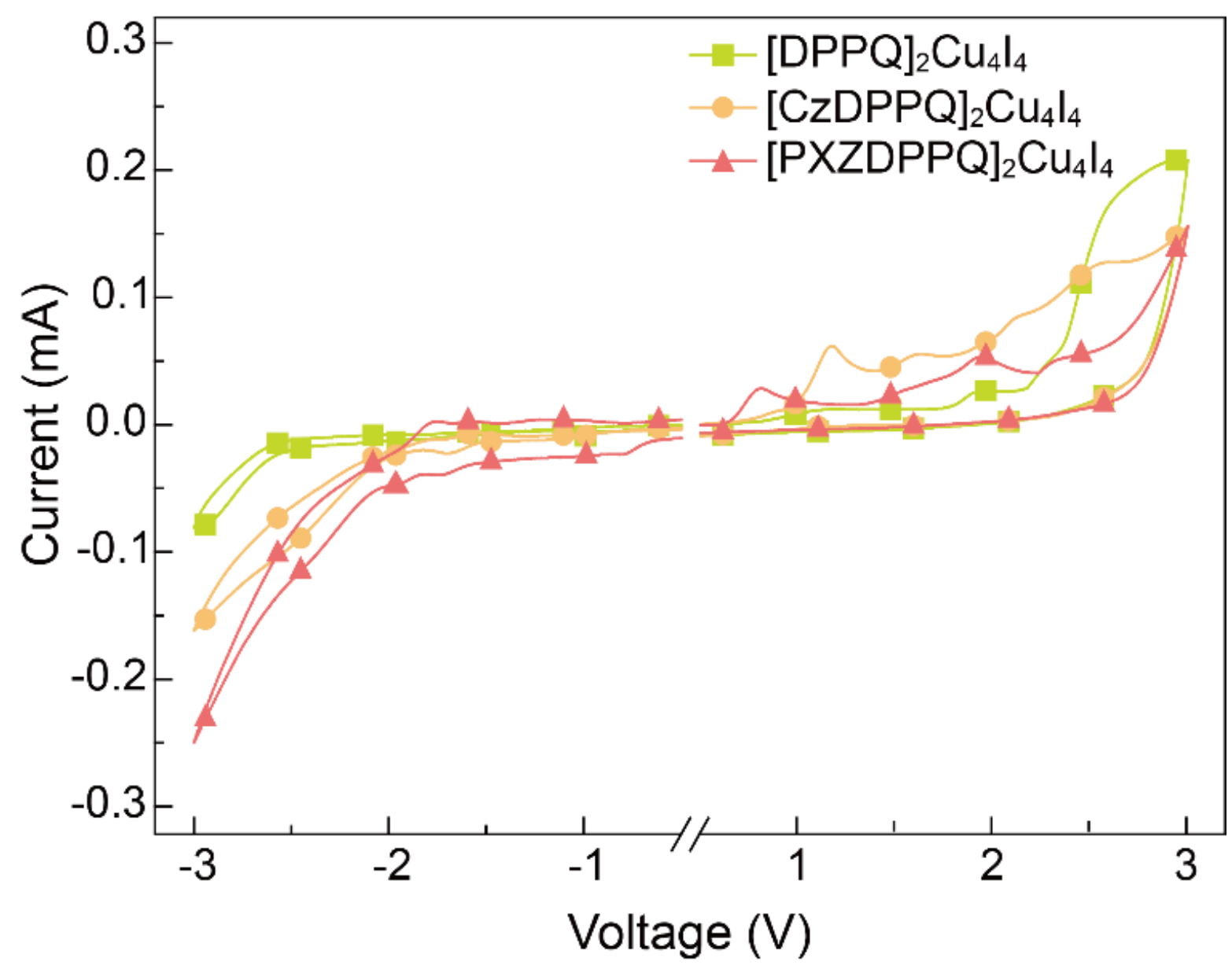

Supplementary Fig. 14 | CV curves of $[DPPQ]_2Cu_4I_4$, $[CzDPPQ]_2Cu_4I_4$ and $[PXZDPPQ]_2Cu_4I_4$ measured at room temperature with the scanning rate of 100 mV s^{-1}.

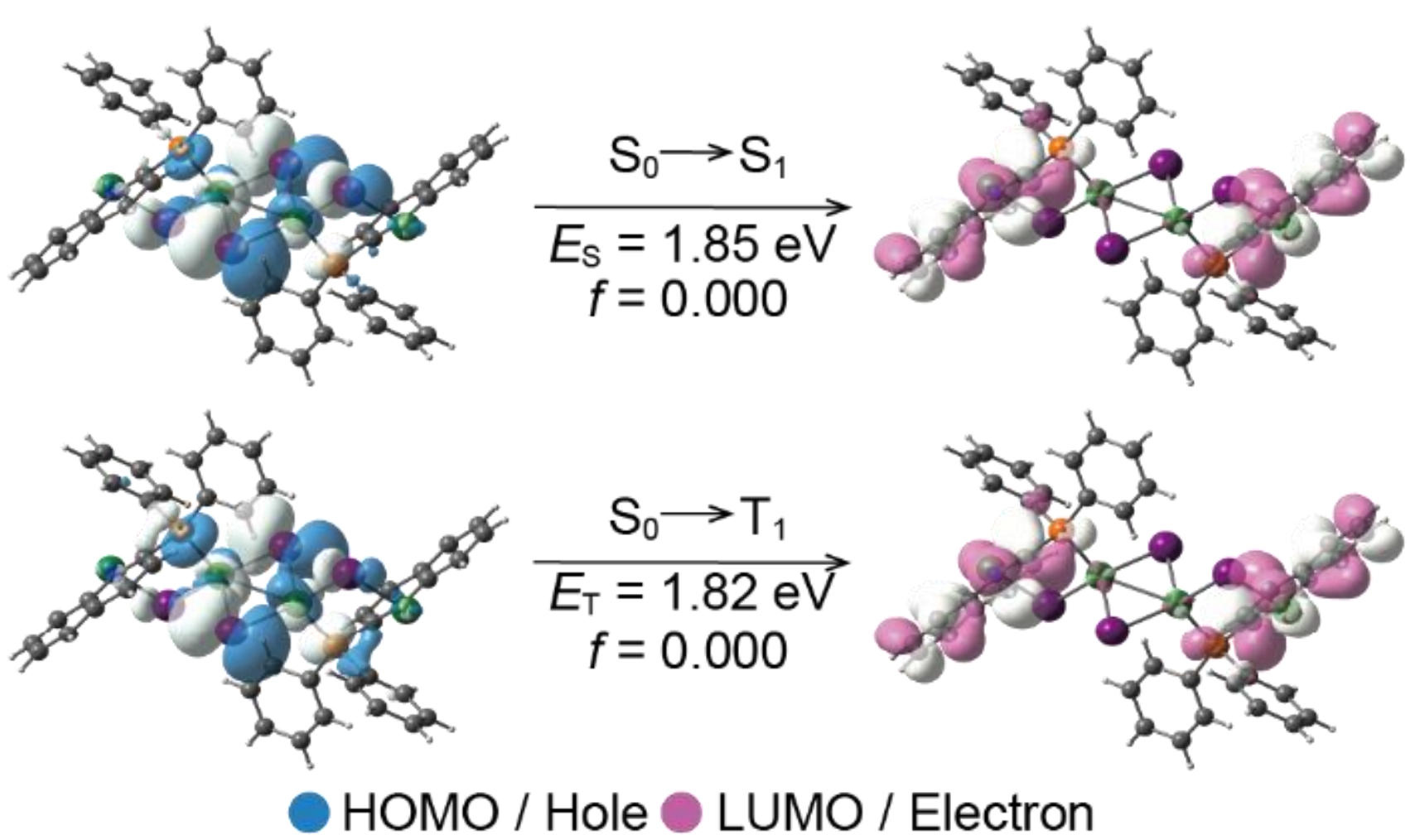

Supplementary Fig. 15 | Contours and key transition parameters of the $S_0 \rightarrow S_1$ and $S_0 \rightarrow T_1$ excitations for $[DPPQ]_2Cu_4I_4$ simulated with NTO analysis. "E_S" and "E_T" are the singlet and triplet energy levels. "f_S" is singlet oscillator strength.

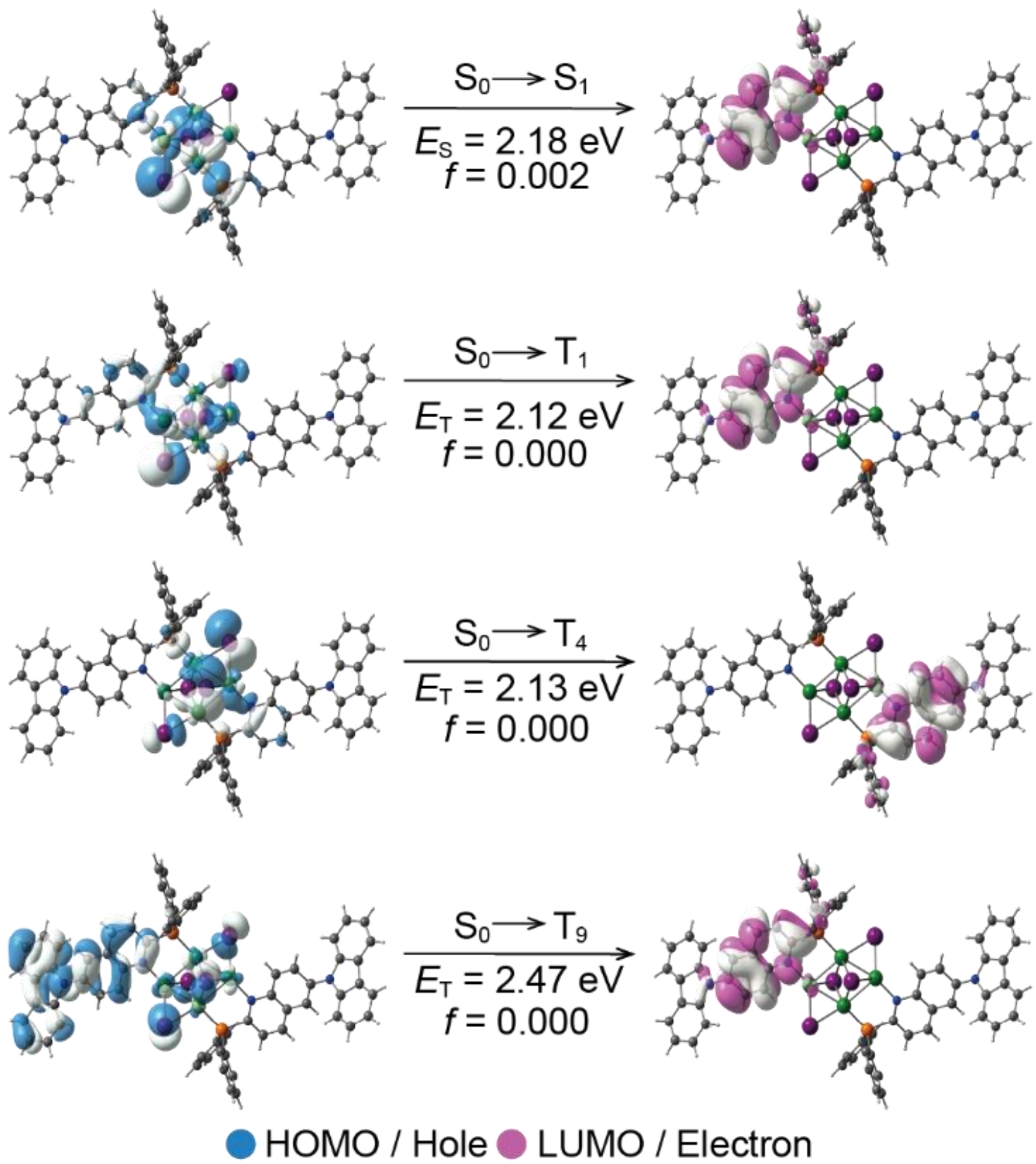

Supplementary Fig. 16 | Contours and key transition parameters of the $S_0 \rightarrow S_1$, $S_0 \rightarrow T_1$, $S_0 \rightarrow T_4$ and $S_0 \rightarrow T_9$ excitations for $[CzDPPQ]_2Cu_4I_4$ simulated with NTO analysis.

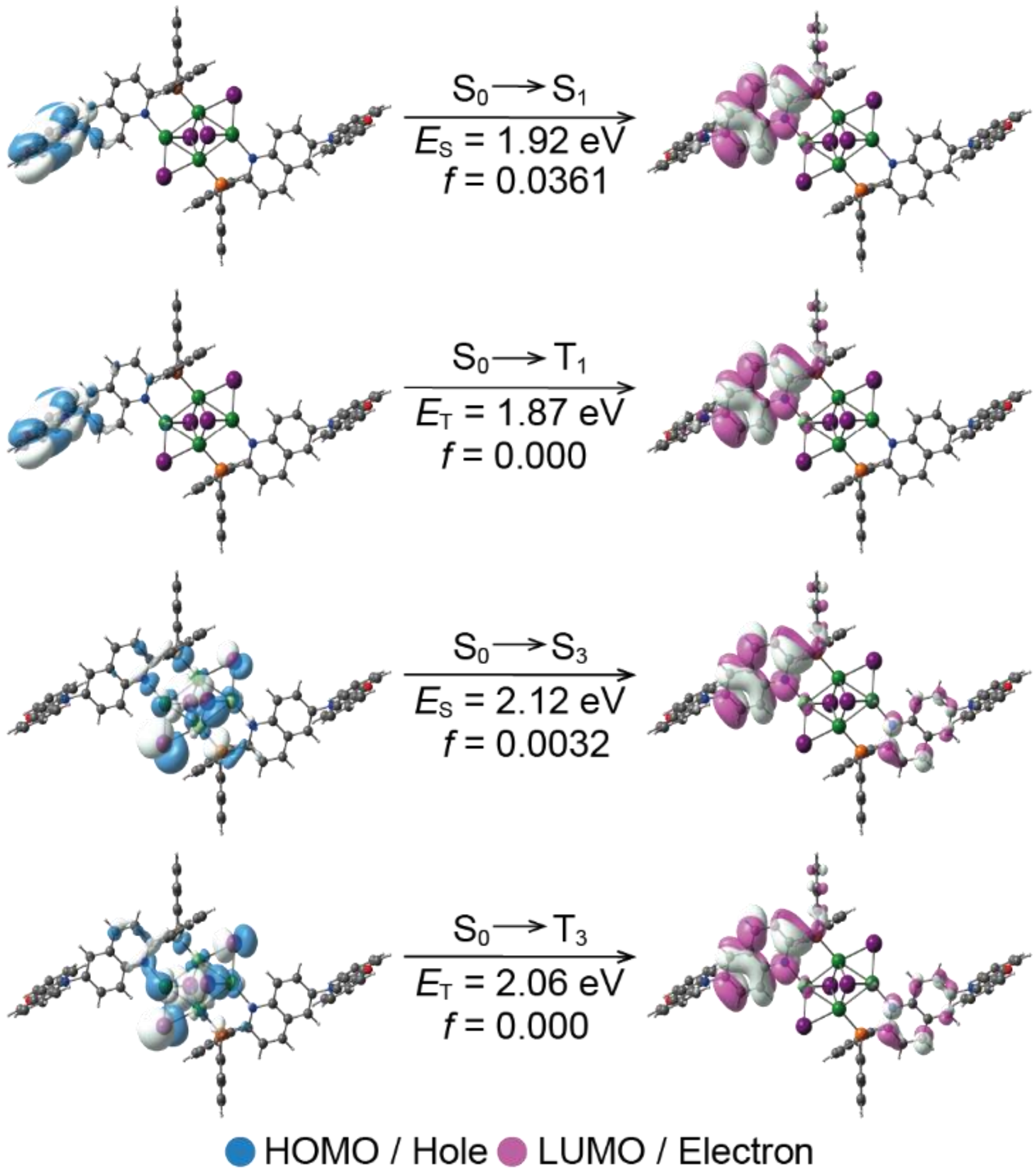

Supplementary Fig. 17 | Contours and key transition parameters of the $S_0 \rightarrow S_1$, $S_0 \rightarrow T_1$, $S_0 \rightarrow S_3$, and $S_0 \rightarrow T_3$ excitations for $[PXZDPPQ]_2Cu_4I_4$ simulated with NTO analysis.

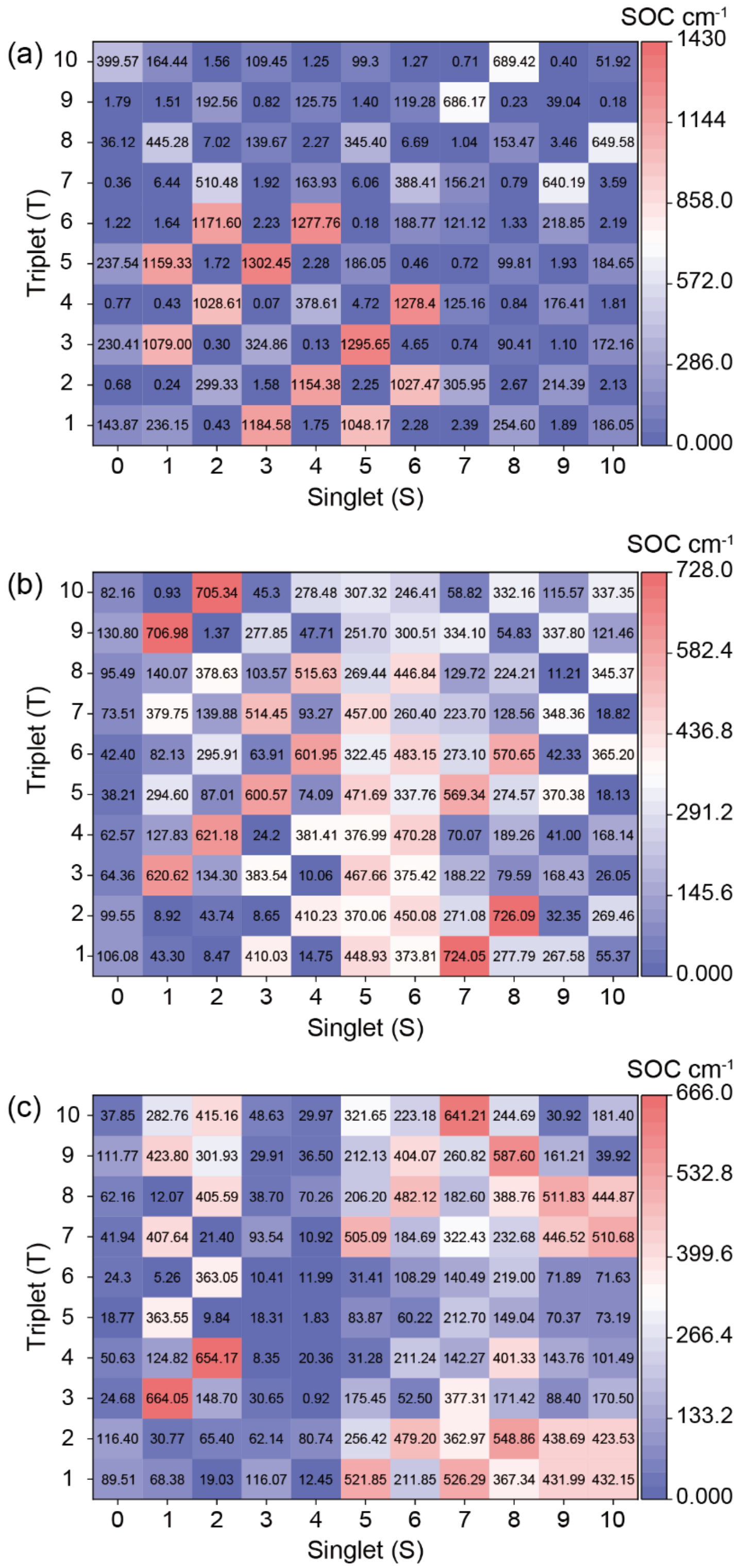

Supplementary Fig. 18 | Simulated spin-orbital coupling constants between triplet states T_m and singlet states S_n (m, n = 1-10) of (a) $[DPPQ]_2Cu_4I_4$, (b) $[CzDPPQ]_2Cu_4I_4$, and (c) $[PXZDPPQ]_2Cu_4I_4$. The color scale represents the SOC values in cm^{-1}.

Supplementary Note 4. Photophysical Analysis

1. Spectral measurement

Steady-state emission spectra were measured using an Edinburgh FPLS 1000 fluorescence spectrophotometer. Films were prepared by spin coating. Time-resolved emission spectra were measured with Time-Correlated Single Photon Counting (TCSPC) method with a nanosecond hyrogen flash lamp and a microsecond pulsed Xenon light source for 100 ps-10 s lifetime measurement, the synchronization photomultiplier for signal collection and the Multi-Channel Scaling Mode of the PCS900 fast counter PC plug-in card for data processing. A temperature controller was equipped to achieve 11-500 K variation. Spectra of prompt fluorescence (PF), delayed fluorescence (DF) and phosphorescence (PH) were sliced from time-resolved emission spectra (TRES) in the time ranges of <1 μs, 1-100 μs and >150 μs, respectively, by considering the predominance of PF, DF and PH components in the corresponding lifetime ranges.

2. Photoluminescence quantum yield measurement

Photoluminescence quantum yields (PLQY, η_{PL}) of these films were measured through a Labsphere 1-M-2 (ϕ = 6") integrating sphere coated with Benflect having efficient light reflection from 200-1600 nm, which was integrated with FPLS 1000. The absolute ϕ_{PL} determination of the sample was performed with two spectral (emission) scans, with the emission monochromator scanning over the Rayleigh scattered light from the sample and a blank substrate. The first spectrum recorded the scattered light and the sample emission, and the second spectrum recorded the scattered light of the Benflect coating. Integration and subtraction of the scattered light in the two spectra are equal to the number of photons absorbed by the samples (N_a), while integration of the sample emission is equal to the number of photons emitted (N_e). Then, absolute ϕ_{PL} can be estimated according to the equation of $\eta_{PL} = N_e/N_a$. Spectral correction (emission arm) was applied to raw data after background subtraction, and from these spectrally corrected curves, the quantum yield was calculated using an F900 software wizard.

3. Transition Parameter Calculation

Thermally activated delayed fluorescence (TADF) and PH ratios were used to determine the fractions of TADF (η_{TADF}) and PH (η_{PH}) quantum efficiencies in total η_{PL}. The integrals

of time decay curves give the total photon numbers contributed by TADF and PH. After subtracting PH contributions, PF and DF proportions in the rest TADF component can be further estimated by the ratios of photon numbers for PF and DF, according to their different lifetime ranges.[4] Then, PF (η_{PF}) and DF (η_{DF}) quantum efficiencies can be roughly estimated. The calculation formulas for the rate constants of PF (k_{PF}), DF (k_{DF}), singlet radiation (k_r^S), triplet radiation (k_r^T), singlet (k_{nr}^S) and triplet nonradiation (k_{nr}^T), reverse intersystem crossing (k_{RISC}) and intersystem crossing (k_{ISC}), and corresponding quantum efficiencies (η) are expressed as following list:[5, 6]

$$k_{\mathrm{PF}} = \frac{\eta_{\mathrm{PF}}}{\tau_{\mathrm{PF}}} \quad \text{(Eq. S1)}$$

$$k_{\mathrm{DF}} = \frac{\eta_{\mathrm{DF}}}{\tau_{\mathrm{DF}}} \quad \text{(Eq. S2)}$$

$$\mathrm{k_r^S} = \eta_{\mathrm{PF}} \cdot \mathrm{k_{PF}} \quad \text{(Eq. S3)}$$

$$k_{\mathrm{r}}^{\mathrm{T}} = \frac{\eta_{\mathrm{PH}}}{\tau_{\mathrm{PH}}} \quad \text{(Eq. S4)}$$

$$k_{\mathrm{ISC}} = \frac{\eta_{\mathrm{DF}}}{\eta_{\mathrm{DF}}+\eta_{\mathrm{PF}}} \cdot k_{\mathrm{PF}} \quad \text{(Eq. S5)}$$

$$k_{\mathrm{RISC}} = \frac{k_{\mathrm{DF}}\, k_{\mathrm{PF}}}{k_{\mathrm{ISC}}} \frac{\eta_{\mathrm{DF}}}{\eta_{\mathrm{PF}}} \quad \text{(Eq. S6)}$$

$$k_{nr}^S = k_{\mathrm{PF}} - k_r^S - k_{\mathrm{ISC}} = k_{\mathrm{PF}} - k_r^S - \frac{\eta_{\mathrm{DF}}}{\eta_{\mathrm{DF}}+\eta_{\mathrm{PF}}} \cdot k_{\mathrm{PF}} \quad \text{(Eq. S7)}$$

$$\mathrm{k_{nr}^T} = \mathrm{k_{DF}} - \left(1 - \frac{\mathrm{k_{ISC}}}{\mathrm{k_{PF}}}\right) \cdot \mathrm{k_{RISC}} = \mathrm{k_{DF}} - \left(1 - \frac{\mathrm{k_{ISC}}}{\mathrm{k_{PF}}}\right) \cdot \frac{\mathrm{k_{DF}\, k_{PF}}}{\mathrm{k_{ISC}}} \frac{\eta_{\mathrm{DF}}}{\eta_{\mathrm{PF}}} \quad \text{(Eq. S8)}$$

$$\eta_{\mathrm{ISC}} = \frac{k_{\mathrm{ISC}}}{k_{\mathrm{PF}}} \quad \text{(Eq. S9)}$$

$$\eta_{\mathrm{RISC}} = \frac{k_{\mathrm{RISC}}}{k_{\mathrm{nr}}^{\mathrm{T}}+k_{\mathrm{RISC}}} \quad \text{(Eq. S10)}$$

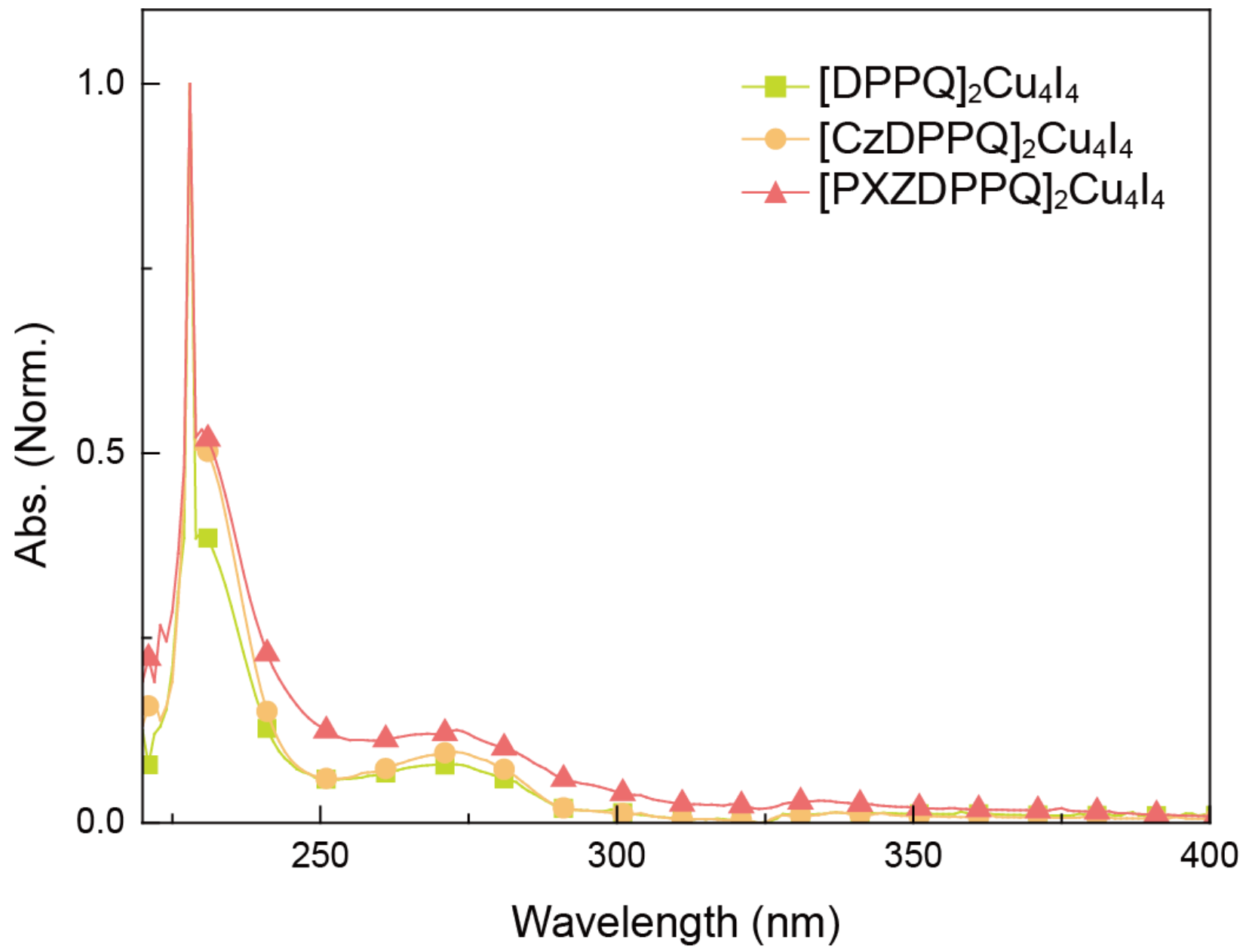

Supplementary Fig. 19 | Electronic absorption spectra of $[DPPQ]_2Cu_4I_4$, $[CzDPPQ]_2Cu_4I_4$ and $[PXZDPPQ]_2Cu_4I_4$ in dilute dichloromethane solution (10^{-6} mol·L^{-1}).

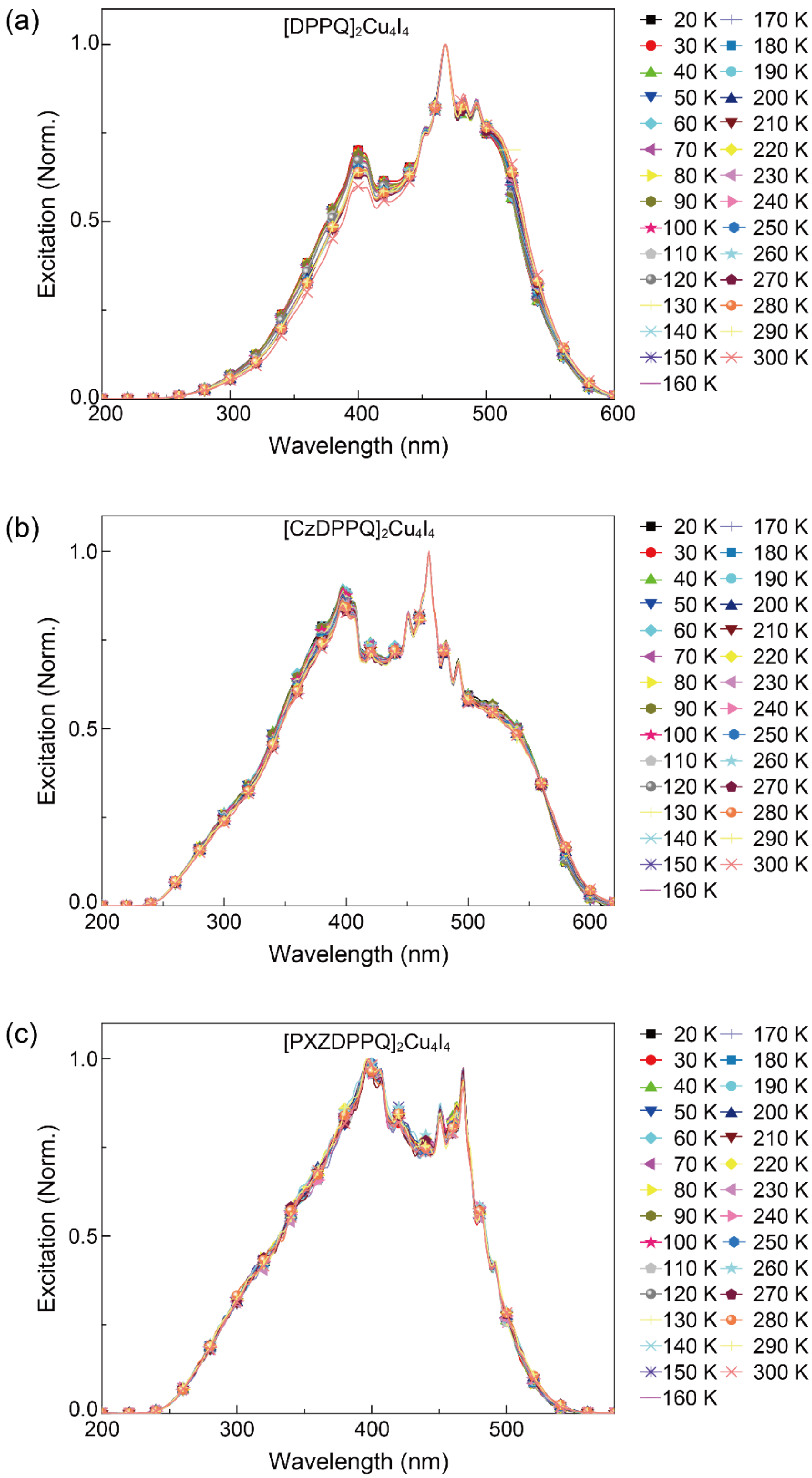

Supplementary Fig. 20 | Excitation spectra of neat films for (a) $[DPPQ]_2Cu_4I_4$, (b) $[CzDPPQ]_2Cu_4I_4$ and (c) $[PXZDPPQ]_2Cu_4I_4$.

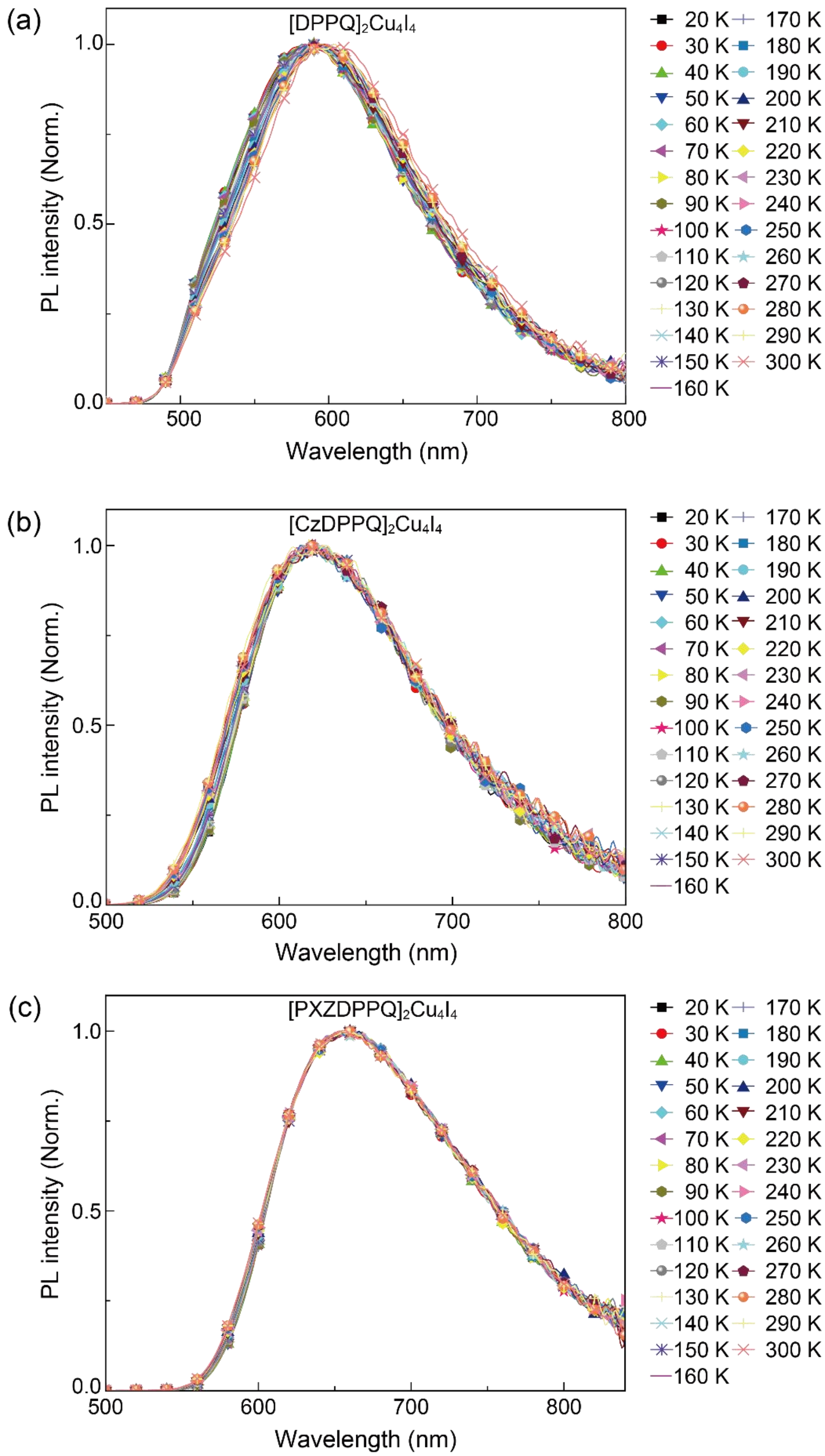

Supplementary Fig. 21 | PL spectra of neat films at different temperatures. (a) $[DPPQ]_2Cu_4I_4$. (b) $[CzDPPQ]_2Cu_4I_4$. (c) $[PXZDPPQ]_2Cu_4I_4$.

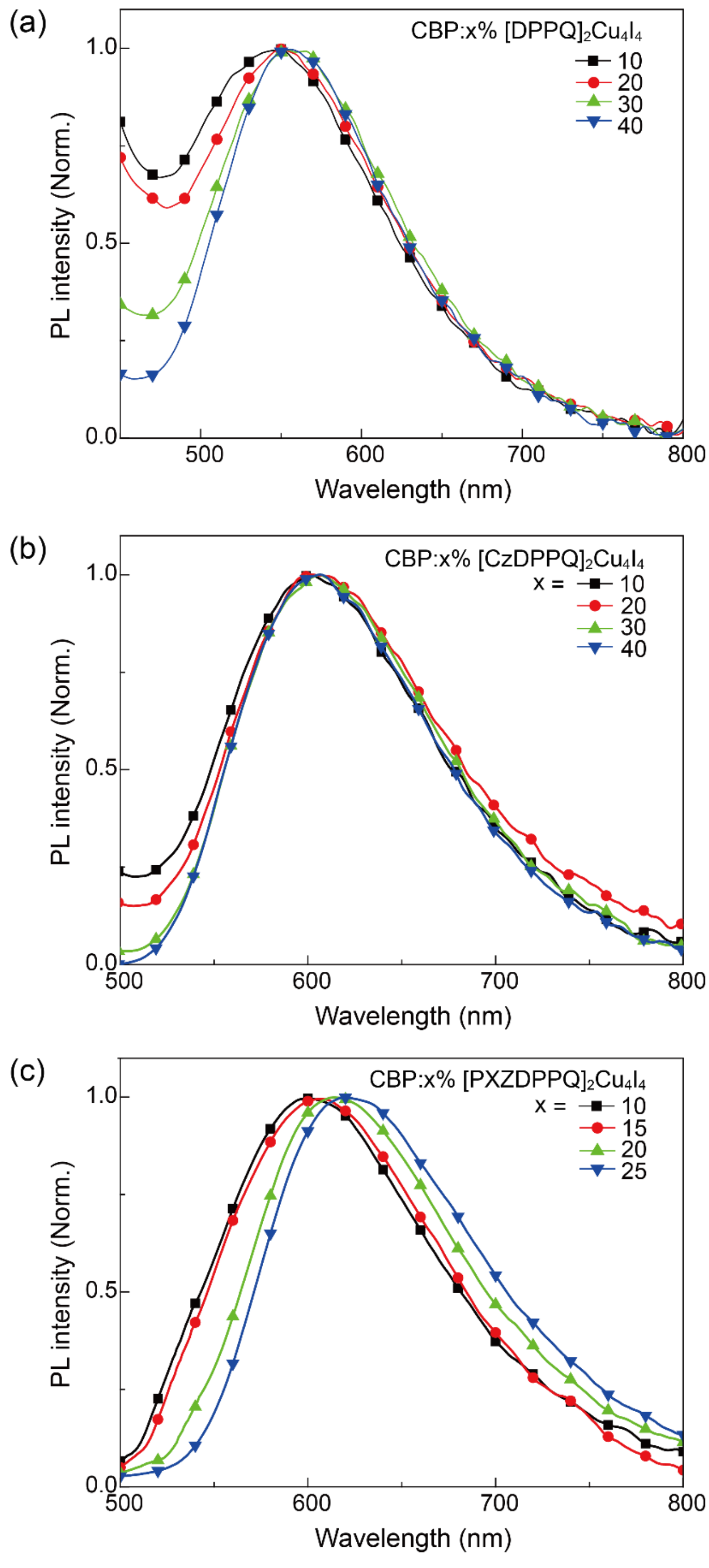

Supplementary Fig. 22 | Doping concentration dependence of PL spectra for CBP:*x*% cluster films based on (a) $[DPPQ]_2Cu_4I_4$ (x = 10, 20, 30 and 40), (b) $[CzDPPQ]_2Cu_4I_4$ (x = 10, 20, 30 and 40) and (c) $[PXZDPPQ]_2Cu_4I_4$ (x = 10, 15, 20 and 25).

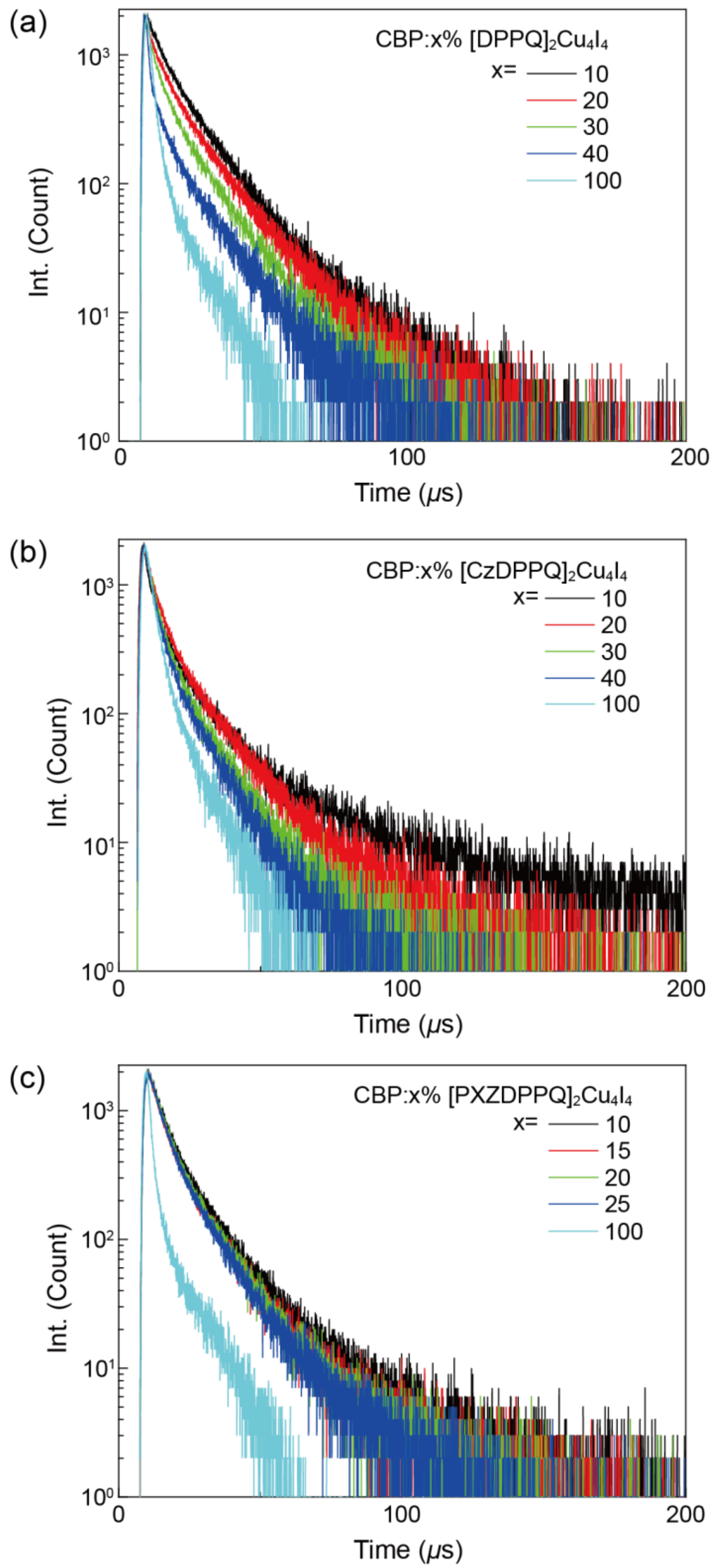

Supplementary Fig. 23 | Doping concentration dependence of time decays for CBP:*x*% cluster films based on (a) $[DPPQ]_2Cu_4I_4$ (x = 10, 20, 30, 40 and 100), (b) $[CzDPPQ]_2Cu_4I_4$ (x = 10, 20, 30, 40 and 100) and (c) $[PXZDPPQ]_2Cu_4I_4$ (x = 10, 15, 20, 25 and 100).

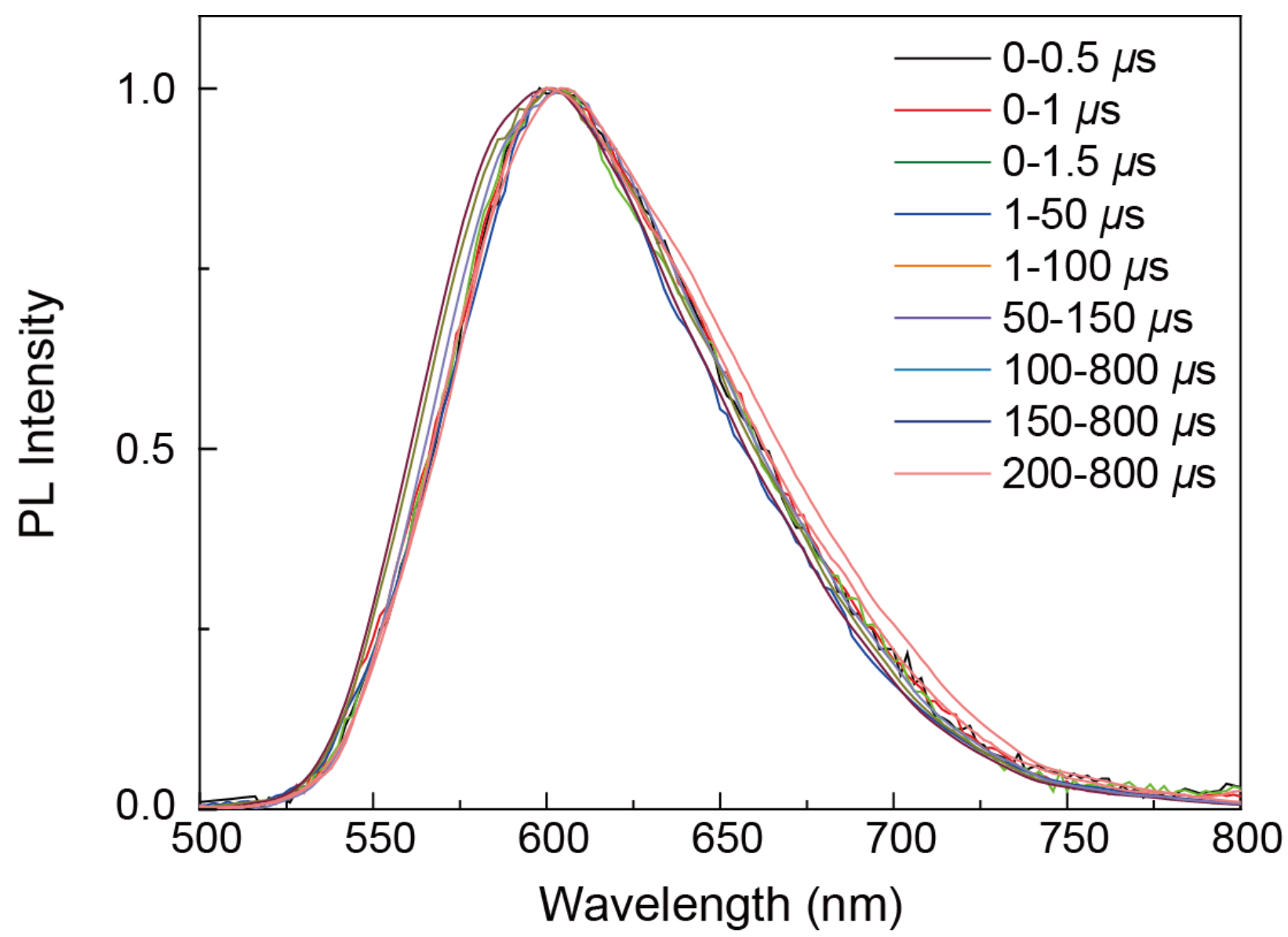

Supplementary Fig. 24 | PL spectra of CBP:15% $[PXZDPPQ]_2Cu_4I_4$ film at room temperature measured in the time ranges of 0-0.5, 0-1.0, 0-1.5, 1-50, 1-100, 50-150, 100-800, 150-800 and 200-800 μs, respectively.

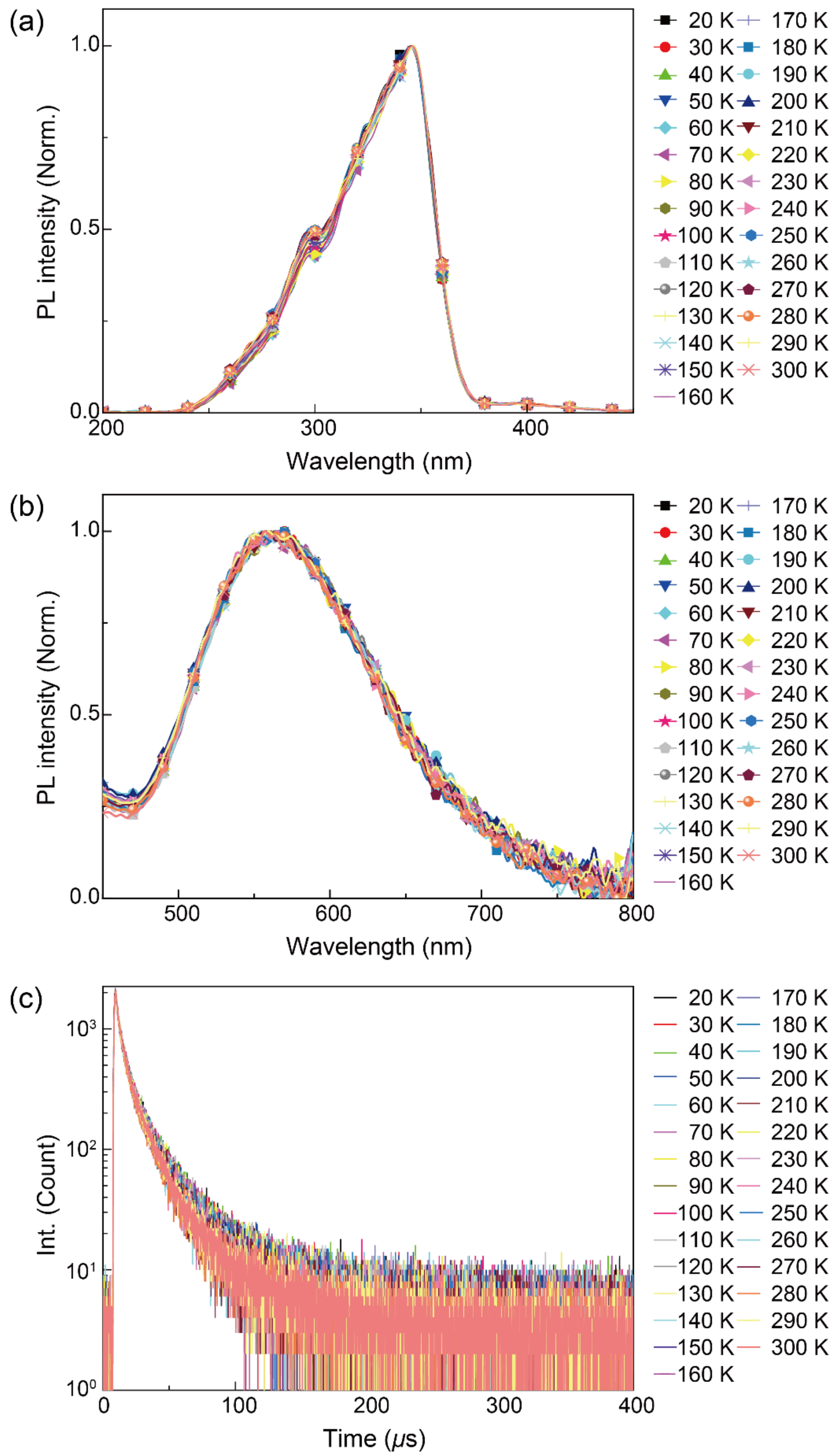

Supplementary Fig. 25 | Excitation, PL spectra and time decays of spin-coated CBP:30% $[DPPQ]_2Cu_4I_4$ film in the range of 20-300 K with an interval of 10 K. (a) Excitation spectra at emission wavelength of 556 nm. (b) Emission spectra. (c) Time decays at emission wavelength of 556 nm.

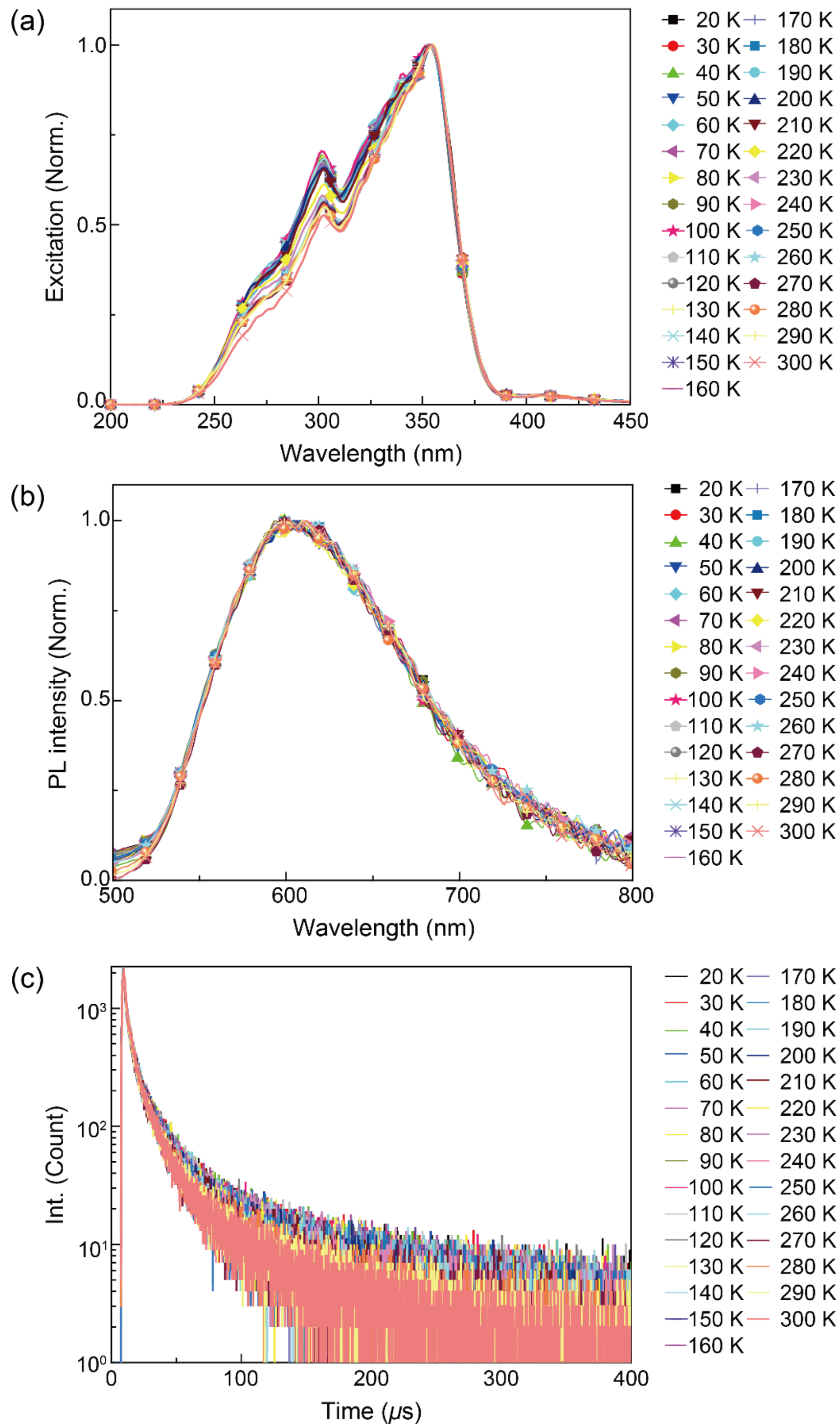

Supplementary Fig. 26 | Excitation and PL spectra and time decays of spin-coated CBP:30% $[CzDPPQ]_2Cu_4I_4$ film in the range of 20-300 K with an interval of 10 K. (a) Excitation spectra at emission wavelength of 603 nm. (b) Emission spectra. (c) Time decays at emission wavelength of 603 nm.

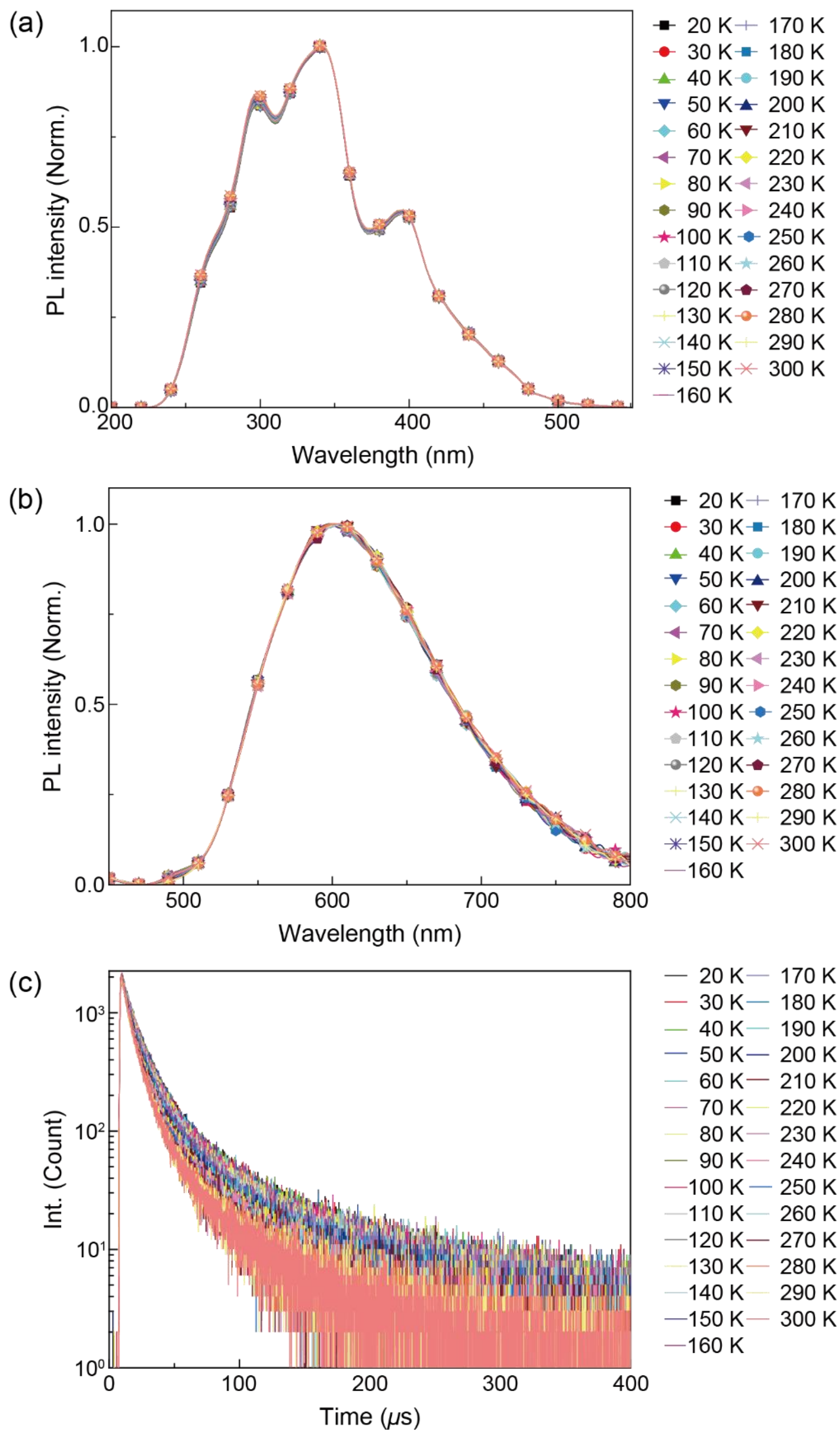

Supplementary Fig. 27 | Excitation and PL spectra and time decays of spin-coated CBP:15% $[PXZDPPQ]_2Cu_4I_4$ film in the range of 20-300 K with an interval of 10 K. (a) Excitation spectra at emission wavelength of 602 nm. (b) Emission spectra. (c) Time decays at emission wavelength of 602 nm.

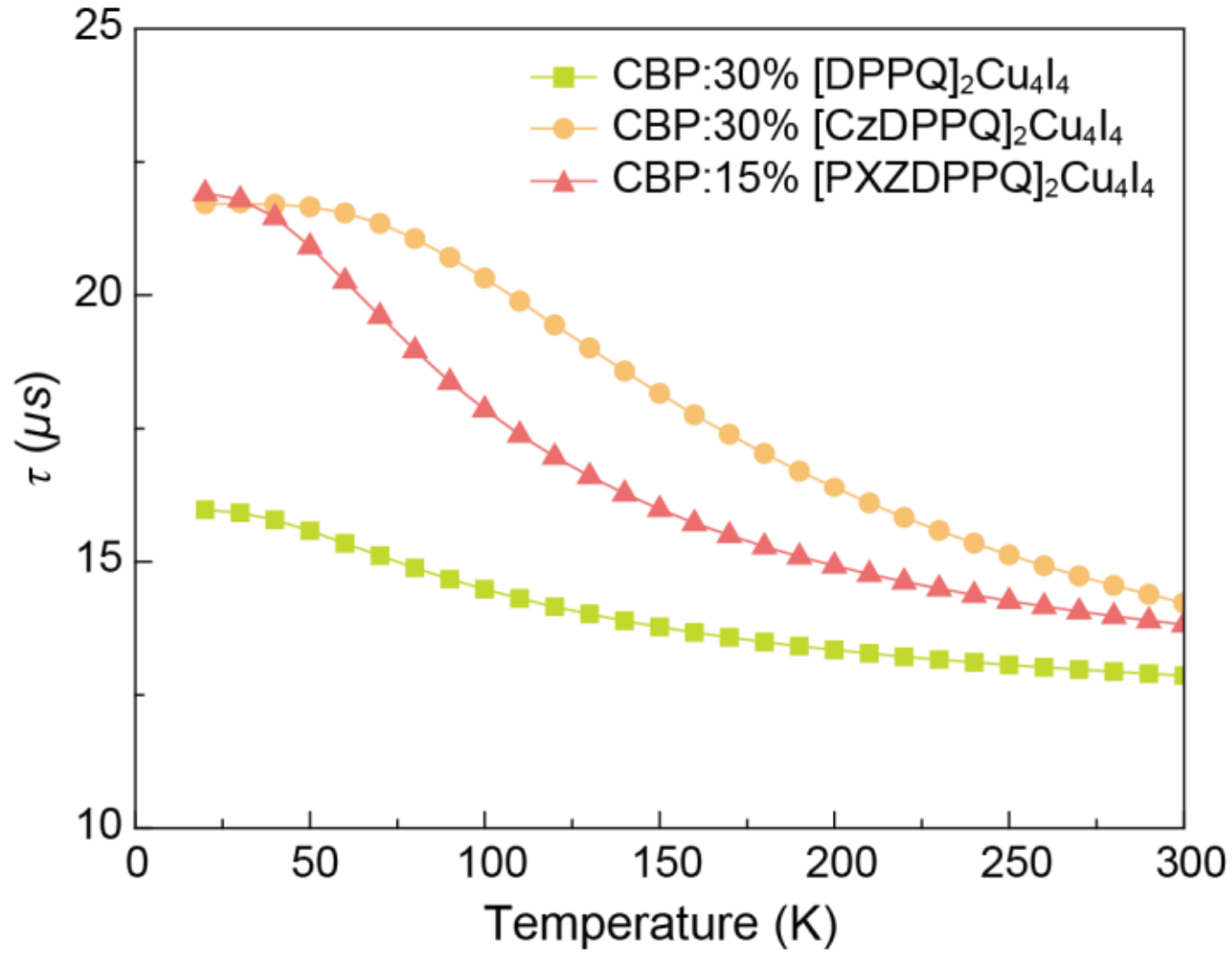

Supplementary Fig. 28 | Lifetime variations of CBP:30% $[DPPQ]_2Cu_4I_4$, CBP:30% $[CzDPPQ]_2Cu_4I_4$ and CBP:15% $[PXZDPPQ]_2Cu_4I_4$ in the temperature range of 20-300 K.

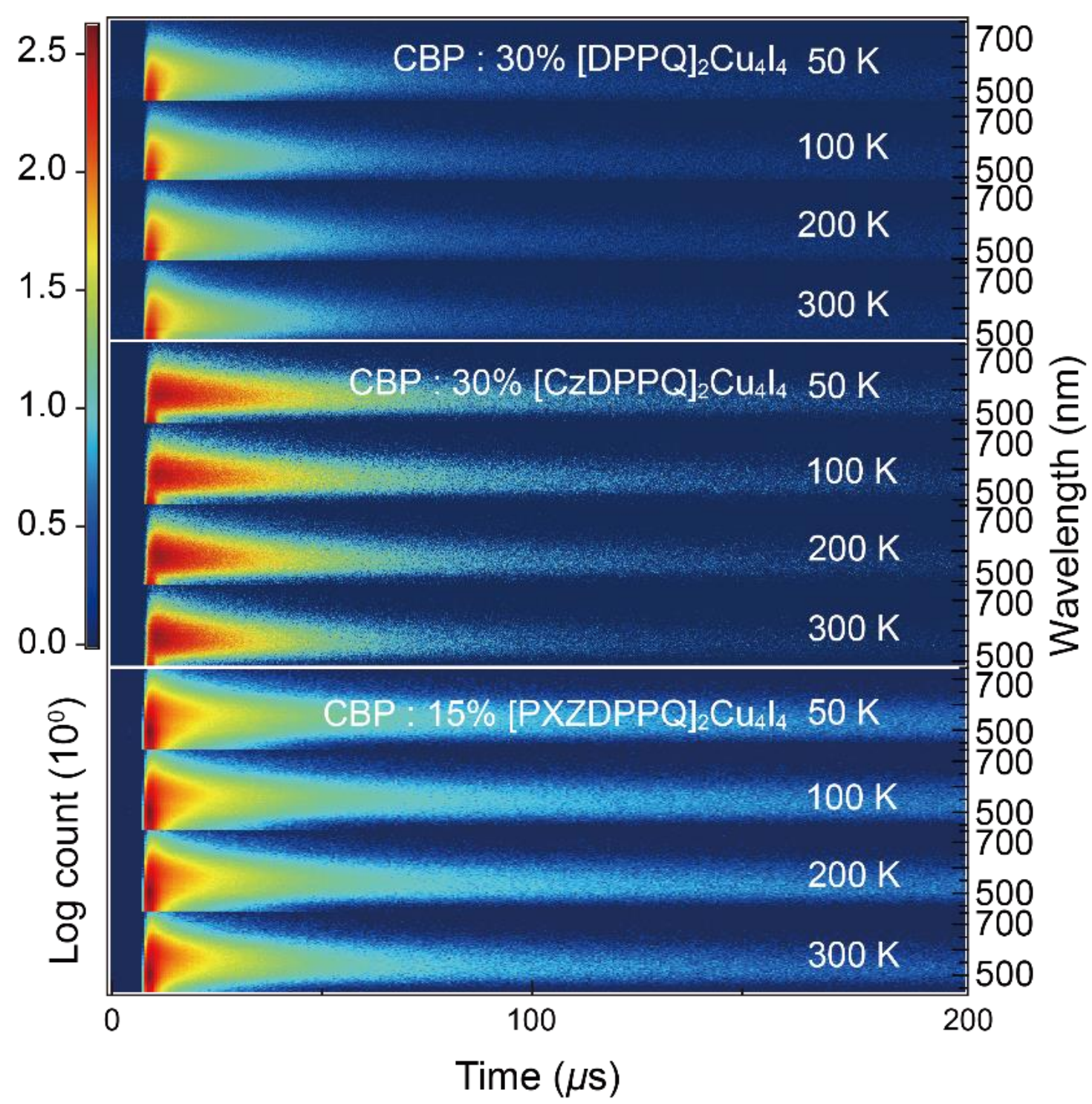

Supplementary Fig. 29 | Time-resolved emission spectra of neat films based on (a) CBP:30% $[DPPQ]_2Cu_4I_4$, (b) CBP:30% $[CzDPPQ]_2Cu_4I_4$ and (c) CBP:15% $[PXZDPPQ]_2Cu_4I_4$ at 50, 100, 200 and 300 K.

Supplementary Table 2 | Physical properties of the clusters.

Cluster	λ_{Abs} (nm)	λ_{PL}[b] (nm)	S_1 (eV)	T_1 (eV)	ΔE_{ST}[f] (eV)	τ[g] (μs)	PLQY (%)	HOMO (eV)	LUMO (eV)	FWHM[i] (nm)
$[DPPQ]_2Cu_4I_4$	276, 336[a]<br>281, 330, 381, 412, 431, 474[b]	597	2.38[c]<br>2.56[d]	2.31[e]<br>2.47[d]	0.07[c,e]<br>0.09[d]	5.55 (0.30),<br>12.49 (0.70)	5.36[b]	-4.16[d]<br>-5.56[h]	-1.76[d]<br>-3.24[h]	145
$[CzDPPQ]_2Cu_4I_4$	271, 333[a]<br>269, 327, 355, 393, 482[b]	610	2.23[c]<br>2.18[d]	2.19[e]<br>2.12[d]	0.04[c,e]<br>0.06[d]	5.22 (0.63),<br>12.08 (0.37)	26.37[b]	-5.24[d]<br>-5.52[h]	-2.47[d]<br>-3.30h]	131
$[PXZDPPQ]_2Cu_4I_4$	272, 338[a]<br>270,328, 346, 395[b]	653	2.14[c]<br>1.92[d]	2.09[e]<br>1.87[d]	0.05[c,e]<br>0.05[d]	5.16 (0.59),<br>11.23 (0.41)	27.41[b]	-5.01d]<br>-5.39[h]	-2.56[d]<br>-3.32[h]	157

[a] In dichloromethane solution (10^{-5} mol L^{-1}); [b] in neat film; [c] estimated according to absorption edge; [d] Gaussian simulation results of single molecules; [e] estimated according to peak wavelengths of time-resolved phosphorescence spectra after a delay of 100 μs; [f] singlet-triplet splitting; [g] lifetimes fitted according to time decays. Data in parentheses are percentages; [h] calculated according to onset voltages of cyclic voltammetric curves; [i] full width at half maximum.

Supplementary Table 3 | Key transition parameters of CBP:*x*% cluster films.

Cluster	*x*	η_{PL}[a] (%)	λ_{Em} (nm)	τ_{PF}[b] (ns)	τ_{DF}[c] (μs)	τ_{PH}[d] (μs)	η_{PF}[e] (%)	η_{DF}[f] (%)	η_{PH}[g] (%)	k_{PF}[h] (10^7 s^{-1})	k_{DF}[i] (10^4 s^{-1})	k_{ISC}[j] (10^7 s^{-1})	k_{RISC}[k] (10^4 s^{-1})	k_r^S[l] (10^7 s^{-1})	k_r^T[m] (10^4 s^{-1})	k_{nr}^S[n] (10^7 s^{-1})	k_{nr}^T[o] (10^4 s^{-1})	η_{ISC}[p] (%)	η_{RISC}[q] (%)
$[DPPQ]_2Cu_4I_4$	10	10.12	553	6.63	0.29	13.29	1.89	0.63	7.60	0.29	2.16	0.07	2.87	0.01	0.57	0.21	2.10	24.86	57.72
	20	11.21	555	5.65	0.33	12.89	2.14	0.74	8.34	0.38	2.23	0.10	3.00	0.01	0.65	0.27	2.17	25.64	58.06
	30	11.45	556	5.63	0.45	12.41	2.51	1.21	7.73	0.45	2.69	0.15	3.98	0.01	0.62	0.29	2.59	32.51	60.61
	40	9.98	558	4.56	0.31	12.47	1.95	0.71	7.32	0.43	2.28	0.11	3.11	0.01	0.59	0.31	2.22	26.61	58.33
$[CzDPPQ]_2Cu_4I_4$	10	80.7	597	9.43	3.69	14.19	18.25	9.64	52.80	1.94	2.61	0.67	3.99	0.35	3.72	0.91	1.88	34.57	67.95
	20	85.95	600	8.71	3.92	12.32	19.97	11.59	54.39	2.29	2.96	0.84	4.67	0.46	4.41	0.99	2.02	36.72	69.78
	30	91.61	603	8.60	5.57	9.21	22.52	17.33	51.76	2.62	3.11	1.14	5.51	0.59	5.62	0.89	1.87	43.50	74.64
	40	83.81	606	7.43	4.02	10.19	19.45	11.22	53.14	2.62	2.79	0.96	4.40	0.51	5.22	1.15	1.94	36.59	69.46
$[PXZDPPQ]_2Cu_4I_4$	10	91.37	601	4.63	5.86	9.03	22.59	18.29	50.49	4.88	3.12	2.18	5.65	1.10	5.59	1.59	1.85	44.74	75.37
	15	93.57	602	3.65	7.25	7.29	23.39	22.93	47.25	6.41	3.16	3.17	6.26	1.50	6.48	1.74	1.70	49.50	78.67
	20	89.76	614	3.63	6.78	7.53	22.40	20.48	46.88	6.17	3.02	2.95	5.78	1.38	6.23	1.84	1.73	47.77	77.02
	25	89.16	621	3.56	6.23	8.02	22.14	18.74	48.29	6.22	3.01	2.85	5.55	1.38	6.02	1.99	1.78	45.84	75.74

[a] PL quantum yield (PLQY) measured with integrating sphere; lifetimes of [b] prompt fluorescence (PF), [c] delayed fluorescence (DF) and [d] phosphorescence (PH); efficiencies of [e] PF, [f] DF and [g] PH; rate constants of [h] PF, [i] DF, [j] intersystem crossing (ISC), [k] reverse ISC (RISC), [l] singlet radiation, [m] triplet radiation, [n] singlet and [o] triplet non-radiative transitions; efficiencies of [p] ISC and [q] RISC.

Supplementary Note 5. Electroluminescence Analysis

1. Device fabrication

Materials used for device fabrication were purchased from P-OLED Technology Company, which were used after once more sublimation.

Before loading into a deposition chamber, ITO substrate was cleaned with detergent and deionized water, dried in an oven at 120 °C for 4 h, and treated with oxygen plasma for 3 min. Poly(3,4-ethylenedioxythiophene): poly(styrenesulfonate) (PEDOT:PSS) layer (~40 nm) as hole transporting layer was spin-coated on the substrate. Then, cluster-doped emissive layers were spin-coated on PEDOT:PSS layer. CBP and clusters were dissolved in chlorobenzene with the total concentrations of 15 mg mL^{-1}. The substrate was transferred to ultrahigh-vacuum chamber, and then electron transporting layers were evapored at a rate of 0.1-0.2 nm s^{-1} sequentially at a pressure below 4×10^{-4} Pa. Onto the electron-transporting layer, a layer of LiF with 1-nm thickness was deposited at a rate of 0.1 nm s^{-1} to improve electron injection. Finally, a 100-nm layer of Al was deposited at a rate of 0.6 nm s^{-1} as the cathode. The emission area of the devices was 0.09 cm^2, as determined by the overlapped area of the anode and the cathode. After fabrication, devices were immediately transferred to a glove box for encapsulation with glass coverslips using epoxy glue. The devices were firstly applied with bias from zero to find out the turn-on voltages, and then driven from the turn-on voltages to minimize measurement errors. For each structure, 20 devices were fabricated to determine the reproducibility. The data used were the closest to the average values.

2. Electroluminescence Properties

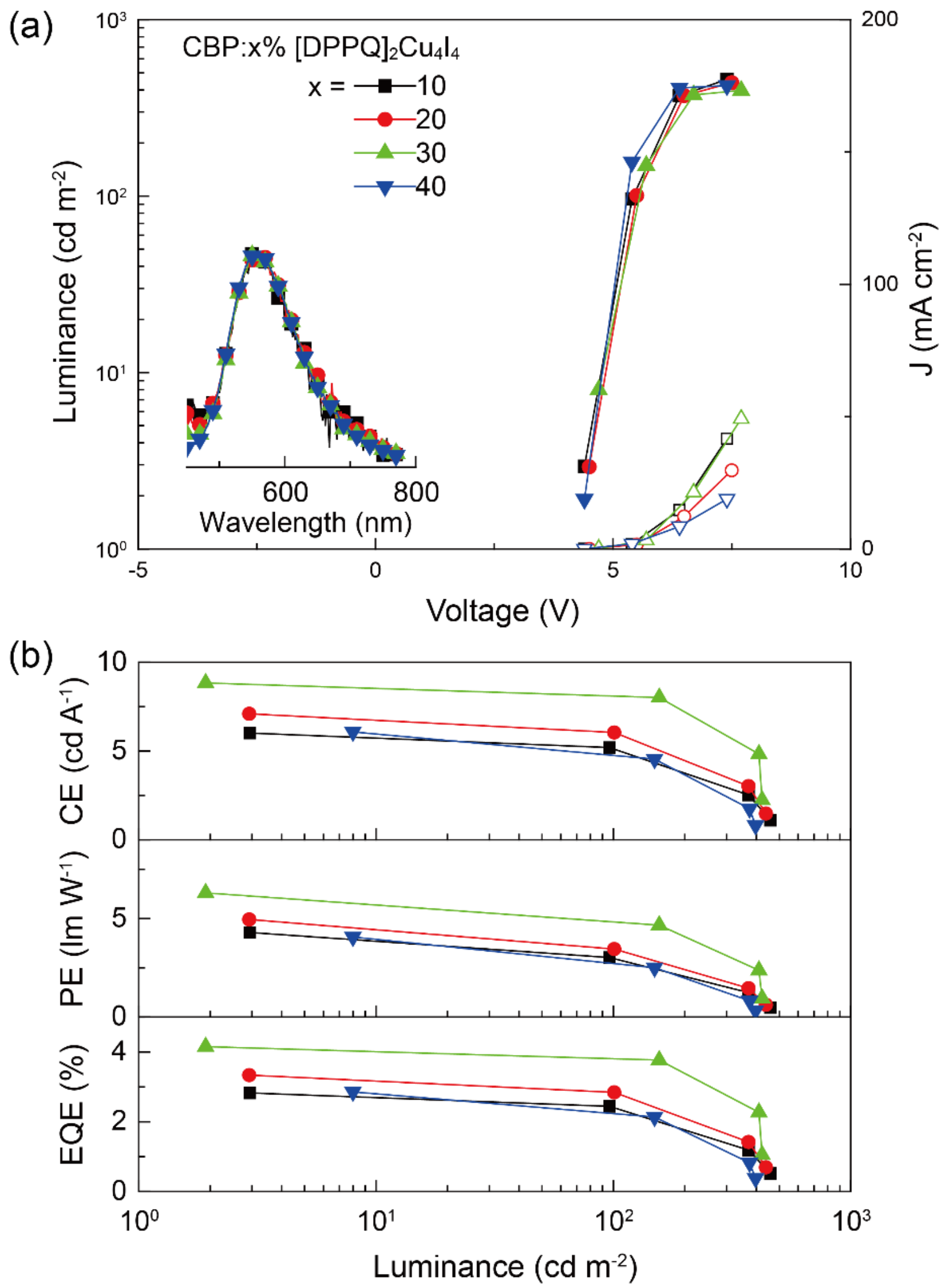

Supplementary Fig. 30 | EL performances of CBP:*x*% [DPPQ]$_2$Cu$_4$I$_4$ based CLEDs with *x*% in the range of 10%-40%. (a) EL spectra (inset) and Current density (*J*)-Voltage-Luminance characteristics. (b) Efficiencies *vs.* Luminance relationships.

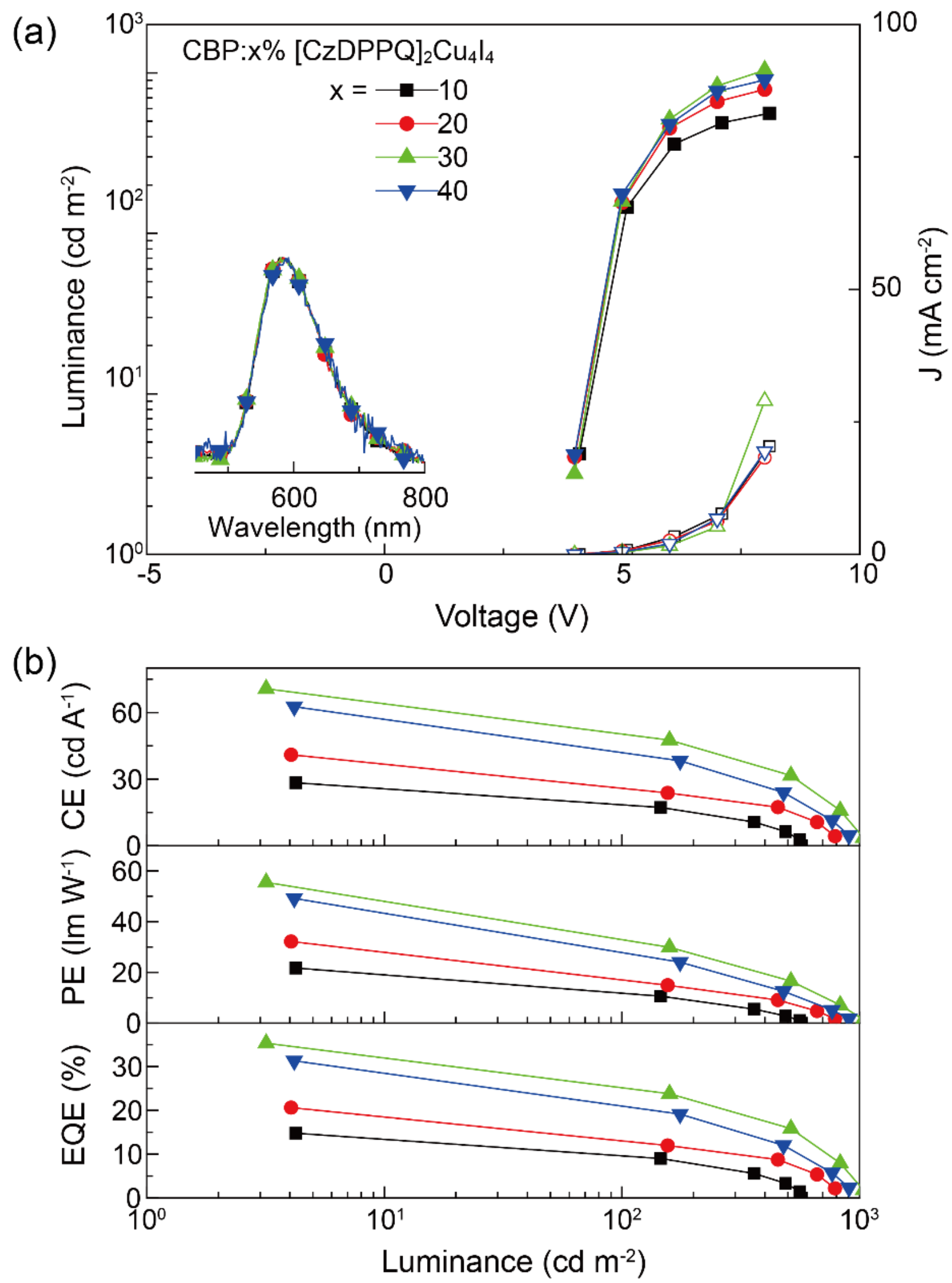

Supplementary Fig. 31 | EL performances of CBP:*x*% $[CzDPPQ]_2Cu_4I_4$ based CLEDs with *x*% in the range of 10%-40%. (a) EL spectra (inset) and Current density (*J*)-Voltage-Luminance characteristics. (b) Efficiencies *vs.* Luminance relationships.

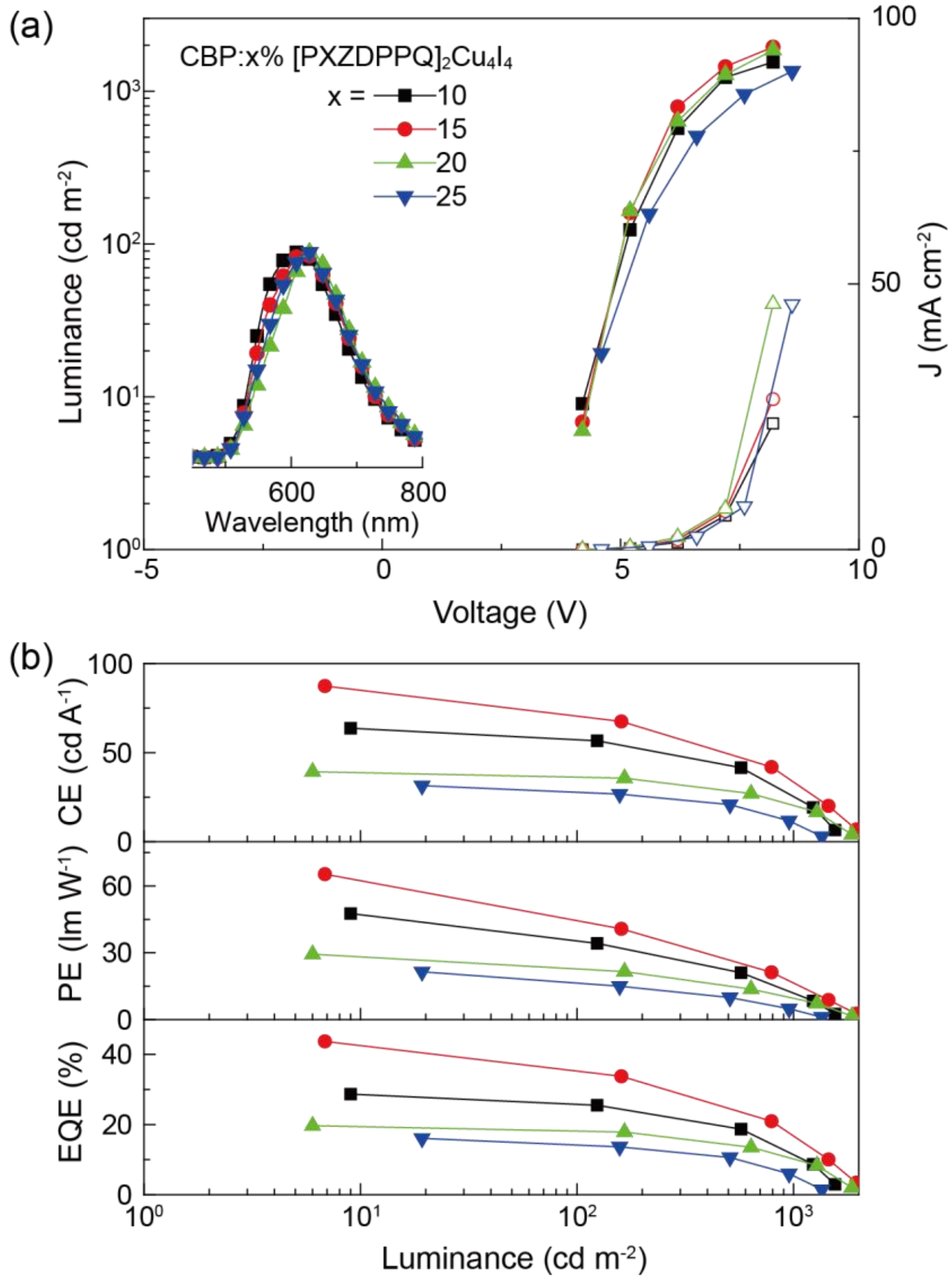

Supplementary Fig. 32 | EL performances of CBP:*x*% $[PXZDPPQ]_2Cu_4I_4$ based CLEDs with *x*% in the range of 10%-25%. (a) EL spectra (inset) and Current density (*J*)-Voltage-Luminance characteristics. (b) Efficiencies *vs.* Luminance relationships.

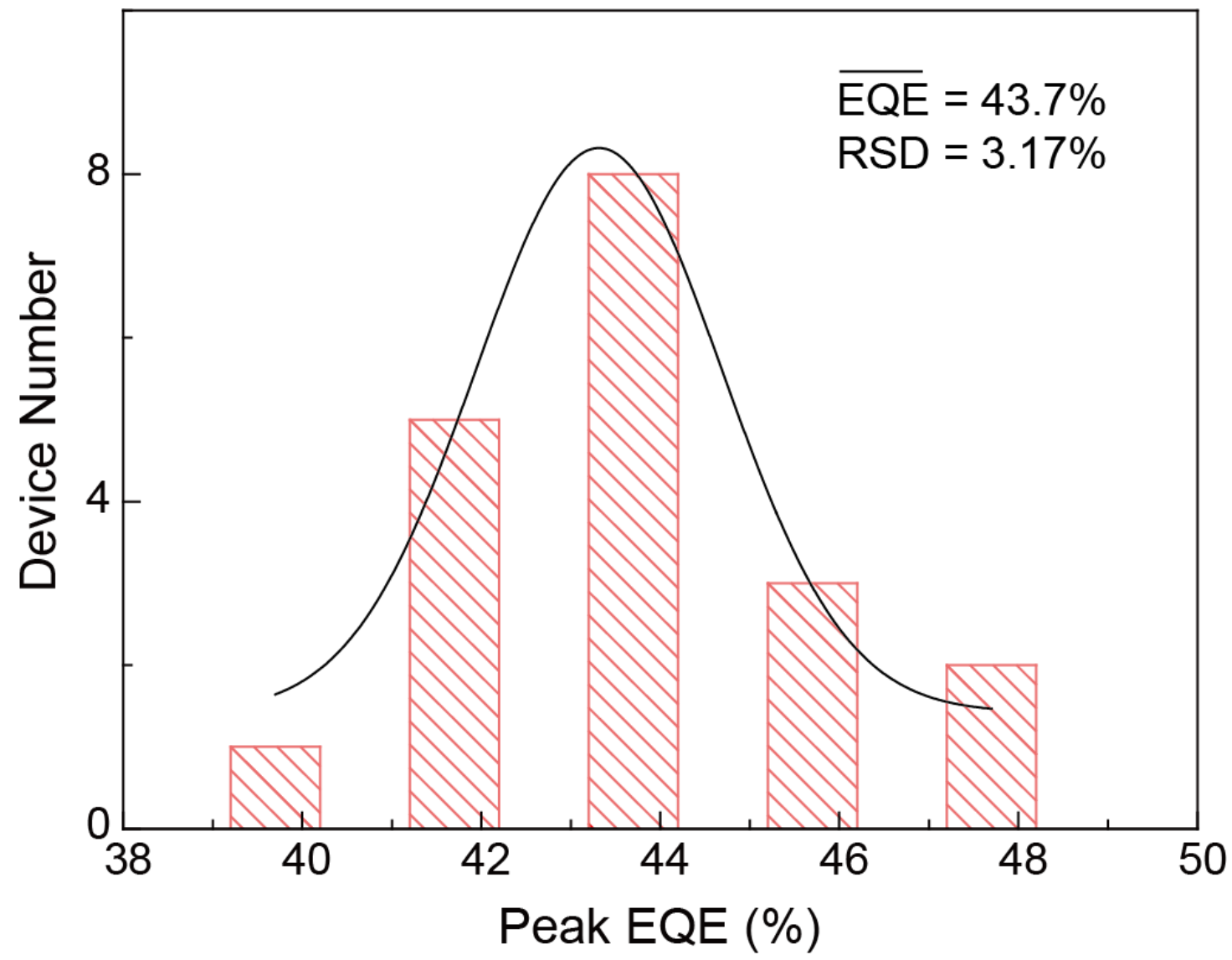

Supplementary Fig. 33 | Statistic analysis of the repeatability for the maximum EQE values of CBP:15% $[PXZDPPQ]_2Cu_4I_4$ based devices with the same structures. Device number is 20. RSD refers relative standard deviation. The EQE variation followed normal distribution with a small RSD less than 5%.

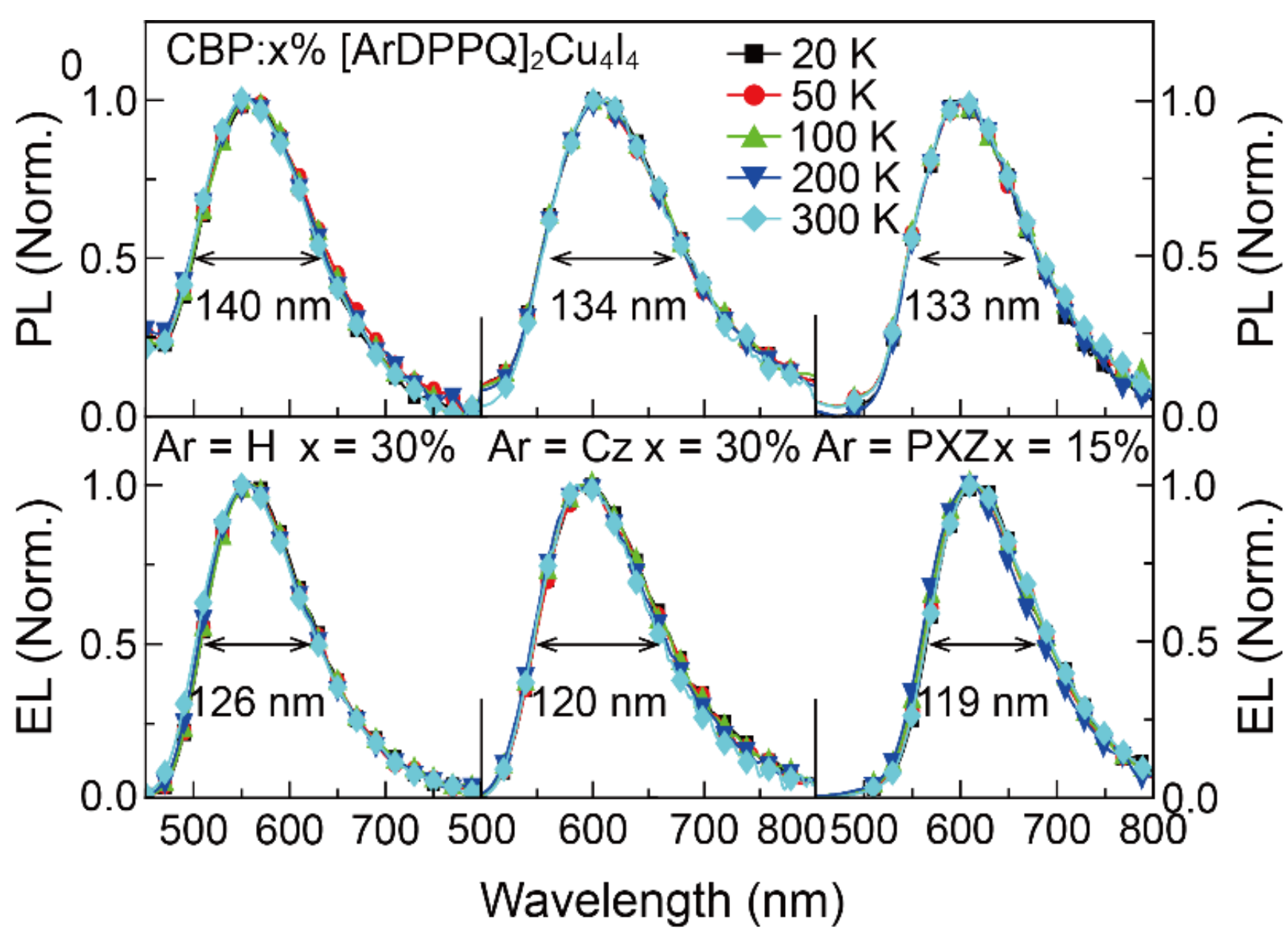

Supplementary Fig. 34 | Comparison on PL (above) and EL (below) spectra of CBP:*x*% clusters at 20, 50, 100, 200 and 300 K.

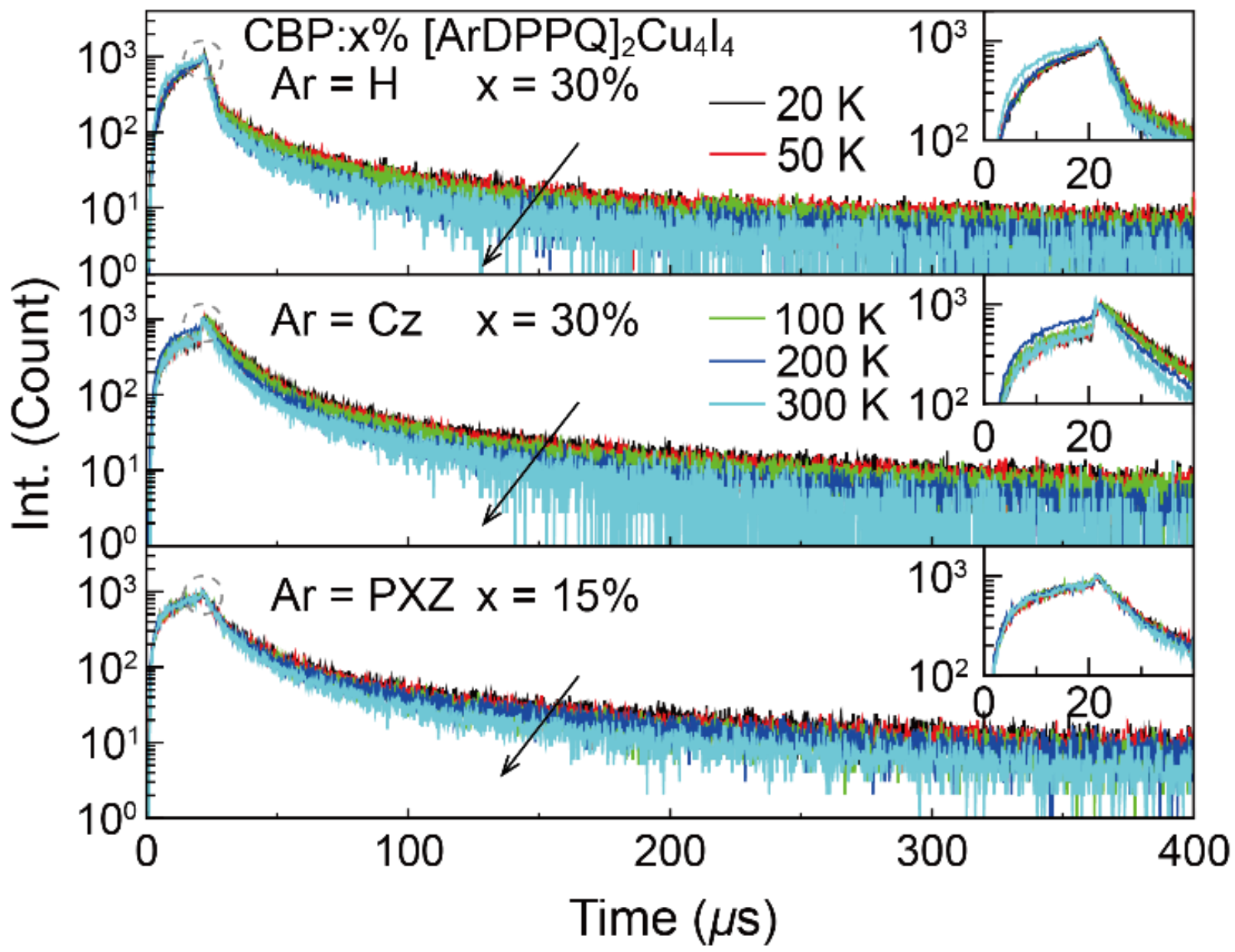

Supplementary Fig. 35 | Electroluminescence time decays of CBP:*x*% clusters at 20, 50, 100, 200 and 300 K. The curves at voltage removal are highlighted in the insets to indicate TPQ process.

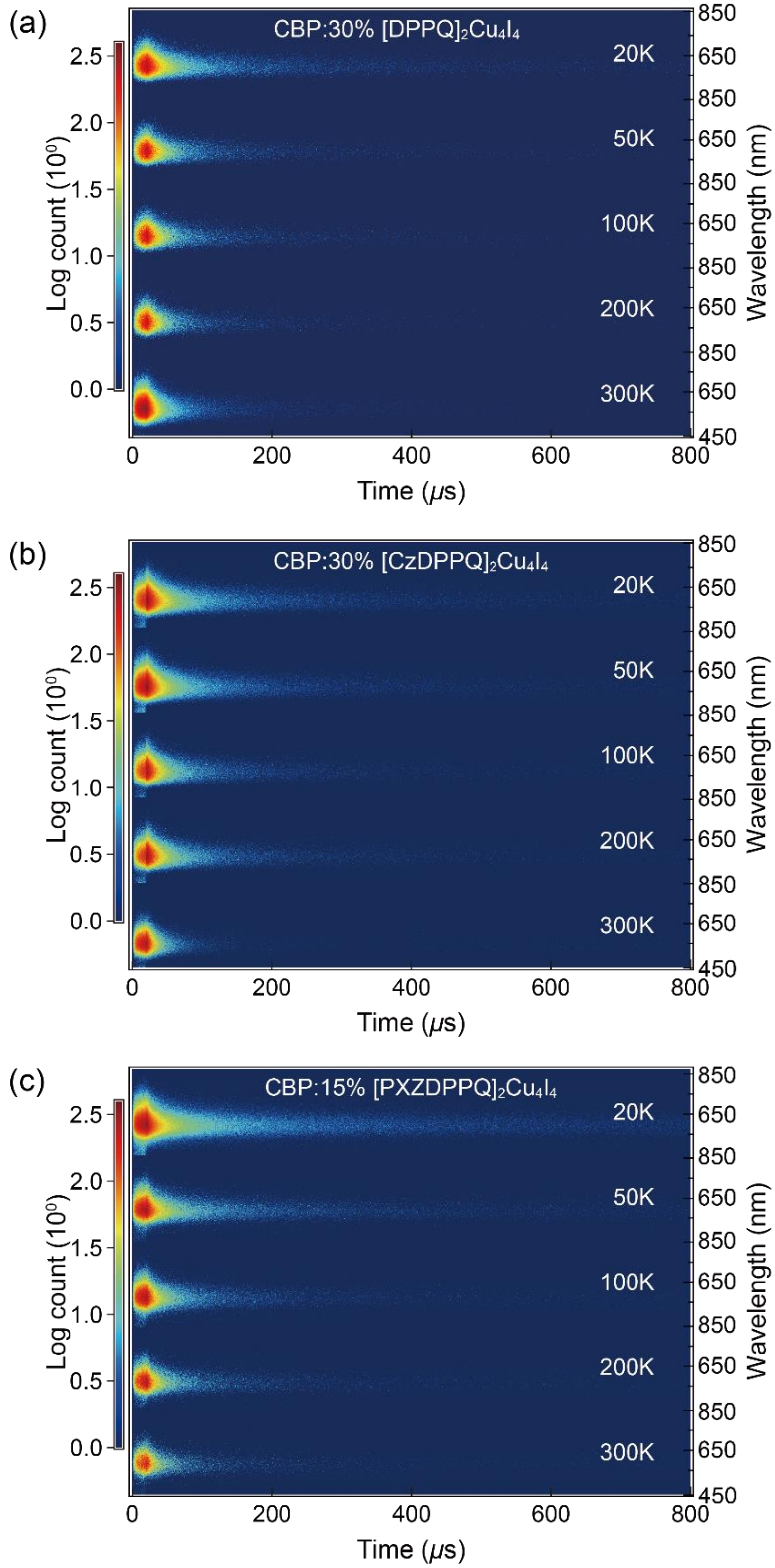

Supplementary Fig. 36 | Temperature dependence of time-resolved electroluminescence emission spectra (TREES) for (a) CBP:30% $[DPPQ]_2Cu_4I_4$, (b) CBP:30% $[CzDPPQ]_2Cu_4I_4$ and (c) CBP:15% $[PXZDPPQ]_2Cu_4I_4$ based devices at 20, 50, 100, 200 and 300 K.

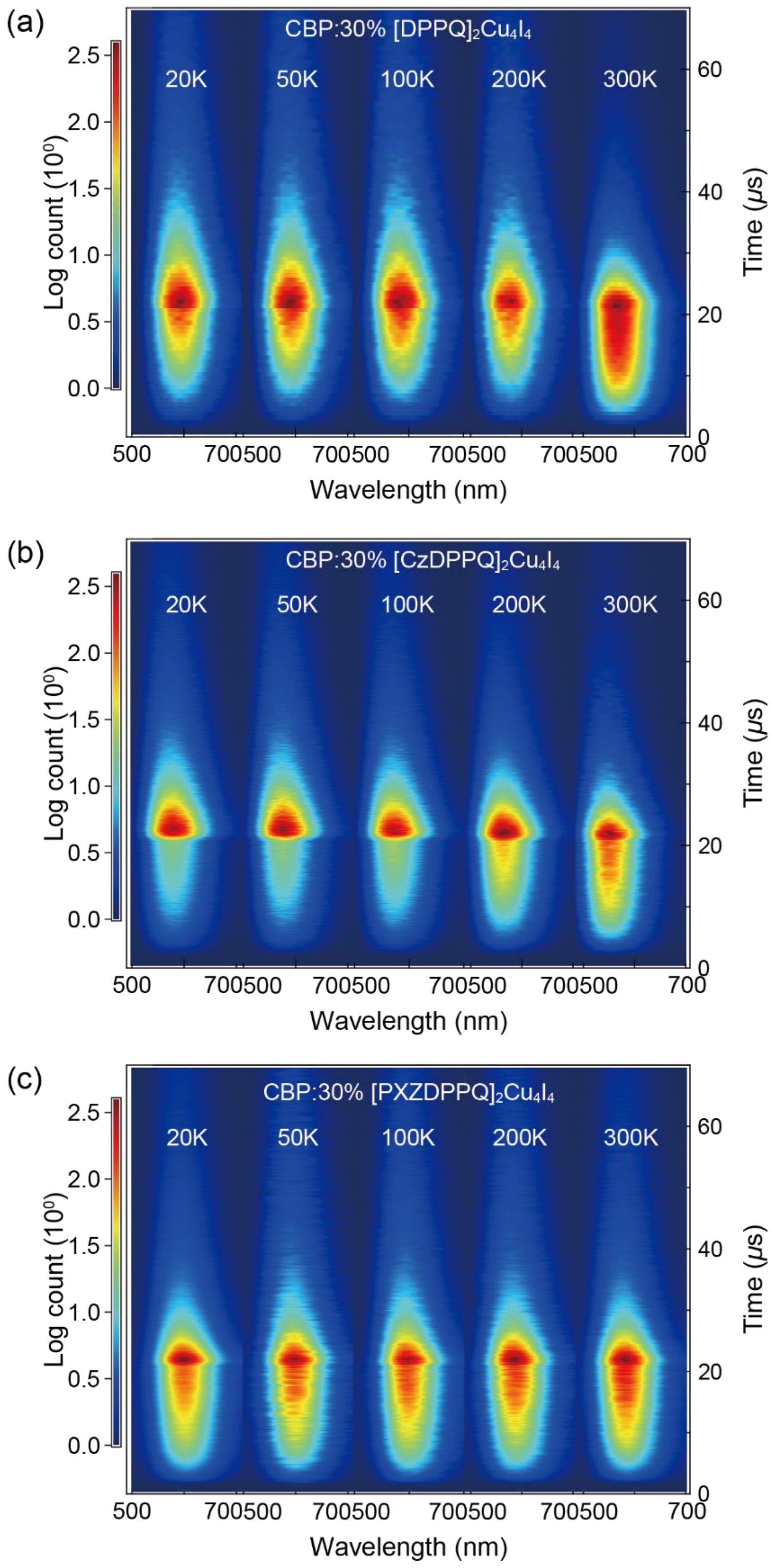

Supplementary Fig. 37 | Temperature dependence of sliced TREES contours during the first 70 μs for (a) CBP:30% $[DPPQ]_2Cu_4I_4$, (b) CBP:30% $[CzDPPQ]_2Cu_4I_4$ and (c) CBP:15% $[PXZDPPQ]_2Cu_4I_4$ based devices at 20, 50, 100, 200 and 300 K. This period contains two stages of carrier recombination during the first 20 μs and exciton decay during the next 50 μs.

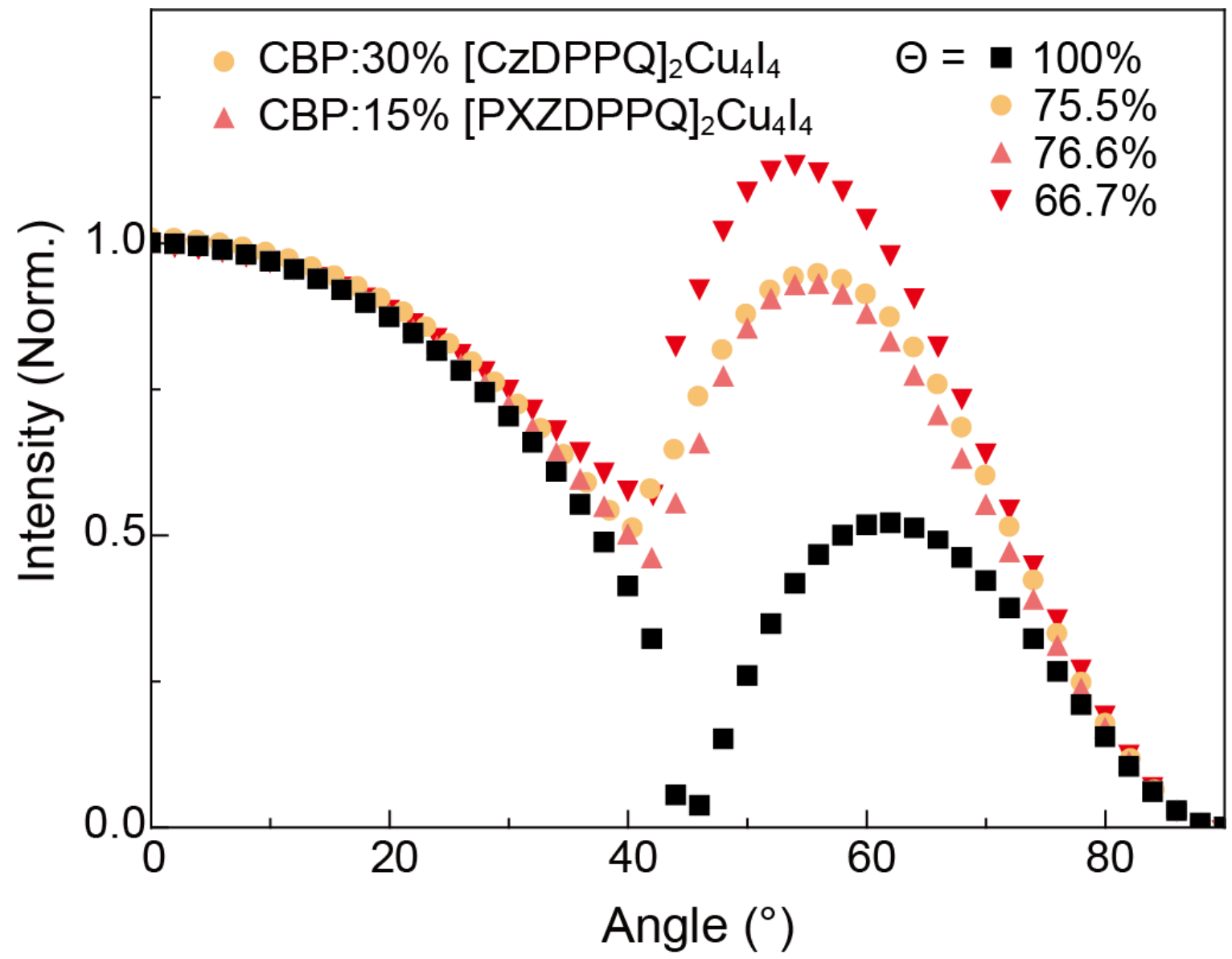

Supplementary Fig. 38 | Experimental p-polarized PL intensities at peak wavelengths of CBP:30% $[CzDPPQ]_2Cu_4I_4$ and CBP:15% $[PXZDPPQ]_2Cu_4I_4$ films, which are compared to simulated curves lines with different horizontal dipole ratios (Θ). Θ = 100% for fully horizontal dipoles, and 66.7% for the isotropic dipole orientation.

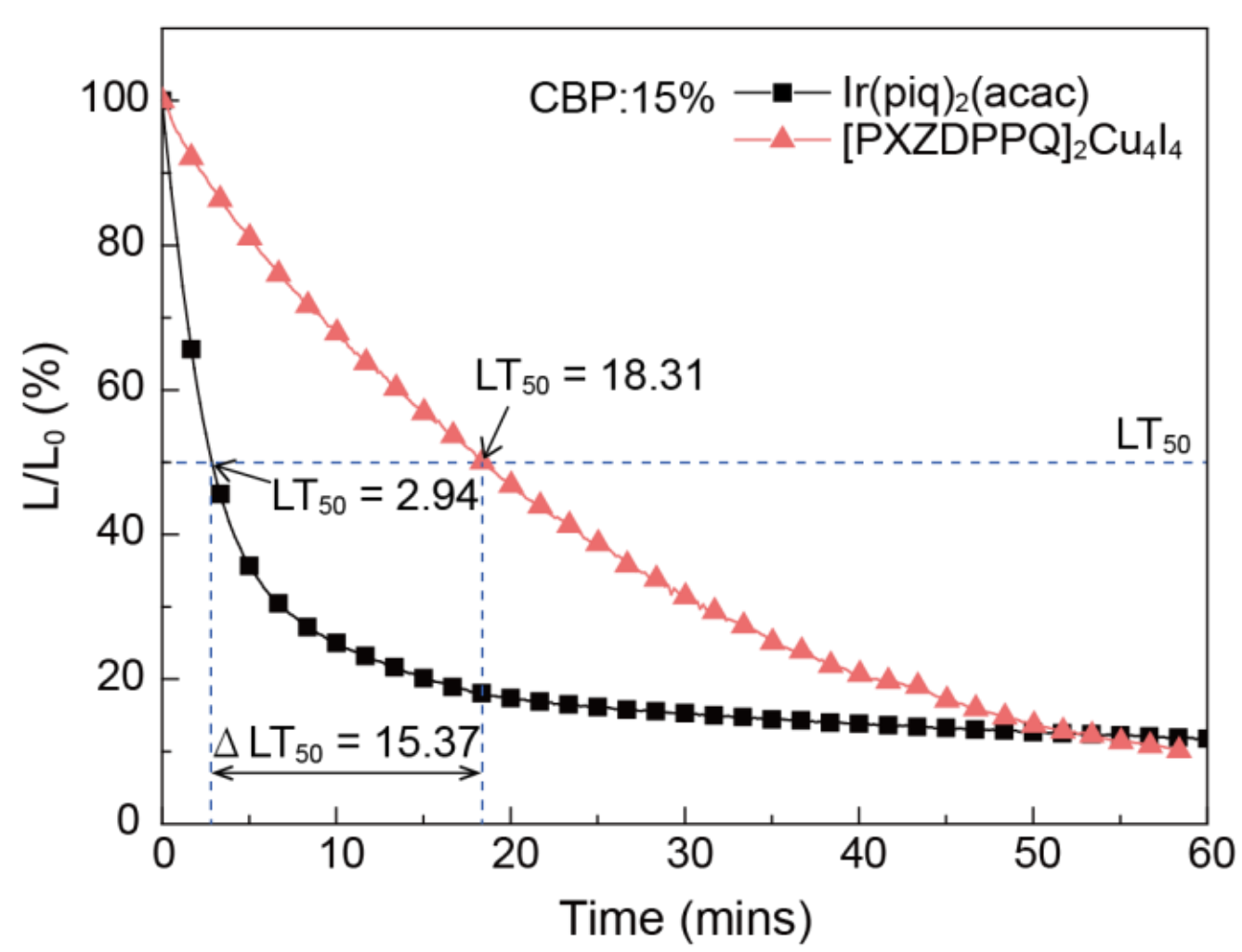

Supplementary Fig. 39 | Aging curves of the devices based on $[PXZDPPQ]_2Cu_4I_4$ under constant-current operation. LT_{50} represents the time at which the luminescence decreased to 50% of the initial value. $Ir(piq)_2acac$ (piq = 1-phenyl-isoquinoline, acac = acetylacetone) is a commercial Ir^{3+} phosphor for red-emitting OLEDs.

Supplementary Table 4 | EL performance of CBP:*x*% clusters based devices.

EML	*x* (wt%)	$V^{[a]}$ (V)	L_{max}[b] (cd m^{-2})	η[c] η_{CE} (cd A^{-1})	η_{PE} (lm W^{-1})	η_{EQE} (%)	λ_{EL} (nm) / CIE (x, y)[d]
CBP:*x*% $[DPPQ]_2Cu_4I_4$	10	4.4, 5.1, -	459	6.0, 5.3, -	4.3, 3.0, -	2.8, 2.5, -	553/(0.40, 0.44)
	20	4.5, 5.2, -	439	7.1, 6.0, -	5.0, 3.5, -	3.3, 2.8, -	555/(0.41, 0.43)
	30	4.4, 5.2, -	423	8.8, 8.1, -	6.3, 5.0, -	4.2, 3.8, -	556/(0.41, 0.43)
	40	4.7, 5.2, -	397	6.1, 4.9, -	4.1, 2.7, -	2.9, 2.2, -	556/(0.42, 0.43)
CBP:*x*% $[CzDPPQ]_2Cu_4I_4$	10	4.1, 5.0, -	560	28.3, 18.1, -	21.7, 11.6, -	14.8, 9.7, -	585/(0.43, 0.51)
	20	4.0, 4.9, -	789	41.0, 26.0, -	30.2, 16.4, -	20.6, 11.6, -	585/(0.43, 0.51)
	30	4.0, 4.9, 8.1	1039	70.7, 50.0, 1.9	55.5, 33.3, 0.9	35.3, 25.0, 1.4	586/(0.44, 0.50)
	40	4.0, 4.9, -	904	62.6, 42.1, -	49.1, 27.1, -	31.3, 20.4, -	586/(0.44, 0.50)
CBP:*x*% $[PXZDPPQ]_2Cu_4I_4$	10	4.2, 5.1, 7.0	1555	63.7, 57.6, 24.6	47.6, 35.7, 12.8	28.7, 25.6, 12.3	613/(0.49, 0.46)
	15	4.2, 5.0, 6.6	1947	87.4, 70.9, 34.0	65.3, 44.8, 15.6	43.7, 35.3, 16.3	615/(0.59, 0.37)
	20	4.2, 5.1, 6.9	1860	39.3, 36.9, 20.6	29.4, 23.5, 10.1	19.7, 18.3, 10.3	621/(0.60, 0.35)
	25	4.6, 5.4, 7.7	1352	31.4, 27.1, 9.3	21.5, 16.0, 3.4	16.1, 13.8, 4.8	628/(0.61, 0.35)

[a] Operation voltages for turn on, and at 100 and 1000 cd m^{-2}; [b] the maximum luminance; [c] EL efficiencies at the maximum, 100 and 1000 cd m^{-2}; [d] EL peak wavelength and CIE coordinates.

Supplementary Table 5 | Comparison on EL performances of representative clusters.

Device structure	Cluster	V_{on}[a] (V)	L_{max}[b] ($cd \cdot m^{-2}$)	η_{max}[c]			CIE (x, y)	λ_{EL}[d] (nm)	Ref.
				η_{CE} (cd A^{-1})	η_{PE} (lm W^{-1})	η_{EQE} (%)			
ITO | PEDOT:PSS (30 nm) | TCTA:$[Au_4(P\^{}P\^{}P)_2(C_2C_6H_4NMe_2)_2](PF_6)_2$ (50 nm) | TPBi (50 nm) | LiF (0.5 nm) |Al (100 nm)	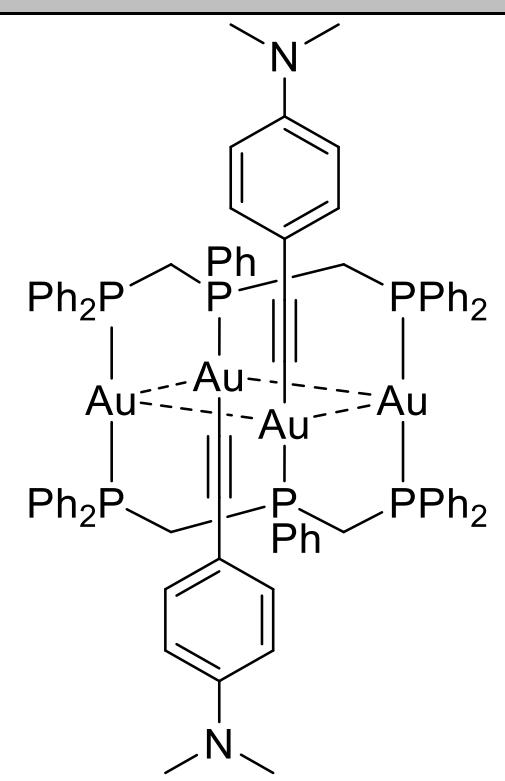	2.5	7430	6.1	5.3	3.1	(0.52, 0.46)	600	7
ITO | PEDOT:PSS (30 nm) | TmPyPB: $[TMeOPP]_4Cu_4I_4$ (40 nm) | TmPyPB (40 nm) | LiF (1 nm) |Al (100 nm)	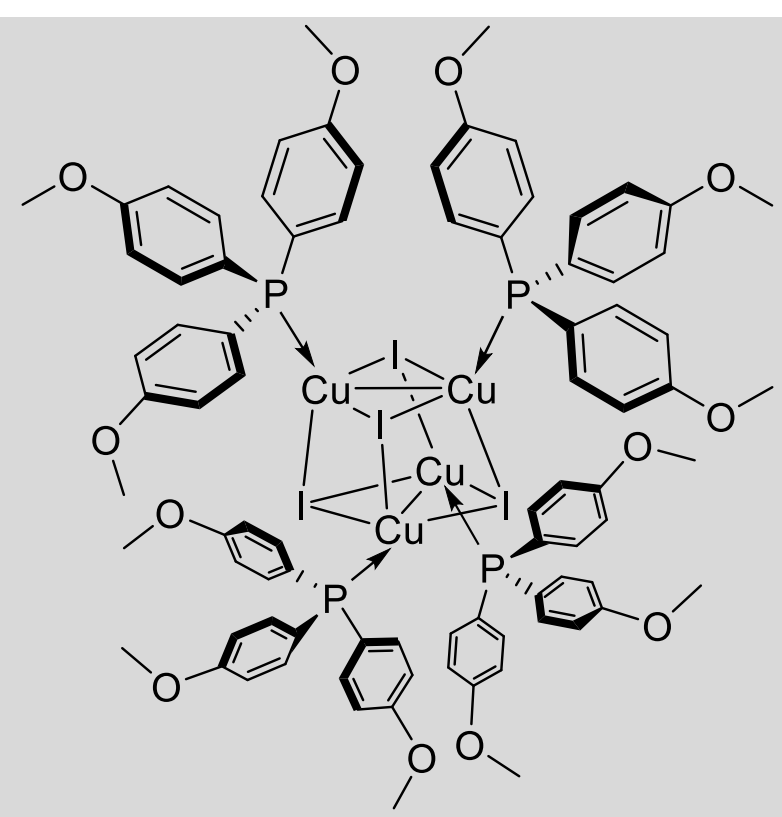	3.0	10710	52.8	55.3	15.6	(0.39, 0.56)	550	8

ITO | PEDOT:PSS (50 nm)|CuSCN (30 nm)|TCTA: OXD-7: $[Ag_6Cu[CH(PPh_2)_3]_2(Ph_2PO_2)_3(CRC\text{-}2\text{-}Phcarb\text{-}9)_3](ClO_4)$ (57 wt%, 28 wt%, 15 wt%, 50 nm) / TPBi (50 nm) | LiF (1 nm) | Al (100 nm)	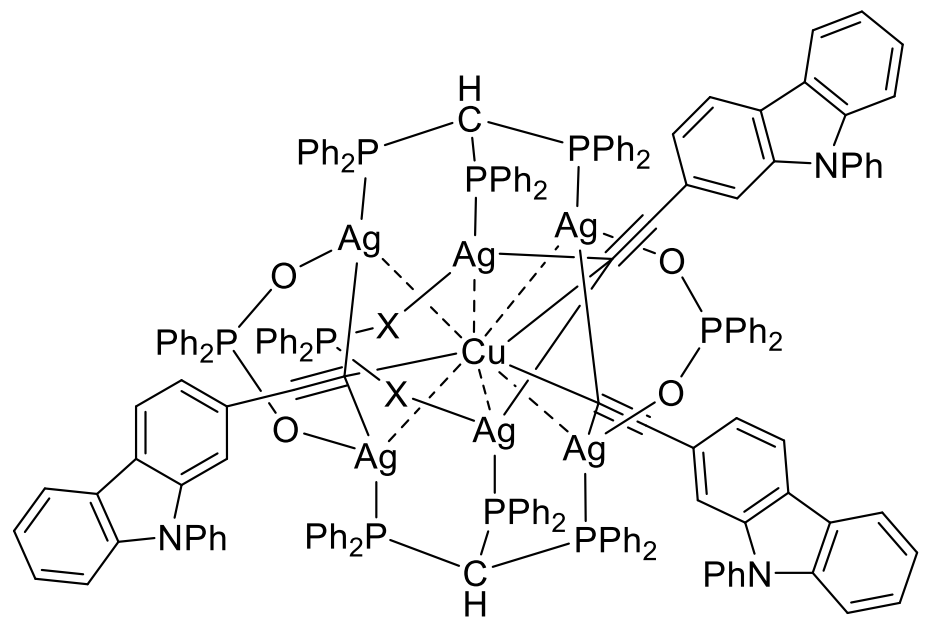	3.4	500	42.5	30.0	13.9	--	573	9
ITO | PEDOT:PSS (50 nm) | TAPC:OXD-7:$PtAu_2(dpmp)_2$(C≡C-PTZ-Et)$_2$ (46 wt%, 46 wt%, 8 wt%, 50 nm) | TPBi (50 nm) | LiF (1 nm) | Al (100 nm)	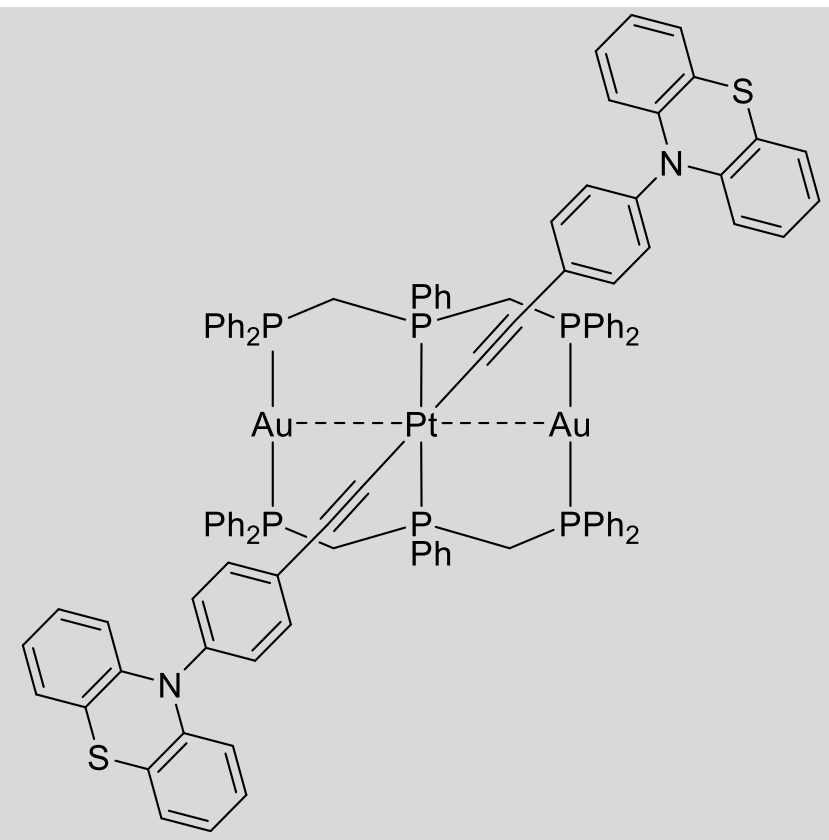	4.5	10446	35.4	16.8	18.7	(0.51, 0.49)	605	10
ITO | PEDOT:PSS (50 nm) | CuSCN (30 nm)|2,6-DCZPY: OXD-7:[$PtAg_2$(rac-dpmppe)(C≡C-PTZ-Et)$_2$[P(Et-9-carb-3)$_3$]$_2$]$(ClO_4)_2$ (70.5 wt%, 23.5 wt%, 8 wt%, 50	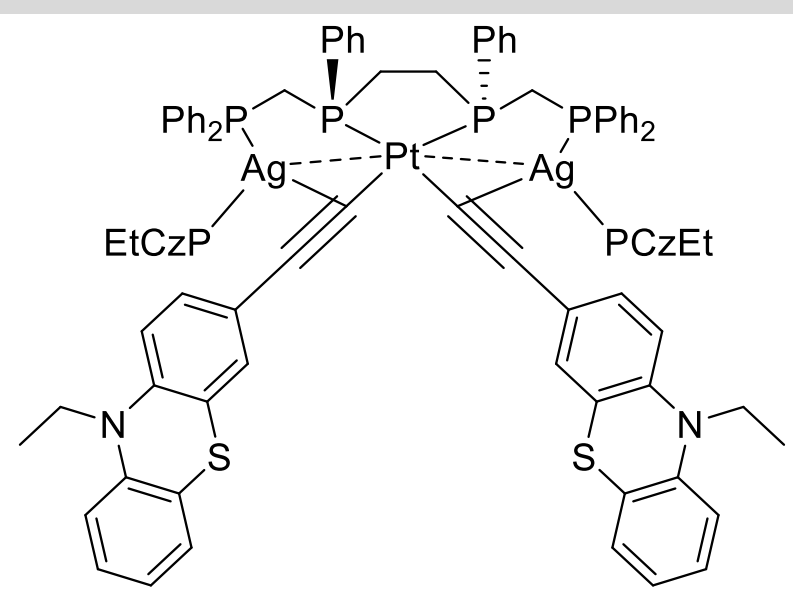	4.6	1898	19.8	9.9	12.4	(0.53, 0.46)	616	11

nm) | BmPyPb (50 nm) | LiF (1 nm) | Al (100 nm)									
ITO | PEDOT:PSS (50 nm) | mCP:OXD-7:[$PtAu_3$(l-Cl)(tdpmp)$_2$(CRC-3-EtPTZ-9)$_2$](ClO_4)$_2$ (48.5 wt%, 48.5 wt%, 3 wt%, 50 nm) | BmPyPb (50 nm) | LiF(1 nm) | Al (100 nm)	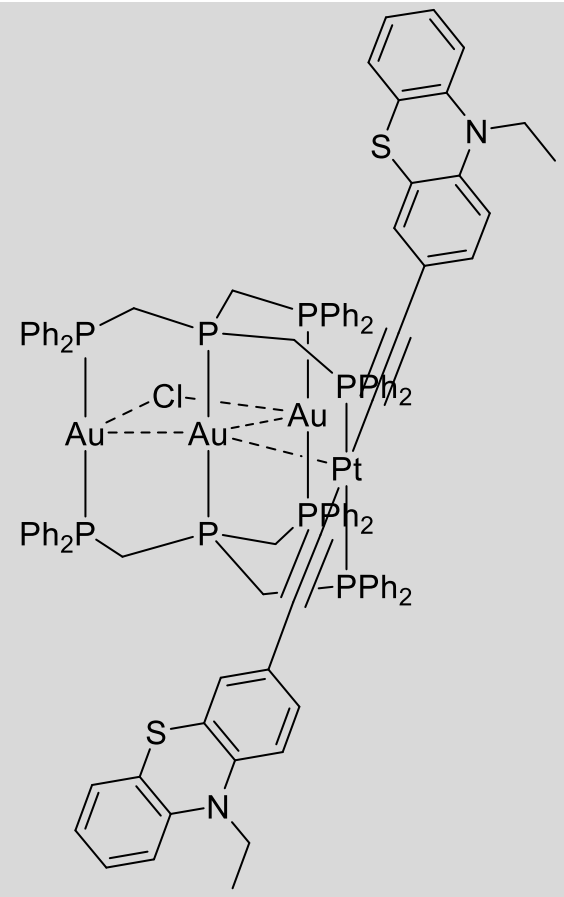	4.9	19308	45.2	20.3	18.1	(0.47, 0.52)	588	[12]
ITO | PEDOT:poly-TPD (50 nm)|mCP:OXD-7:[$PtAu_3$(l-Cl)(tdpmp)$_2$(CRC-3-Spirocarb(NMe))$_2$](ClO_4)$_2$ (48 wt%, 47 wt%, 5 wt%, 50 nm) | BmPyPb (50 nm) | LiF (1 nm) | Al (100 nm)	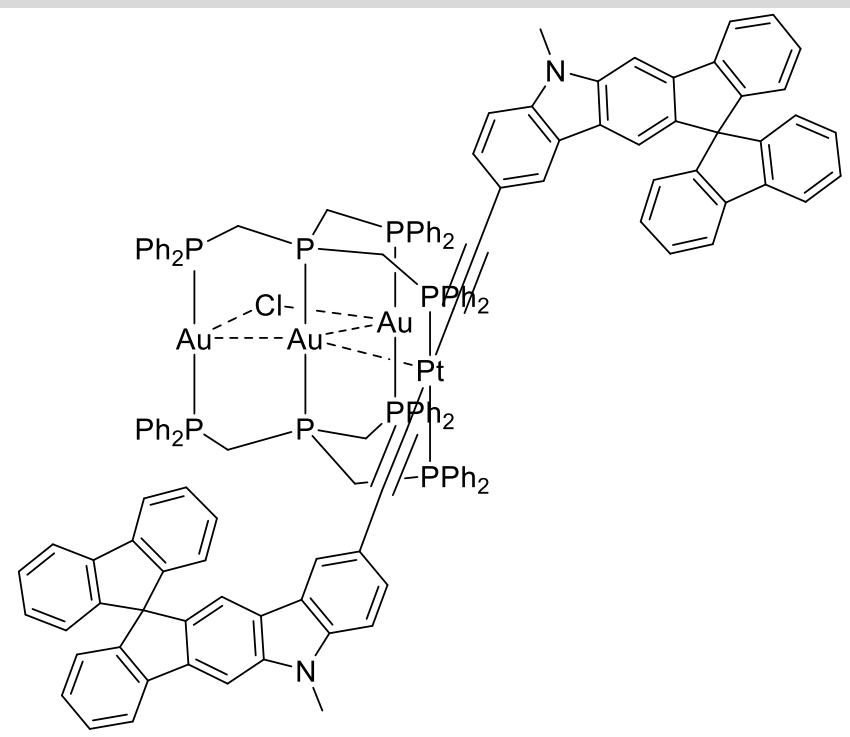	5.4	12702	57.1	20.1	13.1	(0.46, 0.54)	568	[13]

ITO | PEDOT:PSS (50 nm) | [Pt$_2$Au(dpmp)$_2$(DECz)(C≡CPh)$_2$](ClO$_4$):mCP:OXD-7 (6 wt%, 62.6 wt%, 31.3 wt%, 50 nm) | BmPyPb (50 nm) | LiF (1 nm) | Al (100 nm)	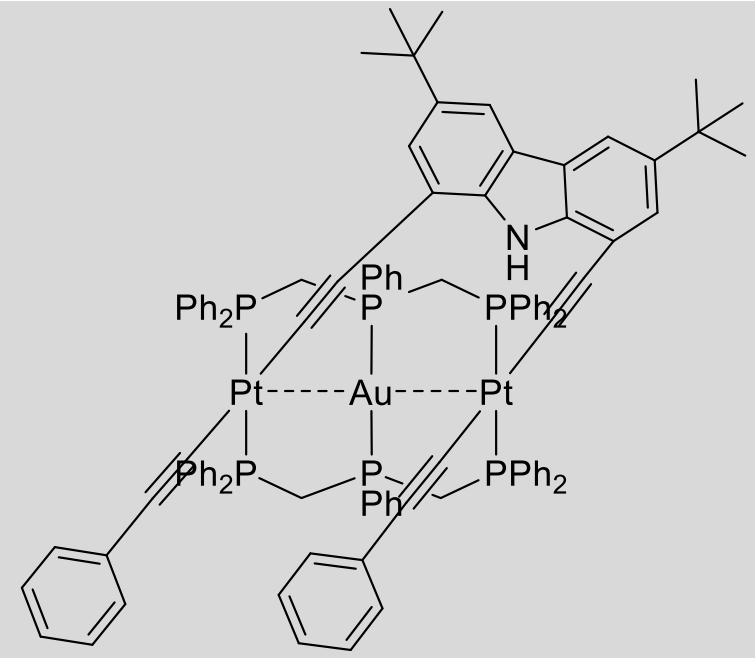	3.1	37160	64.6	26.1	18.3	(0.34, 0.36)	568	14
ITO | PEDOT:PSS (40 nm) | PCTSQ: DSOBPy-Cu (90 wt%, 10 wt%, 50 nm) | TAZ (30 nm) | Al (100 nm)	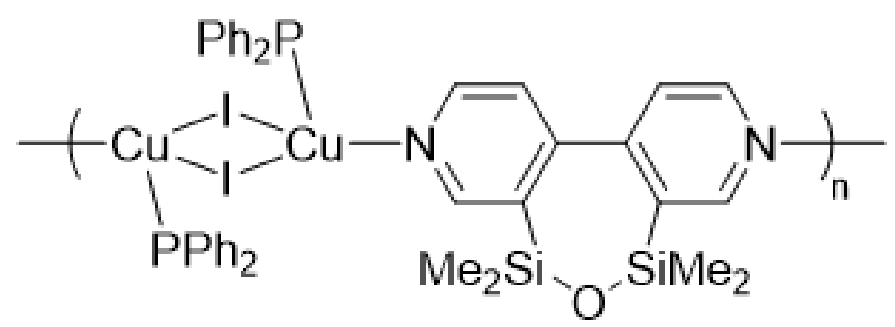	5.7	15081	38.1	44.3	11.9	(0.38, 0.49)	618	15
ITO | PEDOT:PSS (50 nm) | PolyTPD (30 nm) | TCTA:OXD-7:[Ag$_3$Cu$_5$(ebzcz)$_6$(dpppy)$_3$](ClO$_4$)$_2$ (47.5 wt%, 47.5 wt%, 5 wt%, 50 nm) | BmPyPb (50 nm) | LiF (1 nm) | Al (100 nm)	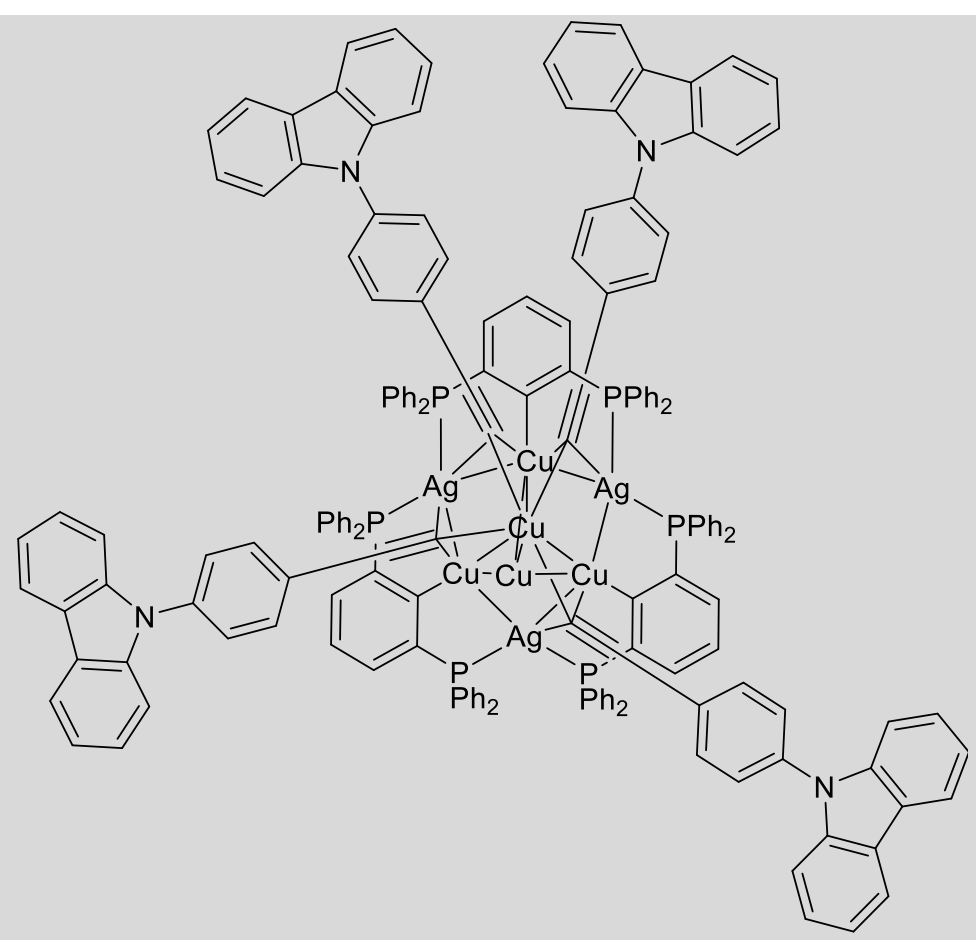	3.2	8554	38.1	29.9	14.7	(0.51, 0.48)	585	16

ITO | PEDOT:PSS (40 nm) | poly-TPD (15 nm) | TCTA: OXD-7:[$PtAg_2(dTolmp)_2(C{\equiv}CTPA)_2](ClO_4)_2$ (47 wt%, 47 wt%, 6 wt%, 50 nm) | ETL (50 nm) | LiF (1 nm) | Al (100 nm)	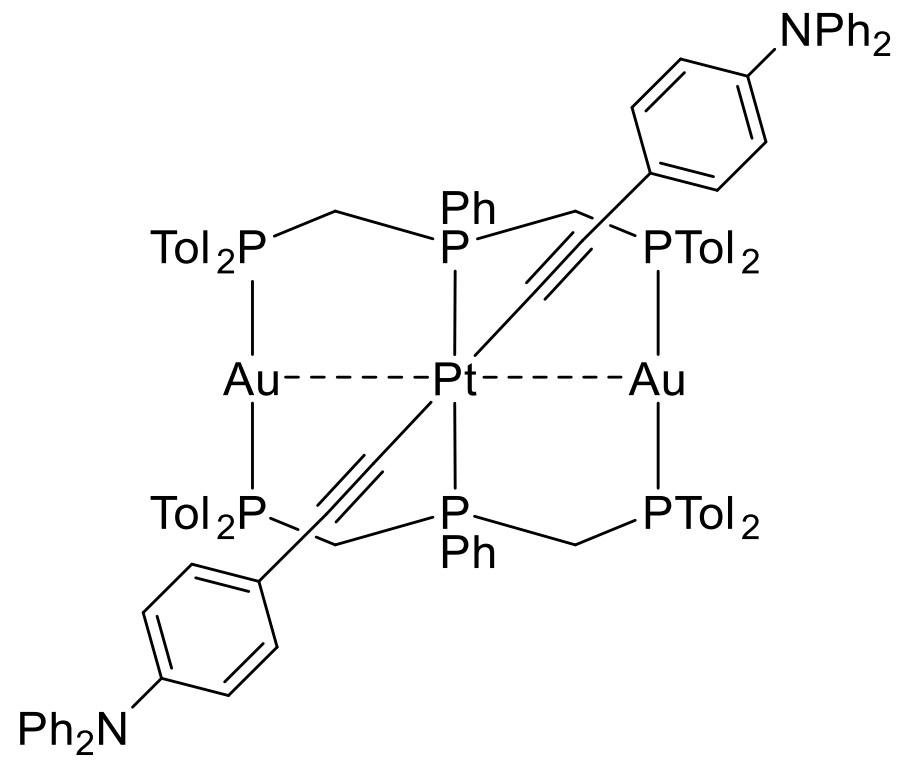	3.8	6117	69.1	37.8	21.7	(0.49, 0.50)	579	17
ITO | PEDOT:PSS (40 nm) | Poly-TPD (50 nm)|mCP: OXD-7:$PtAu_2(dTolmp)_2$(C≡C-4-ph -4H-dithieno[3,2-b:2',3'-d]pyrrole)$_2$ $_2$ (47.5 wt%, 47.5 wt%, 5 wt%, 50 nm) | BmPyPb | LiF (1 nm) | Al (100 nm)	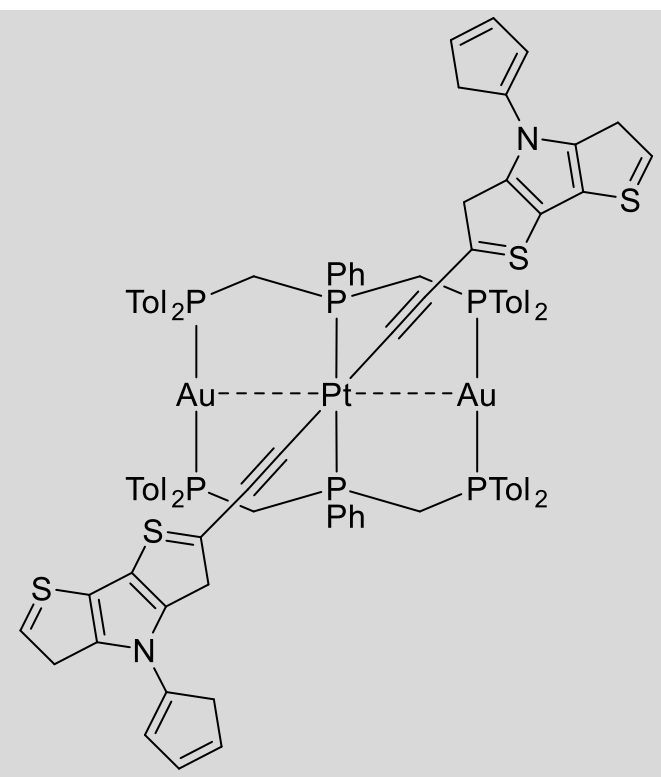	5.2	4034	13.9	8.0	13.6	(0.67, 0.33)	625	18
ITO | PEDOT:PSS (50 nm) | mCP:S-ql-I (98 wt%, 2 wt%, 40 nm)| DPEPO (10 nm) | TmPyPB (50 nm)|Liq (1 nm)|Al (100 nm)	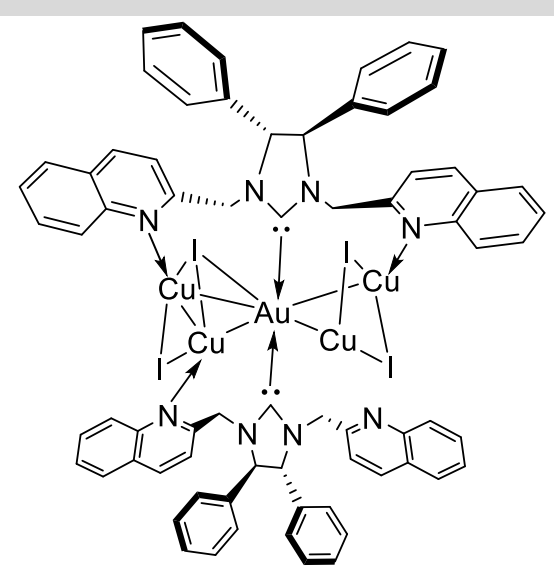	2.7	5600	59.2	23.3	20.8	(0.51, 0.47)	586	19

ITO | PEDOT:PSS (50 nm) | [$PtAu_2$(μ-dTolmp)$_2$(C≡C-NAI-CzEt)$_2$](CF_3SO_3)$_2$:TAPC:OXD-7 (3 wt%, 49 wt%, 48 wt%, 50 nm) | BmPyPb (50 nm) | LiF (1 nm) | Al (100 nm)	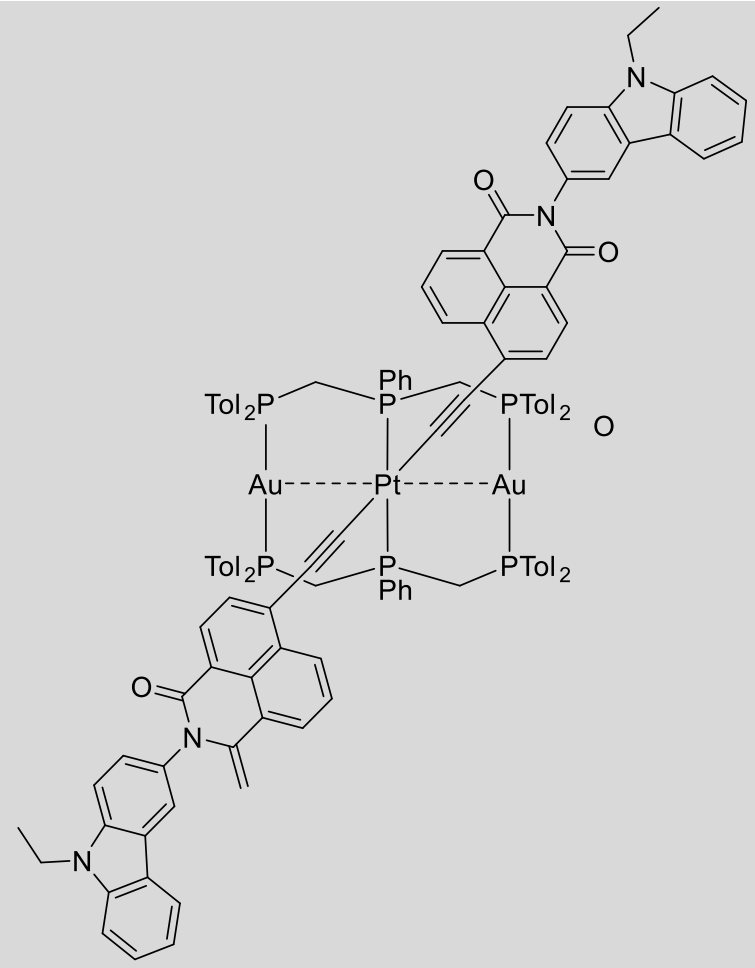	4.0	1003	15.0	11.5	16.7	(0.69, 0.31)	625	[20]
ITO | PEDOT:PSS (50 nm) | Poly-TPD (15 nm) | R-$PtAu_2$:TCTA:OXD-7 (10wt%, 45 wt%, 45 wt%, 50 nm) | TmPyPB (40 nm) | LiF (1 nm) | Al (100 nm)	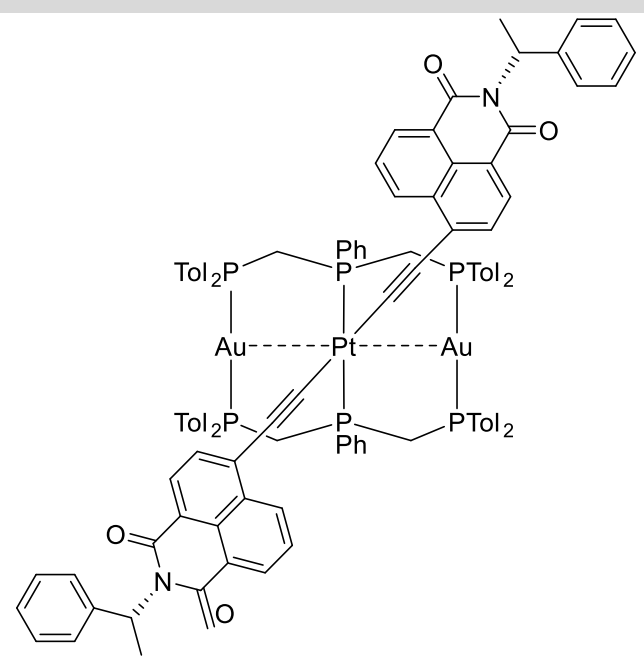	3.1	11000	14.5	12.6	13.7	(0.68, 0.32)	625	[21]

ITO | PEDOT:PSS (50 nm) | Poly-TPD (10 nm)|R-Ag_6Cu: 2,6-DCzPPy:OXD-7 (40 wt%, 30 wt%, 30 wt%, 50 nm) | BmPyPb (40 nm) | LiF (1 nm) | Al (100 nm)	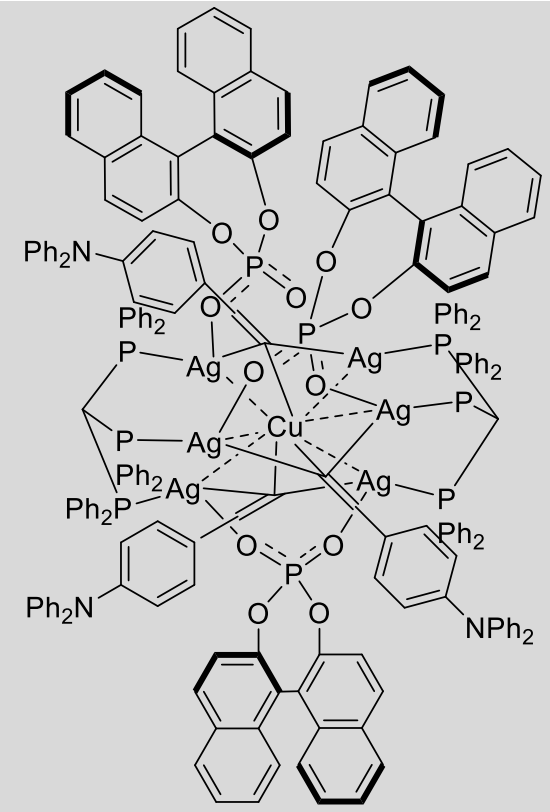	3.4	1053	26.4	22.9	26.7	(0.65, 0.35)	653	22
ITO | PEDOT: PSS (50 nm) | PTAA (20 nm) | $Au_{16}Cu_6(^{t}BuC_6H_4C\equiv C)_{18}$:PVK:OXD-7 (4 wt%, 57.6 wt%, 38.4 wt%, 50 nm)) | TmPyPB (80 nm) |LiF (0.8 nm) | Al (120 nm)	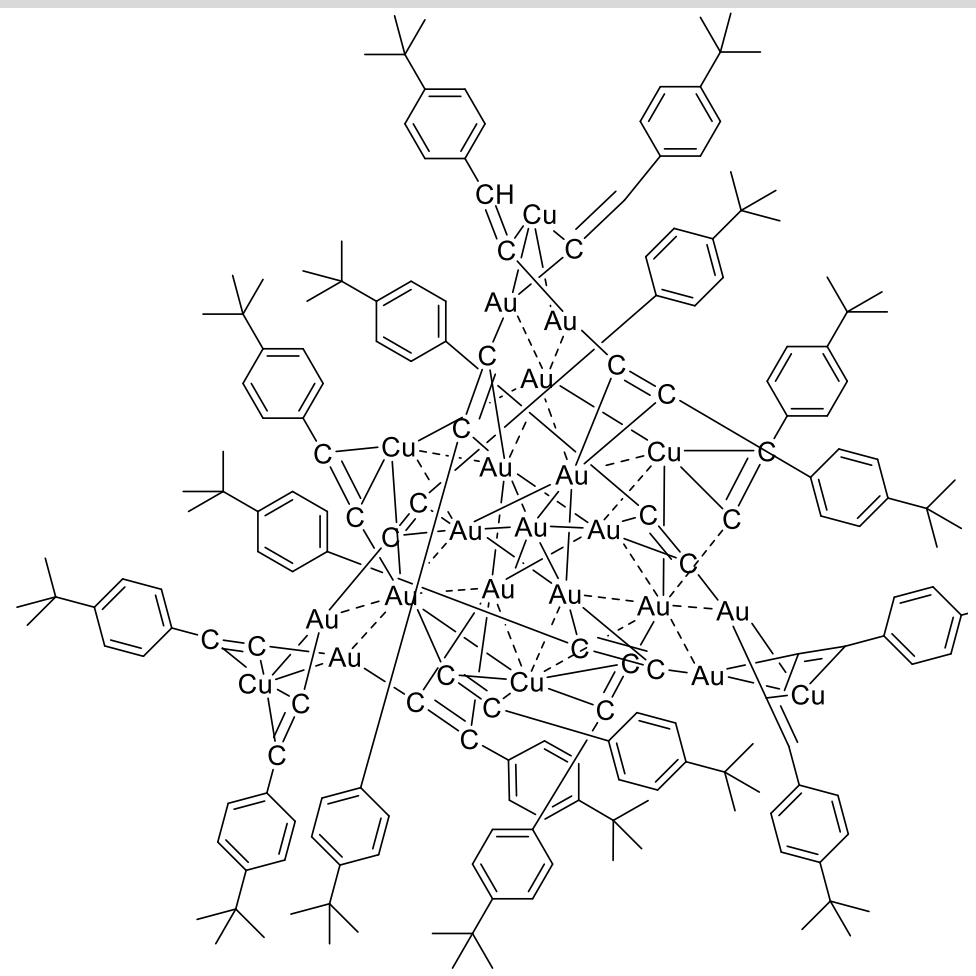	4.6	--	--	--	15.4	--	706	23

[a] Turn-on voltages; [b] the maximum luminance; [c] the maximum efficiencies; [d] EL peak wavelength.

Supplementary Table 6 | Comparison on performances of recently reported representative red-emitting emitters.

Material Type	Device Structure	Emitter	η_{PL}[a] (%)	η[b]			λ_{EL}[c] (nm)	Ref.
				η_{CE} (cd A^{-1})	η_{PE} (lm W^{-1})	η_{EQE} (%)		
Radical	ITO/MoO_3 (3 nm)/TAPC (30 nm)/TCTA (10 nm)/MADN:TTM-TPA 3 wt% (20 nm)/B3PYMPM (80 nm)/LiF (1 nm)/Al (100 nm)	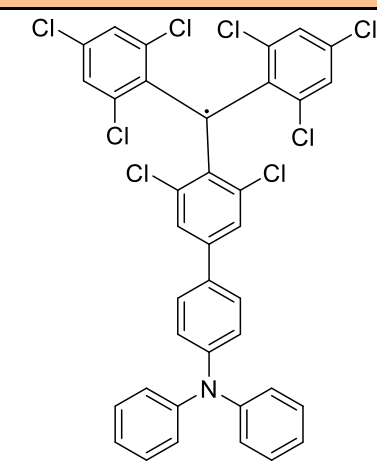 TTM-TPA	27.00	-	-	9.60	800	24
Multi-Resonance	ITO/TAPC (50 nm)/TCTA (10 nm)/DMIC-TRZ:0.5 wt% 2S-BN (30 nm)/TmPyPb (30 nm)/LiF (1 nm)/Al (100 nm)	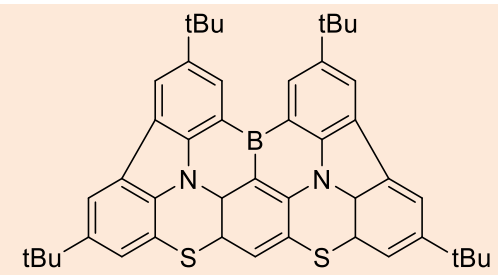 2S-BN	98.40	4.20	4.40	29.30	676	25
	ITO/TAPC (50 nm)/TCTA (10 nm)/DMIC-TRZ:1 wt% tFSBN (30 nm)/TmPyPB (35 nm)/LiF (1 nm)/Al (100 nm)	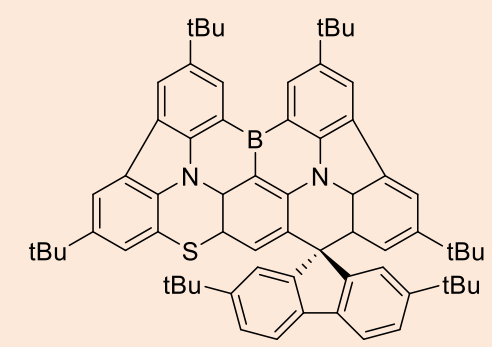 tFSBN	98.00	41.90	48.70	35.20	620	26
Thermally Activated Delayed Fluorescence (TADF) Emitter	ITO/PEDOT:PSS (40 nm)/F8BT:0.2 wt% eDPP (125 nm)/Ca (30 nm)/Al (200 nm)	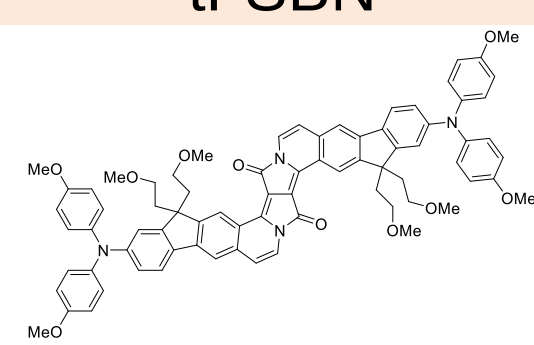 eDPP	33.00	-	-	2.72	670	27

	ITO/PEDOT:PSS (40 nm)/PNAI-AcCz (40 nm)/TmPyPB (42 nm)/LiF (1 nm)/Al (100 nm)	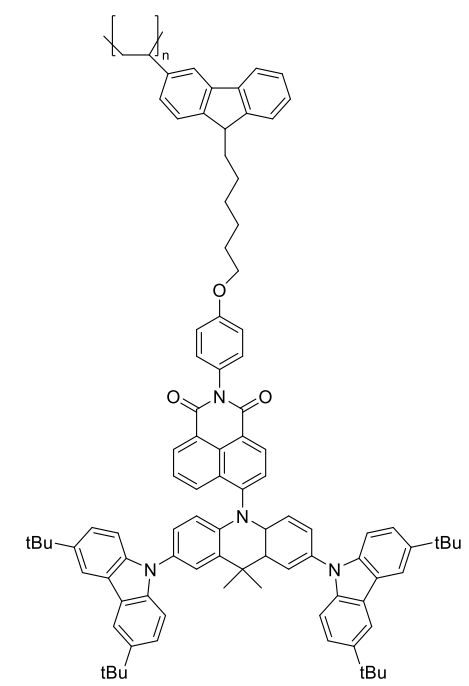 PNAI-AcCz	15.00	1.31	1.44	2.60	659	[28]
	ITO/PEDOT:PSS (40 nm)/PCzAQC0.5 (30 nm)/DPEPO (10 nm)/TmPyPB (50 nm)/Liq (1 nm)/Al (100 nm)	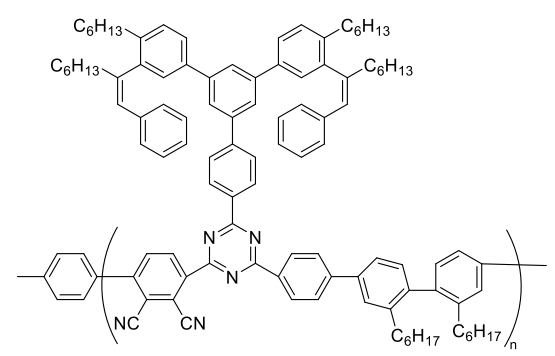 PCzAQC0.5	76.00	-	-	21.20	628	[29]
Complex	ITO/MoO_3 (6 nm)/TAPC (50 nm)/CBP:x% 4FTTPPCuI (20 nm)/TPBi (50 nm)/LiF (1 nm)/Al (100 nm)	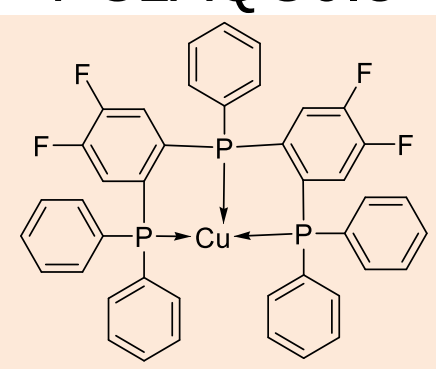 4FTTPPCuI	53.00	20.00	22.00	9.00	603	[30]
	ITO/HAT-CN (5 nm)/TAPC (40 nm)/TCTA (10 nm)/ DMIC-Cz:DMIC-Trz:2 (20 nm)/TPBi (40 nm)/LiF (1 nm)/Al (100 nm)	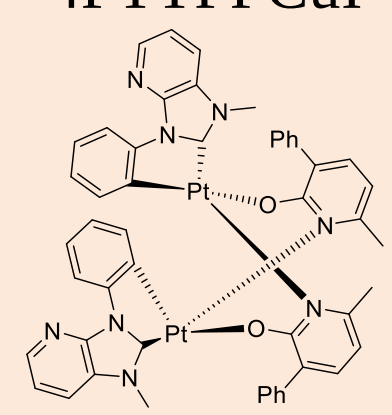 [Pt_2(Ph-NˆO)]	87.00	40.60	51.00	32.70	620	[31]

Cluster	ITO/PEDOT:PSS (40 nm)/PVK (30 nm)/[Au_2Cu_6(Sadm)$_6$(DPPEO)$_6$] NCs (25 nm)/TPBi (50 nm)/Liq (2 nm)/Al (100 nm)	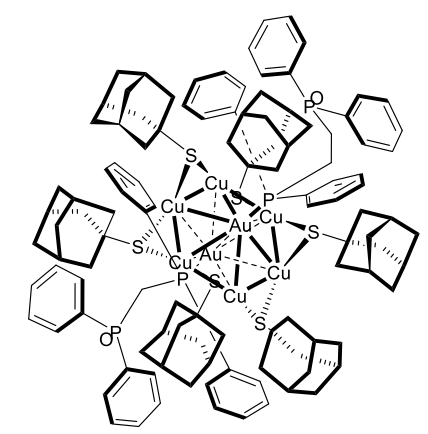 [Au_2Cu_6(Sadm)$_6$(DPPEO)$_6$] NCs	62.00	3.70	3.80	12.60	668	[32]
	ITO/PEDOT:PSS (40 nm)/Poly-TPD (10 nm)/10% R-Au_8:30% TCTA:60% OXD-7 (40 nm)/BmPyPb (40 nm)/LiF (1 nm)/Al (100 nm)	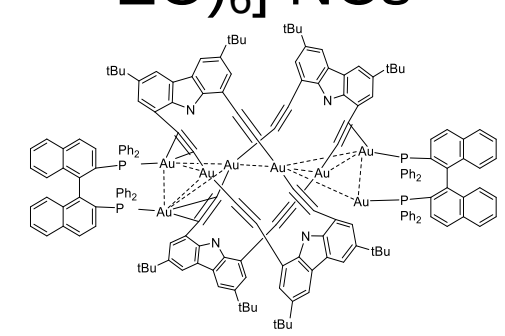 R-Au_8	39.40	-	-	9.90	685	[33]
Quantum Dot (QD)	ITO/PEDOT:PSS (40 nm)/TFB (25 nm)/red QDs-TPP (25 nm)/ZnMgO (35 nm)/Ag (100 nm)	Red Cd-based QDs-TPP	96.10	25.10	-	20.20	630	[34]
	ITO/PEDOT:PSS/TFB (25 nm)/red QDs (25 nm)/ZnMgO (35 nm)/Al (100 nm)	Red CdSe-based QD`s	-	-	-	18.80	630	[35]
	ITO/PEDOT:PSS (38 nm)/TFB (32 nm)/self-assembled InP QDs (17 nm)/ZnMgO (72 nm)/Al (100 nm)	Red InP-based QDs	83.00	-	-	26.60	622	[36]
Perovskite	ITO (150 nm)/PEDOT:PSS (30 nm)/PTAA (20 nm)/PVP (5 nm)/MM-MOPA perovskite (50 nm)/TPBi (40 nm)/LiF (1 nm)/Al (100 nm)	MM-MOPA pure-iodine perovskite	94	-	-	28.70	638	[37]

ITO (150 nm)/PEDOT:PSS (20 nm)/PTAA (20 nm)/$CsPbI_3$ QDs (10 nm)/TmPyPB (6 nm)/PO-T2T (37 nm)/LiF (1 nm)/Al (60 nm)	NSA-NH_4PF_6-treated $CsPbI_3$ perovskite QDs	94	-	-	26.04	628	[38]
ITO/NiO_x (20 nm)/4PACz (1 nm)/TFB (30 nm)/LiF (1.5 nm)/perovskite nanosheet (40 nm)/POT2T (35 nm)/LiF (1.1 nm)/Al (100 nm)	2-NMA-based oriented quasi-2D perovskite nanosheets	91.00	-	-	31.20	635	[39]

[a] Photoluminescence Quantum Yield (PLQY); [b] the maximum efficiencies; [c] EL peak wavelength.

Supplementary References

1. Xie M, Han C, Zhang J, Xie G, Xu H. White Electroluminescent Phosphine-Chelated Copper Iodide Nanoclusters. *Chem Mater* **29**, 6606-6610 (2017).

2. Becke AD. Density‐functional thermochemistry. III. The role of exact exchange. *J Chem Phys* **98**, 5648-5652 (1993).

3. Martin RL. Natural transition orbitals. *J Chem Phys* **118**, 4775-4777 (2003).

4. Lee SY, Yasuda T, Yang YS, Zhang Q, Adachi C. Luminous Butterflies: Efficient Exciton Harvesting by Benzophenone Derivatives for Full-Color Delayed Fluorescence OLEDs. *Angew Chem Int Ed* **53**, 6402-6406 (2014).

5. Tao Y, *et al.* Thermally Activated Delayed Fluorescence Materials Towards the Breakthrough of Organoelectronics. *Adv Mater* **26**, 7931-7958 (2014).

6. Masui K, Nakanotani H, Adachi C. Analysis of exciton annihilation in high-efficiency sky-blue organic light-emitting diodes with thermally activated delayed fluorescence. *Org Electron* **14**, 2721-2726 (2013).

7. Thuy Minh D, *et al.* Tetragold(I) Complexes: Solution Isomerization and Tunable Solid-State Luminescence. *Inorg Chem* **53**, 12720−12731 (2014).

8. Li Y, *et al.* Bulk Passivation Enables Hundredfold-Enhanced Electroluminescence of Monophosphine Cu_4I_4 Cubes. *Angew Chem, Int Ed* **62**, e202308410 (2023).

9. Xu L-J, Zhang X, Wang J-Y, Chen Z-N. High-efficiency solution-processed OLEDs based on cationic Ag_6Cu heteroheptanuclear cluster complexes with aromatic acetylides. *J Mater Chem C* **4**, 1787-1794 (2016).

10. Zeng X-C, Wang J-Y, Xu L-J, Wen H-M, Chen Z-N. Solution-processed OLEDs based on phosphorescent $PtAu_2$ complexes with phenothiazine-functionalized acetylides. *J Mater Chem C* **4**, 6096-6103 (2016).

11. Shu H-X, Wang J-Y, Zhang Q-C, Chen Z-N. Photophysical and Electroluminescent Properties of $PtAg_2$ Acetylide Complexes Supported with meso- and rac-Tetraphosphine. *Inorg Chem* **56**, 9461−9473 (2017).

12. Natarajan N, *et al.* Using phosphorescent $PtAu_3$ clusters for superior solution-processable organic light emitting diodes with very small efficiency roll-off. *J Mater Chem C* **6**, 8966-8976 (2018).

13. Natarajan N, *et al.* $PtAu_3$ cluster complexes with narrow-band emissions for solution-processed organic light emitting diodes. *J Mater Chem C* **7**, 2604-2614 (2019).

14. Xiao H, Zhang X, Wang J-Y, Zhang L-Y, Zhang Q-C, Chen Z-N. Enhancing Phosphorescence through Rigidifying the Conformation to Achieve High-Efficiency OLEDs by Modified PEDOT. *ACS Appl Mater Interfaces* **11**, 45853−45861 (2019).

15. Ohshita J, *et al.* Synthesis of nonplanar bipyridyls bridged by disilane and disiloxane and their phosphorescent copper complexes. *Appl Organometal Chem* **34**, e5306 (2020).

16. Jiao Z, Yang M, Wang J-Y, Huang Y-Z, Xie P, Chen Z-N. High-efficiency solution-processed light-emitting diode based on a phosphorescent Ag_3Cu_5 cluster complex. *J Mater Chem C* **9**, 5528-5534 (2021).

17. Wang Z-Y, *et al.* Solution-Processed Organic Light-Emitting Diodes of Yellow-Emitting $PtAg_2$ Complexes

with an External Quantum Efficiency of 21.7%. *Energy Fuels* **35**, 19132-19138 (2021).

18. Zeng H, Wang J-Y, Shi L-X, Zhang L-Y, Chen Z-N. Platinum(II)-gold(I) heterotrinuclear complexes with N,N '-diarylamine-functionalized acetylide ligands for red electroluminescence. *Inorg Chem Front* **9**, 3156−3164 (2022).

19. Ma X-H, *et al.* Carbene-stabilized enantiopure heterometallic clusters featuring EQE of 20.8% in circularly-polarized OLED. *Nat Commun* **14**, 4121 (2023).

20. Xiao H, Wang J-Y, Zhang L-Y, Shi L-X, Wang Z-Y, Chen Z-N. Naphthalimide-Modified Clusters for Red-Emitting Devices with High Color Purity. *Inorg Chem* **63**, 17157-17165 (2024).

21. Yang X, Cheng H, Shi L-X, Wang J-Y, Chen Z-N. Narrow-band circularly polarized red electroluminescence in trinuclear clusters. *Inorg Chem Front* **11**, 3474-3481 (2024).

22. Ding X-Y, Shi L-X, Wang J-Y, Xu L-J, Zhang L-Y, Chen Z-N. Doping Copper(I) in Ag_7 Cluster for Circularly Polarized OLEDs with External Quantum Efficiency of 26.7 %. *Angew Chem*, *Int Ed* **64**, e202417934 (2025).

23. Jia W-W, *et al.* The Doping Effects on the NIR Emission Enhancement of Gold Nanoclusters and the Application in Efficient NIR-OLED. *Angew Chem, Int Ed* **64**, e202508578 (2025).

24. Cho H, *et al.* Efficient near-infrared organic light-emitting diodes with emission from spin doublet excitons. *Nature Photonics* **18**, 905-912 (2024).

25. Pu Y, Jin Q, Zhang Y, Li C, Duan L, Wang Y. Sulfur-locked multiple resonance emitters for high performance orange-red/deep-red OLEDs. *Nat Commun* **16**, 332 (2025).

26. Pu Y, Cai X, Li C, Liang B, Bi H, Wang Y. Red OLED with efficiency of 25.6% at 10,000 cd m^{-2} based on selenium embedding multiple resonance framework. *Light Sci Appl* **15**, 191 (2026).

27. Minotto A, *et al.* Visible light communication with efficient far-red/near-infrared polymer light-emitting diodes. *Light Sci Appl* **9**, 70 (2020).

28. Li C, *et al.* Deep-red TADF dendronized polymer for efficient non-doped solution-processed OLEDs. *J Mater Chem C* **13**, 15628-15633 (2025).

29. Wang T, *et al.* Effect of molecular weight on photoluminescence and electroluminescence properties of thermally activated delayed fluorescence conjugated polymers. *Chem Eng J* **452**, 139123 (2023).

30. Lou X, *et al.* Efficient Red Electroluminescent Copper Complexes with Fluorination-Balanced Dual Emission. *Research* **9**, (2026).

31. Lo K, *et al.* Stable Binuclear Platinum(II) Emitters with Substituted 2-Hydroxypyridine Bridging Ligands for Red and Near-Infrared OLEDs with EQE up to 32% and LT_{95} up to 26250 h at 1000 cd m^{-2}. *Angew Chem, Int Ed* **65**, e19762 (2025).

32. Rival JV, *et al.* Bimetallic Nanocluster‐Based Light‐Emitting Diodes With High External Quantum Efficiency and Saturated Red Emission. *Adv Mater* **37**, e2507893 (2025).

33. Wei J, *et al.* Topological Engineering From Non-Emissive Chiral Metallomacrocycle to Interlocked Architecture for Strong Circularly Polarized Luminescence. *Angew Chem, Int Ed* **0**, e2560002 (2026).

34. Guan J, *et al.* Direct ambient photopatterning of RGB quantum dots for light-emitting diodes with EQE exceeding 20%. *Nat Commun* **16**, 9689 (2025).

35. Jing Y, *et al.* Photolithographic fabrication of high-resolution Micro-QLEDs towards color-conversion microdisplay. *Light Sci Appl* **14**, 370 (2025).

36. Li H, *et al.* Highly efficient light-emitting diodes via self-assembled InP quantum dots. *Nat Commun* **16**, 4257 (2025).

37. Kong L, *et al.* Fabrication of red-emitting perovskite LEDs by stabilizing their octahedral structure. *Nature* **631**, 73-79 (2024).

38. Li Y, *et al.* Stable and efficient $CsPbI_3$ quantum-dot light-emitting diodes with strong quantum confinement. *Nat Commun* **15**, 5696 (2024).

39. Liu S, *et al.* In-situ formation of oriented perovskite nanosheets with tailored optical dipoles enabling >30% EQE in pure-red LEDs. *Light Sci Appl* **15**, 163 (2026).
