# Supplementary material for: Red electroluminescent Cu4I4 bipyramids with external quantum efficiency beyond 40%
Source: Nat Commun. 2026 Jul 7;17:8380. doi: 10.1038/s41467-026-75381-2 (PMC13473432; doi:10.1038/s41467-026-75381-2)
Supplement: Supplementary file 3 [file 41467_2026_75381_MOESM3_ESM.pdf]

nature portfolio

Peer Review File

Red Electroluminescent Cu4I4 Bipyramids with External Quantum Efficiency beyond 40%

Corresponding Author: Professor Hui Xu

This file contains all reviewer reports in order by version, followed by all author rebuttals in order by version.

Version 0:

Reviewer comments:

Reviewer #1

(Remarks to the Author)

Reviewer #2

(Remarks to the Author)
In this work entitled "Red Electroluminescent Cu4I4 Bipyramids with External Quantum Efficiency beyond 40%", Xu et al. demonstrated high-performance red cluster light-emitting devices based on [PXZDPPQ]2Cu4I4, which should be the best result reported so far to my knowledge. The key excited-state characteristics of [PXZDPPQ]2Cu4I4 were its desired excited-state locations of the outer intraligand charge transfer (nILCT)-featured first singlet (S1) and triplet (T1) excited states and the inner high-lying metal-ligand charge transfer (nMLCT) states, in which the outer and highly radiative nILCT states were spatially and energetically advantageous in carrier capture and exciton confinement, while the inner and high-lying nMLCT states were protected from TPQ effect and supported cross transitions to achieve exactly balanced dual emissions. Consequently, besides a photoluminescence quantum yield of 93.6% and nearly ideal balanced dual emissions with a PH/TADF ratio of 51/49, [PXZDPPQ]2Cu4I4 based devices realized red electroluminescence with λEL of ~620 nm and a state-of-the-art ηEQE as high as 43.7%, which updated the ηEQE record for all kinds of red planar light-emitting devices without additional out-coupling enhancement. This work provided very important and interesting results for cluster material chemistry and optoelectronics, and the paper was well organized. So, it can be published in Nature Communications after minor revisions.

1. According to authors' descriptions, the coordination number would be important for the properties of clusters. So, the differences between "μ2-I" and "μ3-I" should be explained in details. Furthermore, the influences of "triplet-polaron quenching (TPQ)" on electroluminescent performances should be introduced in details.

2. Regarding the red emitting materials, the energy gap rule is commonly considered as the main limitation for luminescence efficiencies. In this work, the authors claimed that the ligand centered excited states can effectively improve the radiative efficiencies, which would be important for cluster emitters. But, the extremely high EQE of the CLEDs far beyond other kinds of planar light-emitting devices can be hardly understood only at the level of molecular excited states. A more detailed explanation on aggregation states and exciton kinetics would be necessary.

3. To make the experimental repeatable, the comprehensive characterization of chemical structures is necessary, such as 1HNMR data, which can be added in supporting information.

4. The authors are suggested to cite more references on dual-emissive copper iodide cluster systems, and high-performance red-emitting devices to clarify the differences or novelty of this work.

5. There are some typos in the manuscript should be revised. For example, the full name of "CLED" is repeated unnecessarily; "constrast" in line 155 should be "contrast"; "functionlization" in line 226 should be "functionalization". In addition, the reference citation format should also be carefully checked and standardized throughout the manuscript.

Reviewer #3

(Remarks to the Author)
Comments:
In this manuscript, Dr. Xu and coworkers report three novel Cu4I4 cluster emitters based on D-A N,P ligands, with particular emphasis on (PXZDPPQ)2Cu4I4. By ligand engineering, the authors aim to regulate the spatial distribution and energetic ordering of different excited states. The optimized compound demonstrates a high PLQY of 93.6% in doped films and enables solution-processed red CLEDs with a reported maximum EQE of 43.7%, which is impressive for planar red electroluminescent devices. The work is potentially significant, as it combines molecular/cluster design, excited-state engineering, and high-performance red electroluminescence, supported by various characterizations and comprehensive analysis. I therefore recommend its publication in Nat. Commun. after following issues are addressed.
Major comments:
1. For the kinetic analysis on page 7 and Fig. 2d, it appears to be organized mainly in terms of PF and DF quantities, while the triplet states seem to contribute only through nonradiative decay according to SI page S18. However, these emitters show dual emissive behavior with substantial phosphorescence contributions, and report PH/TADF ratios of 67/33, 57/43 and 51/49. It is therefore questionable about the rate constants extracted from that analysis. Since PH is not a minor contribution in these systems, the authors are suggested to revise the kinetic model to explicitly include the triplet radiative channel. In addition, the separation of PF, DF and PH is based on fixed time windows of <1 μs, 1-100 μs and >150 μs (SI page S17, lines 186-187). The authors should justify this slicing method and discuss the sensitivity of the extracted rates to the time boundaries.
2. The proposed cascade- and cross-transition mechanisms are interesting and reasonably supported by the TD-DFT/NTO analysis. As I understand it, the essential difference between CZ and PXZ appears to be the spatial localization and energetic ordering of the LCT and MLCT featured excited states, and this spatial and energetic arrangement may indeed help rationalize the different PL/EL behavior and TPQ tendency. The authors should clarify whether the improved performance is primarily attributed to this spatial/energetic arrangement or to a specific kinetic pathway. If the latter is emphasized, additional evidence for the merit of cross-transitions would be needed, since both the proposed cascade and cross pathways involve multiple IC, ISC and RISC processes, and the observed lifetimes appear to be comparable.
Minor comments:
3. The reported maximum EQE of 43.7% for the PXZ CLED is very impressive. To better support the reliability of this value, the authors are recommended to provide reproducibility data, such as average and maximum EQE values, standard deviations or error bars. Such statistical information would make the device performance claim more convincing.
4. Since the manuscript discusses the potential of these emitters for display applications, preliminary operational stability data of the optimized red CLEDs would be useful.
5. Several structure–property statements should be clarified or toned down. For example, the “remote steric effects” of CZ/PXZ (page 4, line 125) appear speculative. The phrase “doubles dihedral angles of coordination geometry” (page 5, line 146) is also unclear, please specify which dihedral angles are meant, provide the numerical values, and explain how they support enhanced rigidity.
6. More generally, terms such as “less radiative nMLCT states and highly radiative nLCT states” (page 6, lines 168-169), should be used more cautiously or supported by quantitative evidence/references, these expressions imply general photophysical advantages that are not self-evident from the MLCT/LCT assignments alone. Similarly, the term “ideal PH/TADF ratio” (page 8, line 249) requires clarification. The manuscript appears to imply that a balanced PH/TADF ratio is beneficial, but it is not clearly explained why a nearly 1:1 PH/TADF contribution should be advantageous, especially compared with a TADF-dominant system.

Version 1:

Reviewer comments:

Reviewer #1

(Remarks to the Author)
I carefully read the authors' responses to my comments and see that the authors did a good job on manuscript revision. Therefore, I would like to recommend the publication of this excellent work.

Reviewer #2

(Remarks to the Author)
As all the concerned issues raised in the first round of review have been well addressed and responded, the work could be published.

Reviewer #3

(Remarks to the Author)
The authors have satisfactorily addressed the concerns raised by the reviewers. I therefore recommend acceptance of this work for publication.

Open Access This Peer Review File is licensed under a Creative Commons Attribution 4.0 International License, which permits use, sharing, adaptation, distribution and reproduction in any medium or format, as long as you give appropriate credit to the original author(s) and the source, provide a link to the Creative Commons license, and indicate if changes were made.
In cases where reviewers are anonymous, credit should be given to 'Anonymous Referee' and the source.
The images or other third party material in this Peer Review File are included in the article's Creative Commons license, unless indicated otherwise in a credit line to the material. If material is not included in the article's Creative Commons license and your intended use is not permitted by statutory regulation or exceeds the permitted use, you will need to obtain permission directly from the copyright holder.
To view a copy of this license, visit https://creativecommons.org/licenses/by/4.0/

Responses to Reviewers' Comments

Reviewer #2:

General comment: In this work entitled "Red Electroluminescent Cu_4I_4 Bipyramids with External Quantum Efficiency beyond 40%", Xu et al. demonstrated high-performance red cluster light-emitting devices based on $[PXZDPPQ]_2Cu_4I_4$, which should be the best result reported so far to my knowledge. The key excited-state characteristics of $[PXZDPPQ]_2Cu_4I_4$ were its desired excited-state locations of the outer intraligand charge transfer (^{n}ILCT)-featured first singlet (S_1) and triplet (T_1) excited states and the inner high-lying metal-ligand charge transfer (^{n}MLCT) states, in which the outer and highly radiative ^{n}ILCT states were spatially and energetically advantageous in carrier capture and exciton confinement, while the inner and high-lying ^{n}MLCT states were protected from TPQ effect and supported cross transitions to achieve exactly balanced dual emissions. Consequently, besides a photoluminescence quantum yield of 93.6% and nearly ideal balanced dual emissions with a PH/TADF ratio of 51/49, $[PXZDPPQ]_2Cu_4I_4$ based devices realized red electroluminescence with λ_{EL} of ~620 nm and a state-of-the-art η_{EQE} as high as 43.7%, which updated the η_{EQE} record for all kinds of red planar light-emitting devices without additional out-coupling enhancement. This work provided very important and interesting results for cluster material chemistry and optoelectronics, and the paper was well organized. So, it can be published in Nature Communications after minor revisions.

Response: Reviewer's kind approval and accurate summary of our work is highly appreciated. Thanks a lot!

1. According to authors' descriptions, the coordination number would be important for the properties of clusters. So, the differences between "μ^2-I" and "μ^3-I" should be explained in details. Furthermore, the influences of "triplet-polaron quenching (TPQ)" on electroluminescent performances should be introduced in details.

Response: Thanks a lot for this constructive comment! We should apologize for our insufficient explanation on coordination optimization and TPQ effect on electroluminescent

(EL) performances. We would like to response one by on as following:

(i) The coordination number variation of iodine atoms significantly influences the photophysical properties of the clusters.

As reviewer pointed out, the coordination modes of iodide bridges would influence the structural and photophysical properties of copper iodide clusters. The "μ^2-I" and "μ^3-I" are distinguished, according to the different numbers of coordination bonds for these two kinds of iodine atoms, namely "2" and "3" (*Pure Appl. Chem.* **1999**, *71(8)*, 1557-1585; *Eur. J. Inorg. Chem.* **2013**, *2013(2),* 187-202; *Cryst. Eng. Comm.* **2014**, *16*, 5338-5344). As consequence, not only the Cu-I bond lengths but also Cu-Cu distances and core geometries can be modulated through changing the coordination numbers of iodine atoms, which directly influences metal-metal and metal-counter ion interactions and ligand-metallic core charge transfer, therefore changing photoluminescent properties of the clusters, including emission wavelengths and quantum yields (*Cryst. Eng. Comm.* **2014**, *16(24)*: 5338-5344; *New J Chem.* **2023**, *47(42)*: 19751-19759; *J Solid State Chem.* **2024**, *333:* 124639).

In this work, the Cu_4I_4 cores of $[CzDPPQ]_2Cu_4I_4$ and $[PXZDPPQ]_2Cu_4I_4$ adopt bipyramidal configurations including Cu_4 rhomboids, where two μ^2-I atoms and two μ^3-I atoms are respectively located on opposite sides of the Cu_4 plane, and on the top and bottom of the Cu_4 plane. Compared with our previously reported octahedral Cu_4I_4 analogue (*Nat. Commun.* **2024**, *15(1)*: 4997), Cu-Cu distances on the four sides of the Cu_4 rhomboids in $[CzDPPQ]_2Cu_4I_4$ and $[PXZDPPQ]_2Cu_4I_4$ increase by approximately 0.1 Å, whereas the distances between two diagonal μ^3-I-bridged Cu^+ ions are markedly shortened by approximately 1.0 Å, giving rise to additional Cu-Cu interactions for more uniform Cu···Cu distributions and connections in Cu_4I_4 cores. Obviously, compared to μ^2-I atoms, μ^3-I atoms not only stabilize and fix Cu_4 rhomboids, but also strengthen intra-core electronic coupling and enhance M^nLCT interactions, leading to remarkable emission bathochrmic shift. Particularly, in the case of $[PXZDPPQ]_2Cu_4I_4$, the combination of its bipyramidal Cu_4I_4 and strong electron-donating phenoxazine (PXZ)-modified PXZDPPQ ligand enables the excited-state evolution from CC/MLCT domination to highly radiative LCT-featured S_1 and T_1 states. As a result, besides further emission red-shifting, non-radiative transitions are effectively suppressed, accompanied by the balanced TADF/PH dual emission, giving rise to

markedly improved photoluminescence and electroluminescence quantum efficiencies up to 93.6% and 43.7%, respectively.

(ii) Triplet-polaron quenching (TPQ) in cluster light-emitting diodes can be alleviated by accelerating radiation and averting charge-exciton interactions.

As reviewer pointed out, the influence of TPQ effect on EL performances of the clusters is important, and should be introduced and discussed in details. TPQ is a nonradiative process in which triplet excitons are quenched by interactions with polarons during the EL process, corresponding to nonradiative deactivation of triplet states to ground states (*Org. Electron.* **2021**, *91*, 106058). Therefore, since TPQ consumes the triplet excitons already formed in the emissive layers, it directly reduces the exciton utilization ratio and thereby EL quantum efficiencies. Furthermore, since the densities of both triplet excitons and polarons are in proportion to current densities, the triplet-polaron interactions are also enhanced along with current density increasing, consequently aggravating TPQ and worsening efficiency roll-off at high luminance (*Chem. Sci.* **2016**, *7(4):* 2870-2882; *Physical Review B* **2017**, *96,* 184203). This process becomes more pronounced at high current density because both triplet exciton density and charge-carrier density increase during device operation. As a result, TPQ deactivates triplet excitons through a non-radiative pathway, reduces the number of radiative excitons, and consequently lowers exciton utilization efficiency and accelerates EQE roll-off.

The previous studies on EL efficiency decreases at high brightness showed the predominance of TPQ in quenching effects for phosphorescence and TADF OLEDs, whose proportions can exceed 50% (*Adv. Mater.* **2013**, *25(47)*: 6801-6827; *Appl. Phys. Lett.* **2010**, *97(24)*, 243304; *Appl. Phys. Lett.* **2014**, *105(14)*: 143303; *Nat. Commun.* **2022**, *13(1)*: 254). In our previous reports of host materials for TADF OLEDs, the exciton and charge transfer channels on host molecules were separated to reduce exciton-polaron interactions and suppress TPQ (*Adv. Mater.* **2014**, *26*, 7070-7077). In this work, TPQ in the CLED was closely related to the spatial and energetic distributions of the excited states for the clusters, since the high-lying and peripheral excited states suffer from more serious TPQ, while high-lying embedded excited states can be protected by peripheral radiative low-lying excited states. For $[CzDPPQ]_2Cu_4I_4$, its outer and high-lying LCT states are exposed to polarons and predominant in carrier recombination before energy transfer to the inner MLCT-featured S_1

and T_1 states. This excited-state arrangement increases exciton-polaron interactions and delays radiation. In contrast, for $[PXZDPPQ]_2Cu_4I_4$, its lowest and highly radiative LCT-featured S_1 and T_1 states are located on the outer ligands, which facilitates direct carrier recombination and prompt exciton radiation. Meanwhile, its inner high-lying MLCT states are embedded in the coordination skeleton and encapsulated by the outer LCT states, thus preventing direct interactions with polarons and suppressing TPQ. This spatially separated and energetically optimized excited-state distribution is beneficial for reducing non-radiative exciton loss and improving EL quantum efficiency, giving rise to the ultrahigh EQE beyond 40%.

The explanations and discussions were added in the related sections. Thanks a lot!

Revision:

Discussions on the coordination numbers of iodine atoms and triplet-polaron quenching (TPQ) were added:

"Due to synergistically exothermic electron transfer and internal conversion, high-lying LC states of red clusters more easily capture charges and electro-generated excitons to increase the probability of charge-exciton interactions and worsen triplet-polaron quenching (TPQ), which is commonly in direct proportion to current and exciton densities.[18]"

"Through bridging with μ^3-I atoms, distances between two μ^3-Cu^+ ions at diagonal lines of Cu_4 rhomboids are markedly decreased by ~1.0 Å, giving rise to additional Cu-Cu bonds and more uniform Cu-Cu interactions in whole metallic cores. Consequently, the combination of μ^2-I and μ^3-I bridges strengthen the connectivity and interactions within Cu_4I_4 cores.[52 53]"

"On the contrary, outer and high-lying ^{n}LCT states of $[CzDPPQ]_2Cu_4I_4$ are non-radiative, but predominant in carrier recombination and the subsequent energy transfer to its inner and ^{n}MLCT-featured S_1 and T_1 states, leading to its delayed radiation and more pronounced TPQ."

"18. Ligthart A, Nevels TDG, Weijtens CHL, Bobbert PA, Coehoorn R. Mechanistic description of the efficiency loss in organic phosphorescent host–guest systems due to triplet-polaron quenching. *Org Electron* **91**, 106058 (2021)."

"52. Müller C, Broeckx LEE, de Krom I, Weemers JJM. Developments in the Coordination Chemistry of Phosphinines. *Eur J Inorg Chem* **2013**, 187-202 (2013).

53. Wei F, Liu X, Liu Z, Bian Z, Zhao Y, Huang C. Structural and photophysical study of copper iodide complex with P^N or P^N^P ligand. *CrystEngComm* **16**, 5338-5344 (2014). "

2. Regarding the red emitting materials, the energy gap rule is commonly considered as the main limitation for luminescence efficiencies. In this work, the authors claimed that the ligand centered excited states can effectively improve the radiative efficiencies, which would be important for cluster emitters. But, the extremely high EQE of the CLEDs far beyond other kinds of planar light-emitting devices can be hardly understood only at the level of molecular excited states. A more detailed explanation on aggregation states and exciton kinetics would be necessary.

Response: Thanks a lot for this constructive comment! The energy gap law was originally established for radiation-less transitions in large molecules by Englman and Jortner, and it is commonly used to explain the enhanced non-radiative decay of red and near-infrared emitters *(Mol. Phys.* **1970**, *18(2)*: 145-164). We agree with reviewer's opinion that although the excited state optimization established the basis to overcome energy gap law for achieving the state-of-the-art PLQY and the maximum EQE of $[PXZDPPQ]_2Cu_4I_4$ beyond 90% and 40%, the record-high EQE of the $[PXZDPPQ]_2Cu_4I_4$-based CLEDs markedly exceeds the theoretical EQE up-limit of 35% for planar light-emitting devices using ITO substrates.

EQE can be given by:

$$\eta_{\mathrm{EQE}} = \gamma \times \eta_{\mathrm{EUE}} \times \Phi_{\mathrm{PL}} \times \eta_{\mathrm{out}}$$

in which the charge balance factor γ, exciton utilization efficiency η_{EUE}, photoluminescence quantum yield Φ_{PL} and outcoupling efficiency η_{out} determine the EQE values. Compared to 25% for fluorescent emitters, the maximum η_{EUE} can be 100% for thermally activated delayed fluorescence (TADF) and phosphorescent emitters. In this work, since all the clusters are TADF-phosphorescence dual emission systems, their theoretical η_{EUE} can reach 100%. Furthermore, through device engineering, the carrier injection and transportation can be balanced to unify the carrier recombination, namely $\gamma = 1$. Significantly, in planar OLEDs, EQE should be further modified by considering η_{out} as functions of the horizontal dipole

orientation ratio Θ and device geometry Γ, because the emitting dipole orientation and microcavity effect strongly affect light extraction (*Adv. Funct. Mater.* **2013**, *23(31)*: 3896-3900).

In this work, Φ_{PL} values of the clusters are improved mainly by molecular excited-state optimization and host-dopant energy transfer. CBP:15% $[PXZDPPQ]_2Cu_4I_4$ film exhibits a high Φ_{PL} of 93.6%, which is higher than that of CBP:30% $[CzDPPQ]_2Cu_4I_4$ and more than eight times that of $[DPPQ]_2Cu_4I_4$-based films. This result demonstrates that the LCT-featured S_1/T_1 states and dispersion in CBP matrix effectively facilitate radiative transitions and suppress non-radiative decay and concentration quenching. Taking η_{EQE} of 43.7% and Φ_{PL} of 93.6% and assuming $\gamma = 1$ and $\eta_{EUE} = 100\%$, η_{out} should reach 46.7%, larger than 30-35% for ITO based devices.

Angle-dependent emission spectra of the films were measured to figure out the aggregation states of cluster dopants in CBP matrixes. The CBP:30% $[CzDPPQ]_2Cu_4I_4$ and CBP:15% $[PXZDPPQ]_2Cu_4I_4$ films show horizontal dipole orientation ratios Θ of 75.5% and 76.6%, respectively, both higher than the 66.7% for isotropic orientation (Supplementary Fig. 27). Although it is commonly more difficult to determine the accurate horizontal dipole orientation ratio for spin-coated films, the higher Θ values of CBP:30% $[CzDPPQ]_2Cu_4I_4$ can reflect the more enhanced light extraction at forward direction of the devices, which undoubtedly contributes to the extremely high EQE of its devices.

We further measured the angular electroluminescence distributions of the optimal devices (*Opt. Express* **2008**, *16*: 1808-1819; *Adv. Mater.* **2018**, *30(35)*: 1802285). The angular profiles of EL intensities can be fitted with:

$$I(\theta) = I(0)\cos^n \theta$$

CBP:30% $[CzDPPQ]_2Cu_4I_4$ and CBP:15% $[PXZDPPQ]_2Cu_4I_4$ devices give n values of 0.93 and 0.71, respectively (Fig. 4e). Compared with the Lambertian reference (n = 1), both devices were in accord with forward-dominant super-Lambertian emission, but the EL emission of CBP:15% $[PXZDPPQ]_2Cu_4I_4$ based devices is more concentrated at forward direction, leading to higher light extraction efficiency and the EQE beyond 40%.

Actually, regarding to η_{EUE}, the exciton kinetics investigation shows that $[PXZDPPQ]_2Cu_4I_4$ is superior in radiation improvement and quenching suppression, since its

lowest and highly radiative LCT-featured S_1 and T_1 states are located on the outer ligands, which is beneficial for direct carrier recombination and prompt radiation. Meanwhile, its inner high-lying MLCT states are embedded in the coordination skeleton and protected from polaron-induced quenching, especially TPQ. This excited-state distribution simultaneously facilitates cross transitions between MLCT and LCT states. In contrast, the outer high-lying LCT states of $[CzDPPQ]_2Cu_4I_4$ are exposed to intermolecular interactions, and should transfer energy to the inner MLCT-featured S_1/T_1 states in a cascade mode, leading to delayed radiation and more serious TPQ. So, despite their comparable PLQYs more than 90% and comparable electrical properties, $[PXZDPPQ]_2Cu_4I_4$ endowed its device with the record high EQE of 43.7%, which is about one fifth higher than 35.3% for $[CzDPPQ]_2Cu_4I_4$-based devices. At 100 nits, the EQE value of $[PXZDPPQ]_2Cu_4I_4$-based devices still remains as high as 35.3%, in contrast to 25.0% for $[CzDPPQ]_2Cu_4I_4$-based analogs.

Therefore, the record-high EQE of the $[PXZDPPQ]_2Cu_4I_4$-based CLED should be a combined result of (i) high Φ_{PL} realized by its optimized excited state characteristics, e.g. its LCT-predominant S_1 and T_1 states and suppressed non-radiative decays; (ii) desired aggregation behaviors in high horizontal dipole orientation and super-Lambertian angular emission, leading to large out-coupling ratio; and (iii) balanced TADF/PH dual emissions and suppressed TPQ, markedly limiting efficiency roll-offs.

The detailed explanations and discussions were added in the related sections. Thanks a lot!

Revision:

Discussions on the determinants for ultrahigh EQE were added:

"In addition, the angular electroluminescence distributions of $[PXZDPPQ]_2Cu_4I_4$ and $[CzDPPQ]_2Cu_4I_4$ based devices were in accord with forward-dominant super-Lambertian emission (**Figure 4e**), which was consistent with the increased horizontal dipole orientation ratios of the corresponding films (**Supplementary Fig. 36**). Nonetheless, the forward out-coupling proportion of CBP:15% $[PXZDPPQ]_2Cu_4I_4$ was markedly larger than that of CBP:30% $[CzDPPQ]_2Cu_4I_4$, reflecting the higher out-coupling ratio of the former. On account of nearly the same η_{PL} values and comparable electrical properties for $[CzDPPQ]_2Cu_4I_4$ and

[PXZDPPQ]$_2$Cu$_4$I$_4$ doped films, one fifth larger η_{EQE} of [PXZDPPQ]$_2$Cu$_4$I$_4$ based devices should be attributed to the combined superiorities in exciton utilization and light extraction."

3. To make the experimental repeatable, the comprehensive characterization of chemical structures is necessary, such as ^{1}H NMR data, which can be added in supporting information.

Response: Thanks a lot for this constructive comment! The ^{1}H NMR spectra were added in the experimental section of Supplementary Information. Thanks a lot!

Revision:

<u>^{1}H NMR spectra were added:</u>

"As a proof of concept, we used quinoline instead of pyridine in DPPy to construct the coordination unit DPPQ with larger conjugation (**Supplementary Fig. 1-7**)."

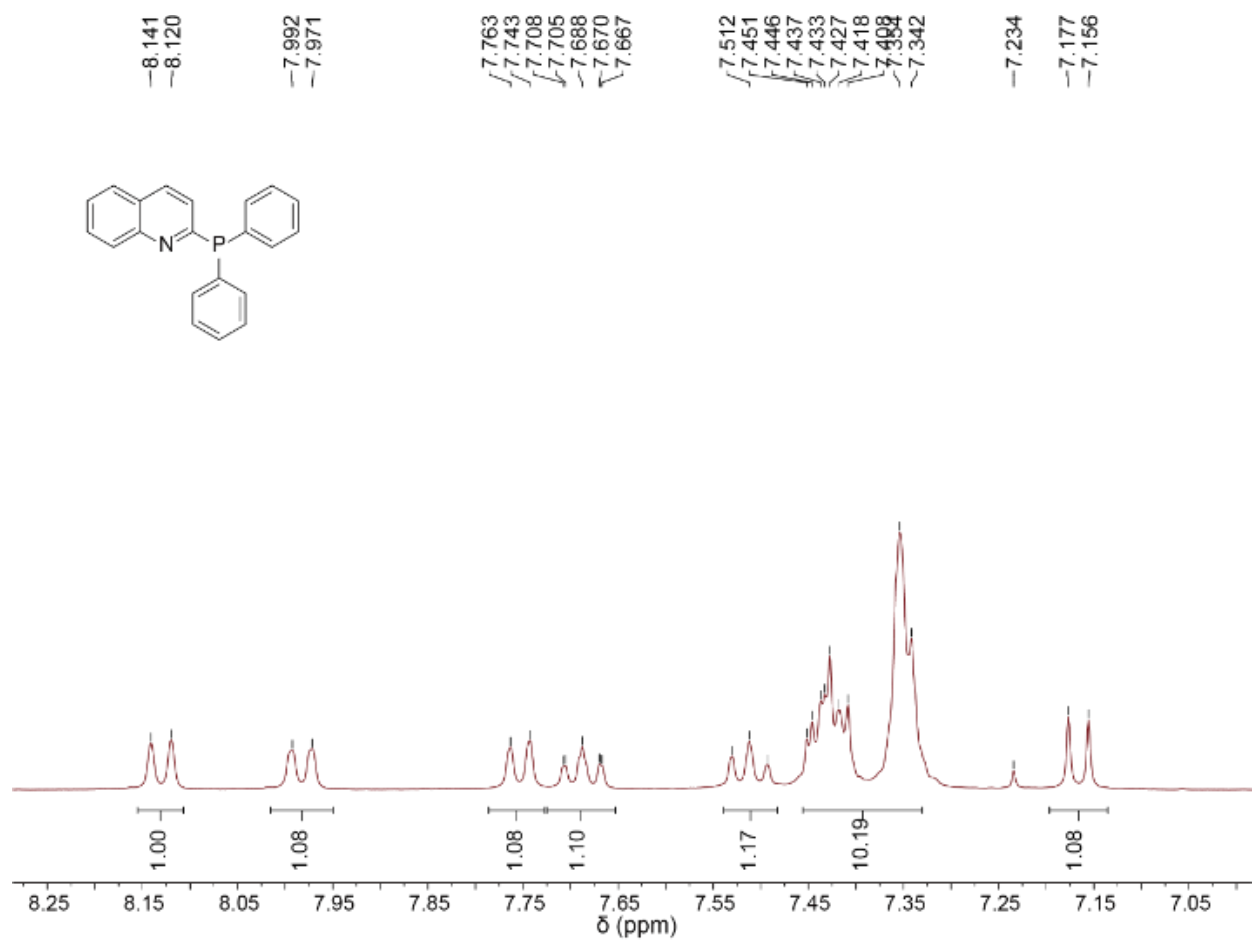

Supplementary Fig. 2 | ^{1}H NMR spectrum of DPPQ in $CDCl_3$.

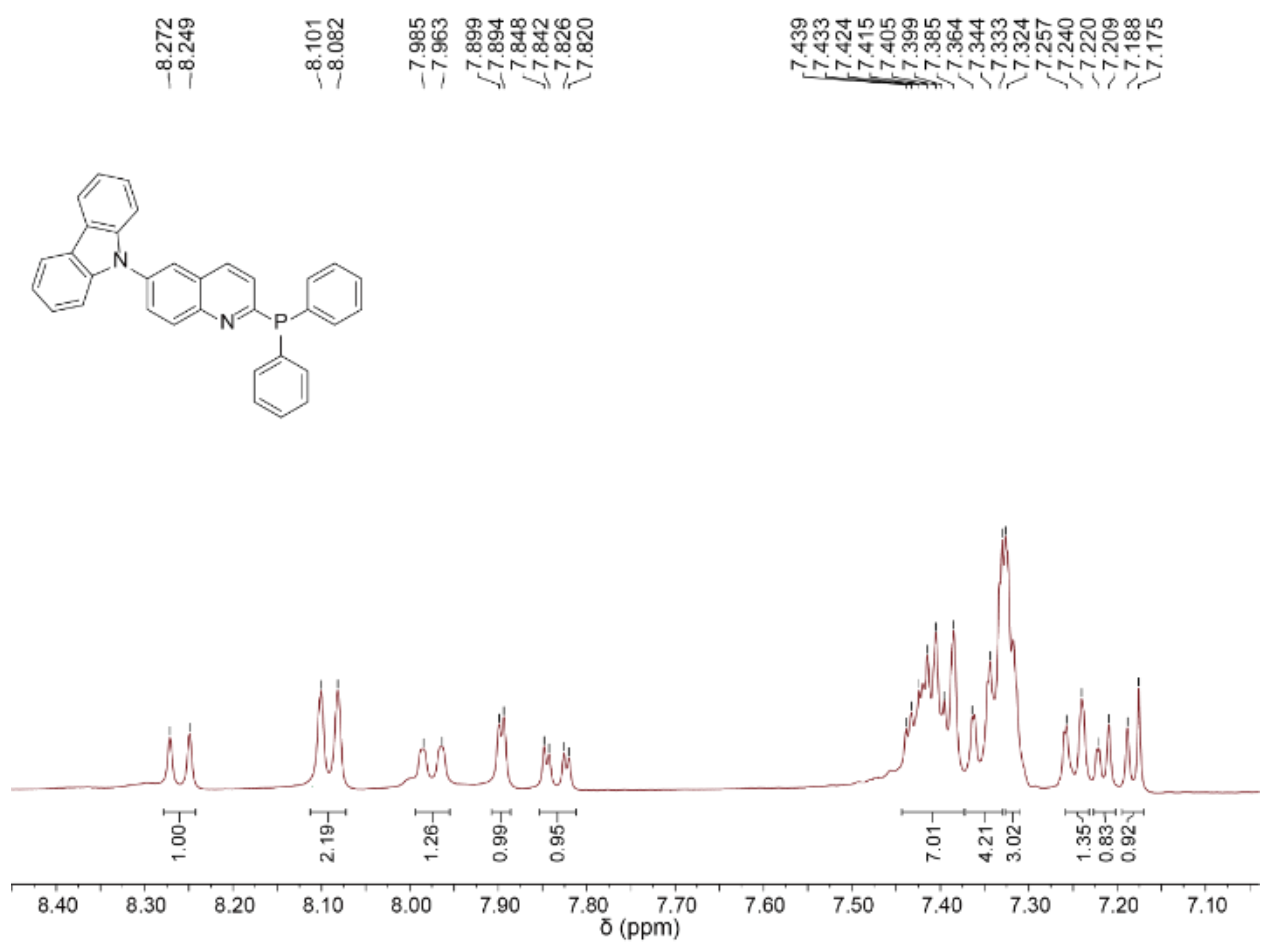

Supplementary Fig. 3 | ^{1}H NMR spectrum of CzDPPQ in $CDCl_3$.

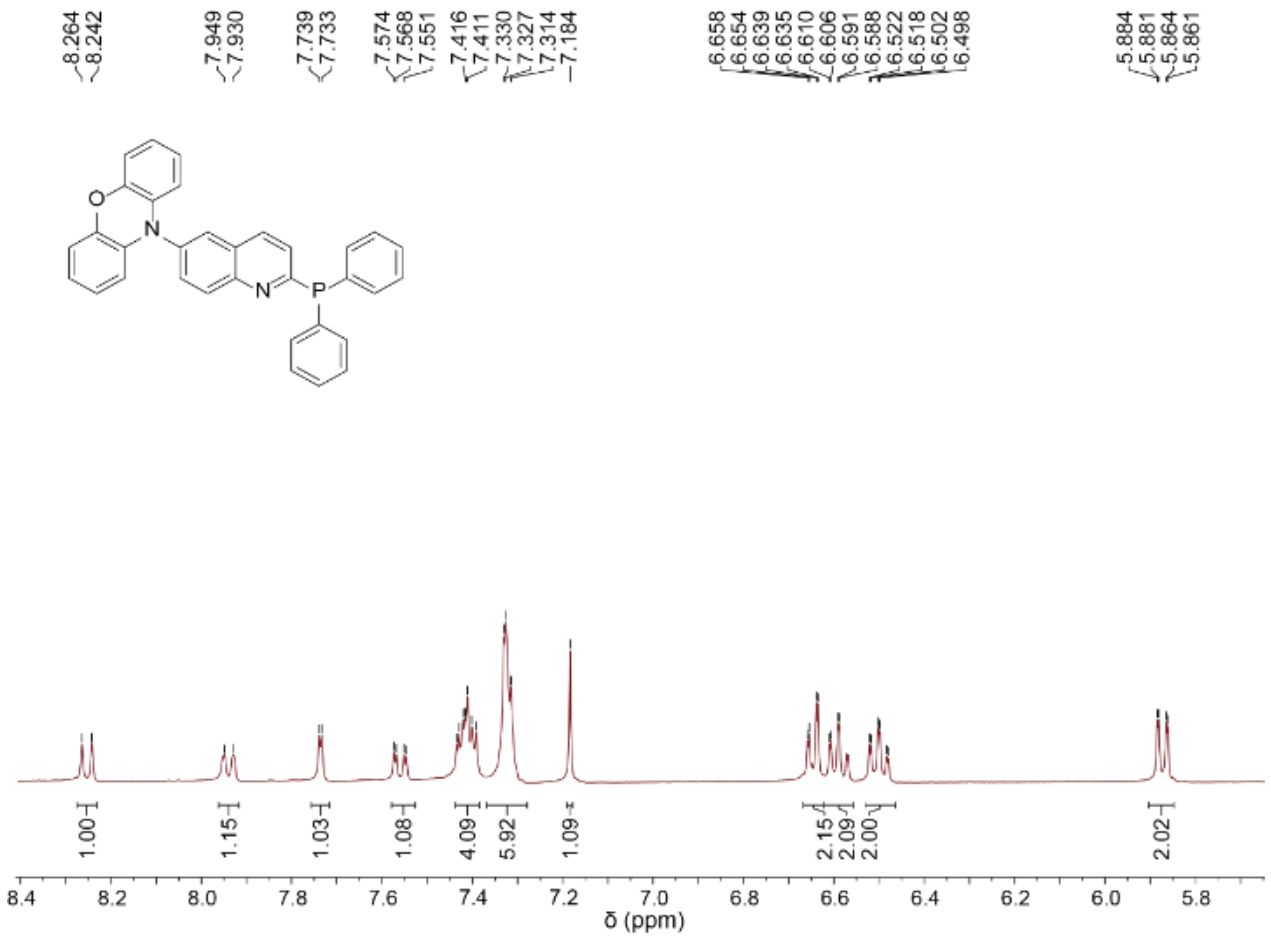

Supplementary Fig. 4 | ^{1}H NMR spectrum of PXZDPPQ in $CDCl_3$.

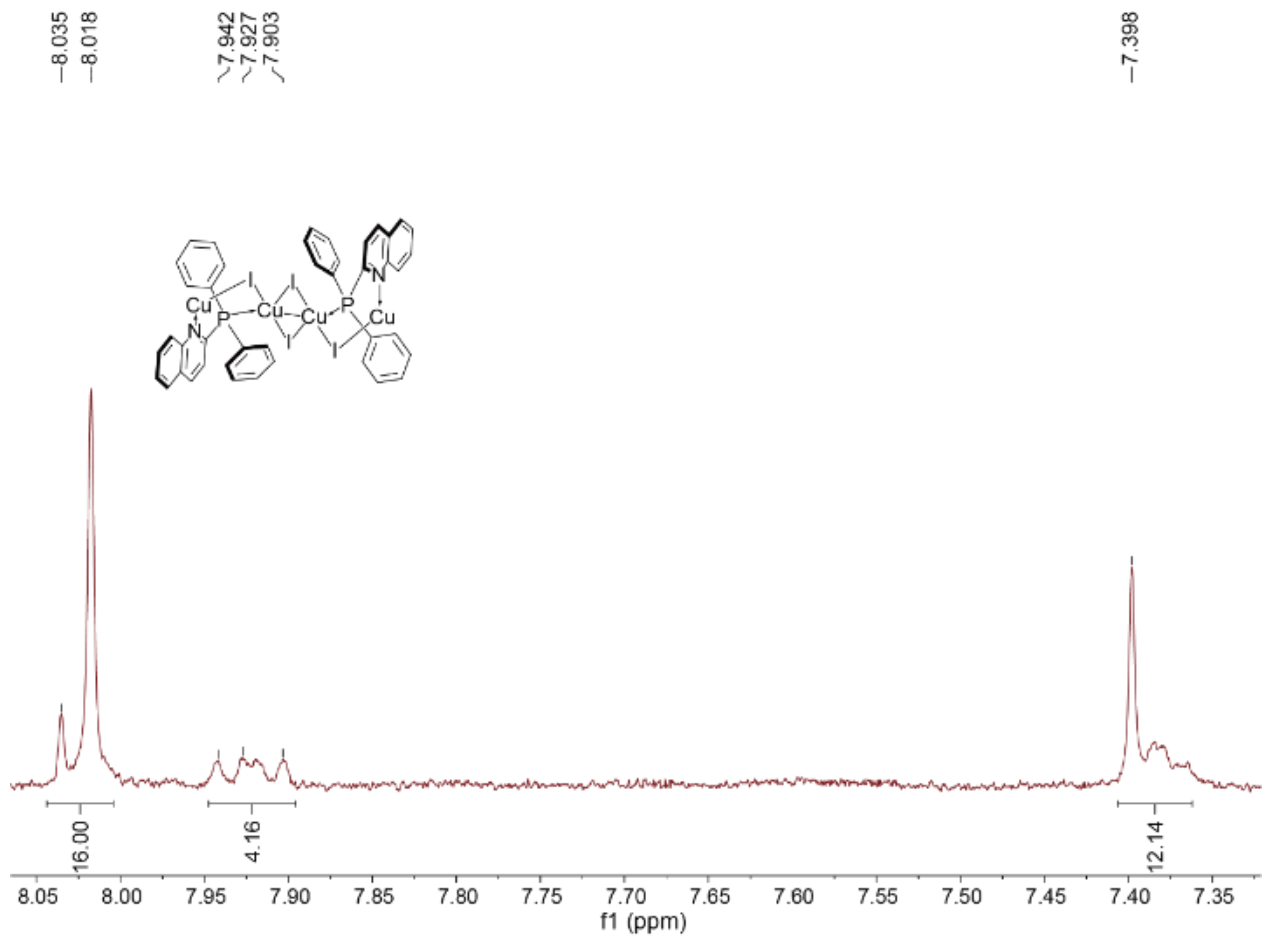

Supplementary Fig. 5 | ^{1}H NMR spectrum of $[DPPQ]_2Cu_4I_4$ in $CDCl_3$.

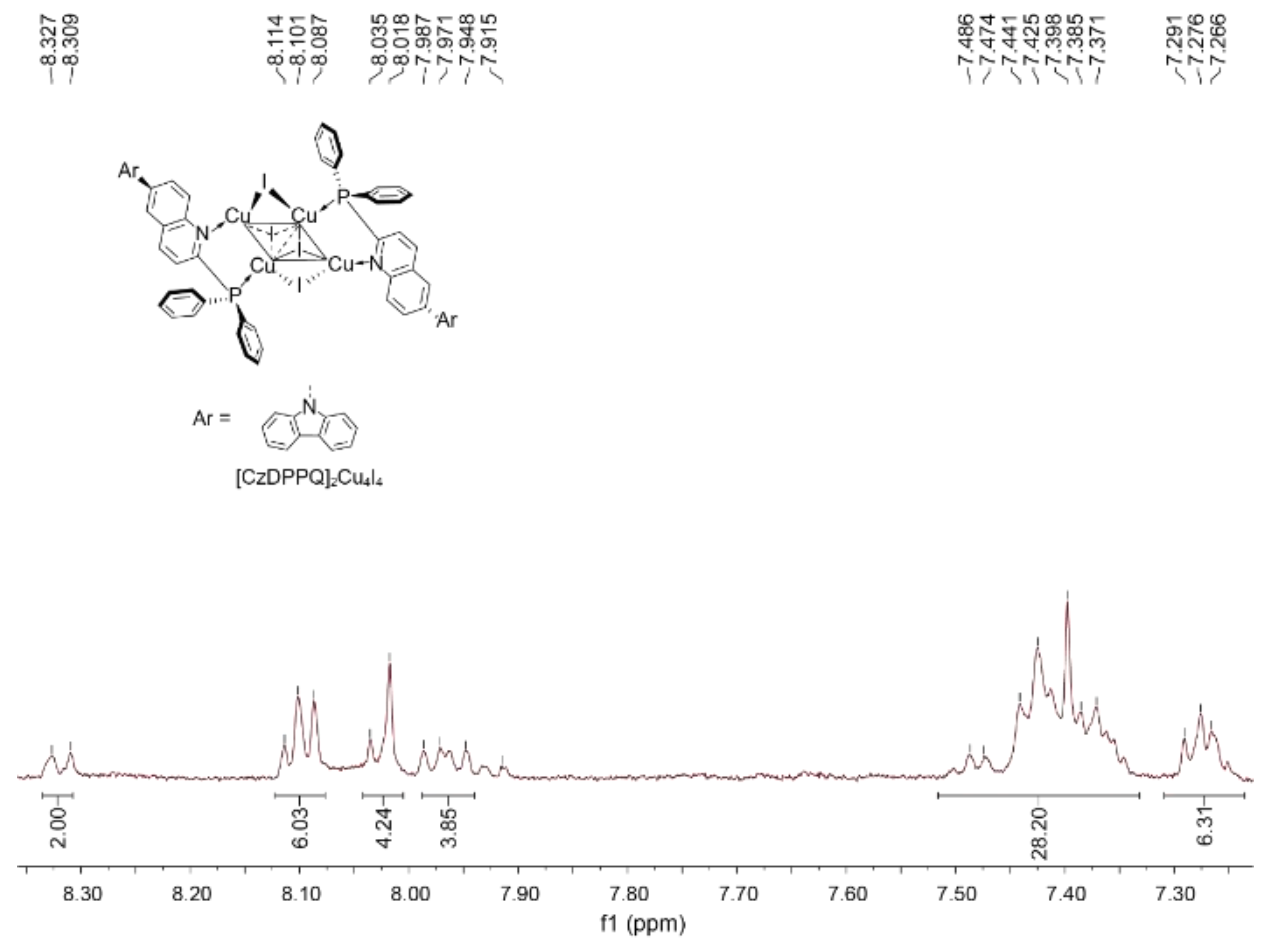

Supplementary Fig. 6 | ^{1}H NMR spectrum of $[CzDPPQ]_2Cu_4I_4$ in $CDCl_3$.

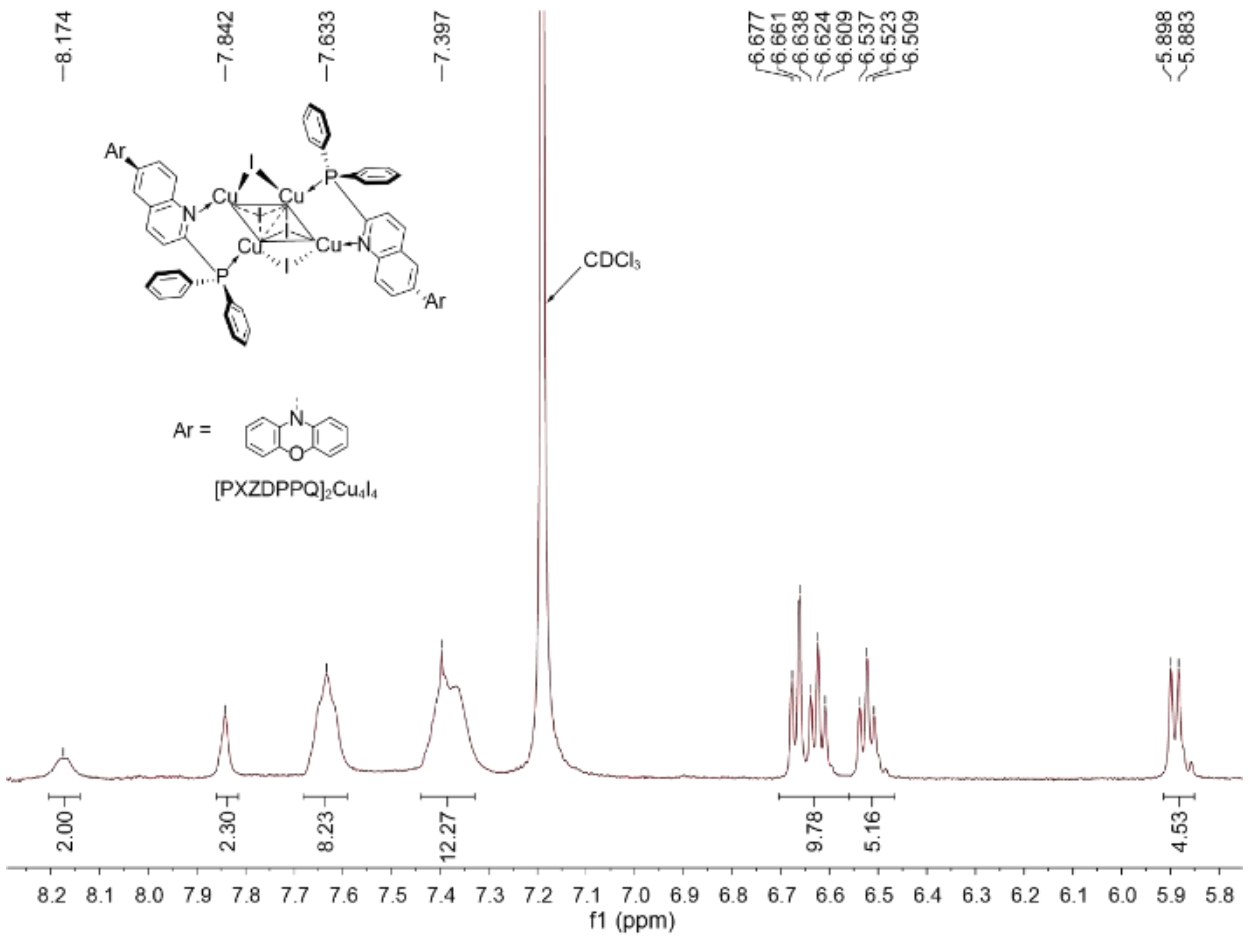

Supplementary Fig. 7 | ^{1}H NMR spectrum of $[PXZDPPQ]_2Cu_4I_4$ in $CDCl_3$.

4. The authors are suggested to cite more references on dual-emissive copper iodide cluster systems, and high-performance red-emitting devices to clarify the differences or novelty of this work.

Response: Thanks a lot for this constructive comment! We should apologize for our insufficient citations. Accordingly, six works about dual-emissive copper iodide clusters were cited. The related device performances were also added in Supplementary Table 5.

To demonstrate the superiorities of $[PXZDPPQ]_2Cu_4I_4$ in electroluminescent performances with respect to red-emitting materials, the representative systems for red light-emitting diodes were listed in Supplementary Table 6 for comparison, including red-emitting small molecules featuring thermally activated delayed fluorescence (TADF) and/or multi-resonance, polymers, complexes, clusters, quantum dots and perovskites.

It indicates that compare to all the other kinds of electroluminescent materials, $[PXZDPPQ]_2Cu_4I_4$ significantly improves the external quantum efficiencies by about one third to undoubtedly realize the record-high device efficiencies, which can be ascribed to: (i) the superiority of organic-inorganic clusters in accurate excited-state optimization through modulating metal-metal interactions in the cores, donor-acceptor interactions in the ligands and ligand-core interactions, which can control the compositions and characteristics of the lowest excited states, e.g. the LCT-predominant S_1 and T_1 states and balanced dual emissions of $[PXZDPPQ]_2Cu_4I_4$, giving rise to high photoluminescence quantum yields; (ii) adjustable electrical properties and carrier recombination channels of the clusters to separate charge and

exciton for TPQ suppression and roll-off limitation; (iii) the tunable molecular geometries by controllable assembly for desired aggregation behaviors in high horizontal dipole orientation and super-Lambertian angular emission, leading to large out-coupling ratios.

The related paragraph was revised, and the data were added in Supplementary Table 5 and 6. Thanks a lot!

Revision:

Comparison on performances of representative red-emitting systems and dual-emissive copper iodide clusters was added:

"Actually, moderate SOC endows copper iodide emitters[30-32] with both thermally activated delayed fluorescence (TADF) and phosphorescence (PH) from their first singlet (S_1) and triplet (T_1) states.[33-36] This establishes the basis for constructing dual emissive materials with suitable TADF/PH ratios.[37-42]"

"Actually, compared to other kinds of red electroluminescent materials (**Supplementary Table 6**), the merit of cluster emitters is fully embodied by $[PXZDPPQ]_2Cu_4I_4$ in synergy and complementarity between multiple excited states involved in exciton formation and utilization."

"37. Hofbeck T, Monkowius U, Yersin H. Highly Efficient Luminescence of Cu(I) Compounds: Thermally Activated Delayed Fluorescence Combined with Short-Lived Phosphorescence. *J Am Chem Soc* **137**, 399-404 (2015).

38. Baranov AY, *et al.* New Cu(i) halide complexes showing TADF combined with room temperature phosphorescence: the balance tuned by halogens. *Dalton Trans* **49**, 3155-3163 (2020).

39. Sun C, *et al.* Nuclearity Control for Efficient Thermally Activated Delayed Fluorescence in a CuI Complex and its Halogen-Bridged Dimer. *Chem Mater* **33**, 6383-6393 (2021).

40. Chatterjee J, *et al.* Activation of TADF in Photon Upconverting Crystals of Dinuclear Cu(I)-Iodide Complexes by Ligand Engineering. *J Phys Chem Lett* **15**, 6069-6080 (2024).

41. Lin S, *et al.* Efficient non-doped cluster light-emitting diodes based on

semiconducting copper iodide hybrids. *J Lumin* **271**, 120609 (2024).

42. Zhang D, *et al.* Efficient and bright broadband electroluminescence based on environment-friendly metal halide nanoclusters. *Light Sci Appl* **13**, 82 (2024)."

Supplementary Table 6 | Comparison on performances of recently reported representative red-emitting emitters.

Material Type	Device Structure	Emitter	η_{PL}[a] (%)	η[b]		η_{EQE} (%)	λ_{EL}[c] (nm)	Ref.
				η_{CE} (cd A^{-1})	η_{PE} (lm W^{-1})			
Radical	ITO/MoO_3 (3 nm)/TAPC (30 nm)/TCTA (10 nm)/MADN:TTM-TPA 3 wt% (20 nm)/B3PYMPM (80 nm)/LiF (1 nm)/Al (100 nm)	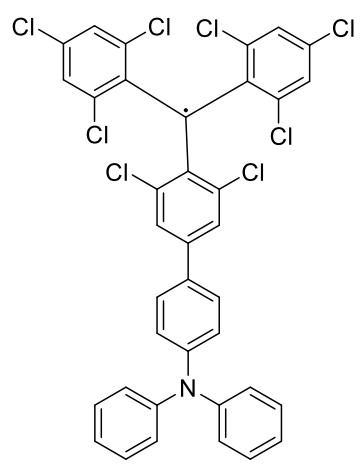 TTM-TPA	27.00	-	-	9.60	800	23
Multi-Resonance	ITO/TAPC (50 nm)/TCTA (10 nm)/DMIC-TRZ:0.5 wt% 2S-BN (30 nm)/TmPyPb (30 nm)/LiF (1 nm)/Al (100 nm)	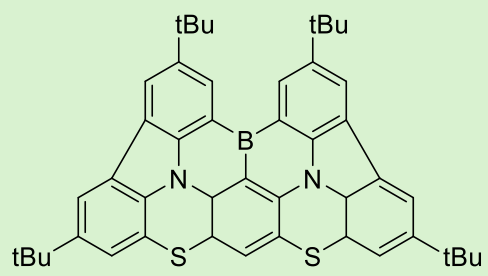 2S-BN	98.40	4.20	4.40	29.30	676	24
	ITO/TAPC (50 nm)/TCTA (10 nm)/DMIC-TRZ:1 wt% tFSBN (30 nm)/TmPyPB (35 nm)/LiF (1 nm)/Al (100 nm)	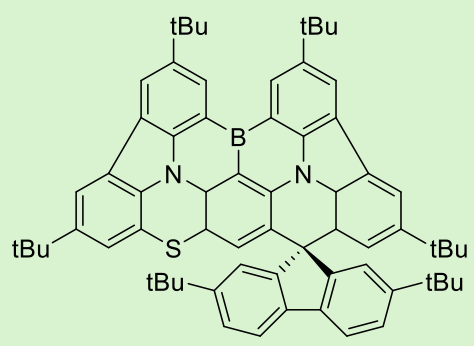 tFSBN	98.00	41.90	48.70	35.20	620	25
Thermally Activated Delayed Fluorescence (TADF) Emitter	ITO/PEDOT:PSS (40 nm)/F8BT:0.2 wt% eDPP (125 nm)/Ca (30 nm)/Al (200 nm)	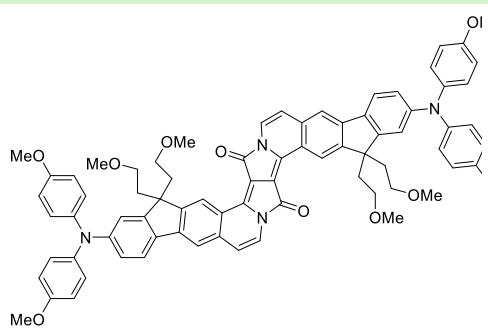 eDPP	33.00	-	-	2.72	670	26

	ITO/PEDOT:PSS (40 nm)/PNAI-AcCz (40 nm)/TmPyPB (42 nm)/LiF (1 nm)/Al (100 nm)	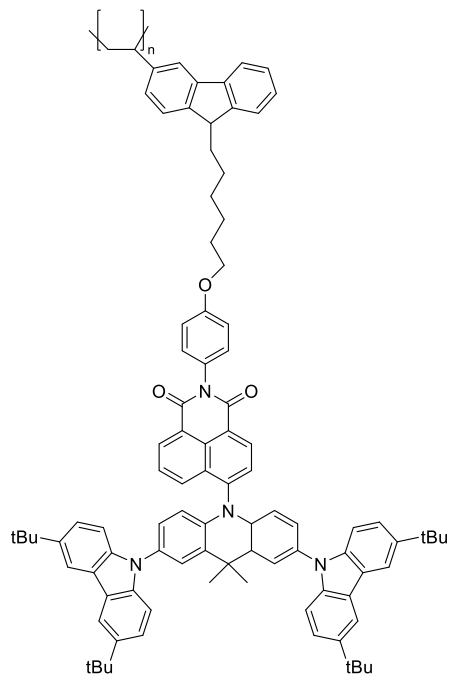 PNAI-AcCz	15.00	1.31	1.44	2.60	659	27
	ITO/PEDOT:PSS (40 nm)/PCzAQC0.5 (30 nm)/DPEPO (10 nm)/TmPyPB (50 nm)/Liq (1 nm)/Al (100 nm)	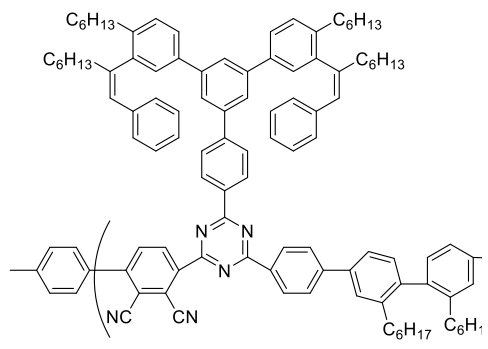 PCzAQC0.5	76.00	-	-	21.20	628	28
Complex	ITO/MoO_3 (6 nm)/TAPC (50 nm)/CBP:x% 4FTTPPCuI (20 nm)/TPBi (50 nm)/LiF (1 nm)/Al (100 nm)	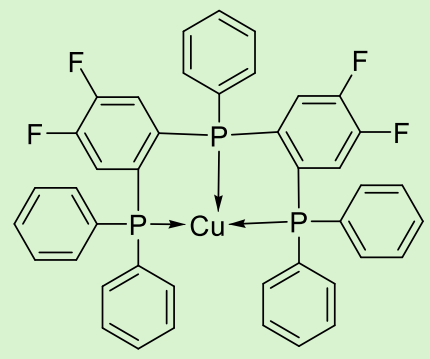 4FTTPPCuI	53.00	20.00	22.00	9.00	603	29
	ITO/HAT-CN (5 nm)/TAPC (40 nm)/TCTA (10 nm)/ DMIC-Cz:DMIC-Trz :2 (20 nm)/TPBi (40 nm)/LiF (1 nm)/Al (100 nm)	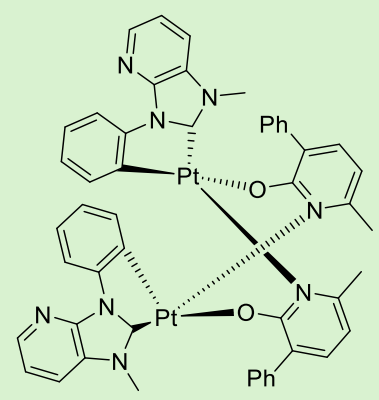 [Pt_2(Ph-N^O)]	87.00	40.60	51.00	32.70	620	30
Cluster	ITO/PEDOT:PSS (40 nm)/PVK (30 nm)/$[Au_2Cu_6(Sadm)_6(DPPEO)_6]$ NCs (25 nm)/TPBi (50 nm)/Liq (2 nm)/Al (100 nm)	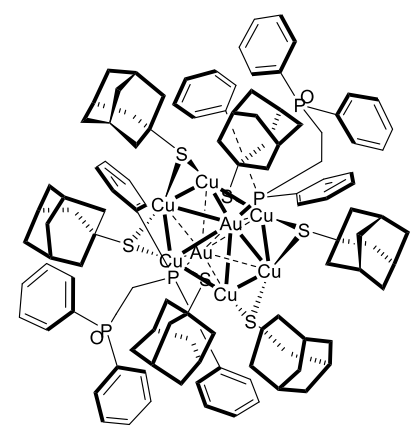 $[Au_2Cu_6(Sadm)_6(DPPEO)_6]$ NCs	62.00	3.70	3.80	12.60	668	31
	ITO/PEDOT:PSS (40 nm)/Poly-TPD (10 nm)/10% R-Au_8:30% TCTA:60% OXD-7 (40 nm)/BmPyPb (40	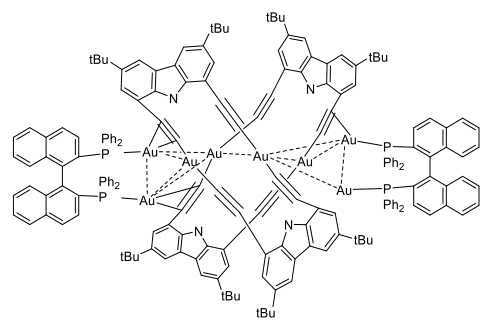	39.40	-	-	9.90	685	32

	nm)/LiF (1 nm)/Al (100 nm)	R-Au_8						
Quantum Dot (QD)	ITO/PEDOT:PSS (40 nm)/TFB (25 nm)/red QDs-TPP (25 nm)/ZnMgO (35 nm)/Ag (100 nm)	Red Cd-based QDs-TPP	96.10	25.10	-	20.20	630	33
	ITO/PEDOT:PSS/TFB (25 nm)/red QDs (25 nm)/ZnMgO (35 nm)/Al (100 nm)	Red CdSe-based QD`s	-	-	-	18.80	630	34
	ITO/PEDOT:PSS (38 nm)/TFB (32 nm)/self-assembled InP QDs (17 nm)/ZnMgO (72 nm)/Al (100 nm)	Red InP-based QDs	83.00	-	-	26.60	622	35
Perovskite	ITO (150 nm)/PEDOT:PSS (30 nm)/PTAA (20 nm)/PVP (5 nm)/MM-MOPA perovskite (50 nm)/TPBi (40 nm)/LiF (1 nm)/Al (100 nm)	MM-MOPA pure-iodine perovskite	94	-	-	28.70	638	36
	ITO (150 nm)/PEDOT:PSS (20 nm)/PTAA (20 nm)/$CsPbI_3$ QDs (10 nm)/TmPyPB (6 nm)/PO-T2T (37 nm)/LiF (1 nm)/Al (60 nm)	NSA-NH_4PF_6-treated $CsPbI_3$ perovskite QDs	94	-	-	26.04	628	37
	ITO/NiO_x (20 nm)/4PACz (1 nm)/TFB (30 nm)/LiF (1.5 nm)/perovskite nanosheet (40 nm)/POT2T (35 nm)/LiF (1.1 nm)/Al (100 nm)	2-NMA-based oriented quasi-2D perovskite nanosheets	91.00	-	-	31.20	635	38

[a] Photoluminescence Quantum Yield (PLQY); [b] the maximum efficiencies; [c] EL peak

wavelength.

5. There are some typos in the manuscript should be revised. For example, the full name of "CLED" is repeated unnecessarily; "constrast" in line 155 should be "contrast"; "functionlization" in line 226 should be "functionalization". In addition, the reference citation format should also be carefully checked and standardized throughout the manuscript.

Response: Thanks a lot for this constructive comment! We should apologize for our carelessness during manuscript preparation. The manuscript was rechecked carefully to try our best to get rid of typos, grammar errors and format errors. Thanks a lot!

Revision:

Typos and the reference formats were corrected:

"Recently emerged cluster light-emitting diodes (CLED) …"

"In contrast to Cz-dispersed HOMO-6 ..."

"… donor functionalization comprehensively ..."

"1. Zhang XJ, Xu H. Electroluminescent Clusters. *Angew Chem, Int Ed* **63**, e202317597 (2024).

2. Zhong C, Zhang X, Gong Z, Xu H. Recent Advances in Electroluminescent Metallic Nanoclusters: From Materials to Devices. *Nano Lett* **24**, 9415-9428 (2024).

3. Shirasaki Y, Supran GJ, Bawendi MG, Bulović V. Emergence of colloidal quantum-dot light-emitting technologies. *Nat Photonics* **7**, 13-23 (2012).

4. Fakharuddin A*, et al.* Perovskite light-emitting diodes. *Nat Electron* **5**, 203-216 (2022).

5. Matsushima t*, et al.* High performance from extraordinarily thick organic light-emitting diodes. *Nature* **572**, 502-506 (2019).

6. Gong S, Yang C, Qin J. Efficient phosphorescent polymer light-emitting diodes by suppressing triplet energy back transfer. *Chem Soc Rev* **41**, 4797-4807 (2012).

7. Kim T*, et al.* Efficient and stable blue quantum dot light-emitting diode. *Nature* **586**, 385-389 (2020).

8. Cao Y*, et al.* Perovskite light-emitting diodes based on spontaneously formed

submicrometre-scale structures. *Nature* **562**, 249-253 (2018).

9. Luo J*, et al.* Efficient and stable emission of warm-white light from lead-free halide double perovskites. *Nature* **563**, 541-545 (2018).

10. Uoyama H, Goushi K, Shizu K, Nomura H, Adachi C. Highly efficient organic light-emitting diodes from delayed fluorescence. *Nature* **492**, 234-238 (2012).

11. Liu Y, Li C, Ren Z, Yan S, Bryce MR. All-organic thermally activated delayed fluorescence materials for organic light-emitting diodes. *Nat Rev Mater* **3**, 18020 (2018).

12. Wang ZY*, et al.* Flexible light-emitting diodes with EQE approaching 26.4% from horizontal dipole-oriented metal nanoclusters. *Nat Commun* **17**, 767 (2026).

13. Reineke S*, et al.* White organic light-emitting diodes with fluorescent tube efficiency. *Nature* **459**, 234-238 (2009).

14. McCarthy MA*, et al.* Low-Voltage, Low-Power, Organic Light-Emitting Transistors for Active Matrix Displays. *Science* **332**, 570-573 (2011).

15. Xu H*, et al.* Recent progress in metal-organic complexes for optoelectronic applications. *Chem Soc Rev* **43**, 3259-3302 (2014).

16. Tang R*, et al.* Highly stable and efficient copper(I) sensitizer for narrowband red organic light-emitting diodes with an operational lifetime (LT_{95}) of up to 3689 h at 1000 cd m^{-2}. *Nat Commun* **16**, 7776 (2025).

17. Ford PC, Cariati E, Bourassa J. Photoluminescence Properties of Multinuclear Copper(I) Compounds. *Chem Rev* **99**, 3625-3648 (1999).

18. Ligthart A, Nevels TDG, Weijtens CHL, Bobbert PA, Coehoorn R. Mechanistic description of the efficiency loss in organic phosphorescent host–guest systems due to triplet-polaron quenching. *Org Electron* **91**, 106058 (2021).

19. Niesen B, Rand BP. Thin Film Metal Nanocluster Light-Emitting Devices. *Adv Mater* **26**, 1446-1449 (2014).

20. Ding XY*, et al.* Cooperative Construction of Silver(I) Nanoclusters of Primary-Tertiary Diphosphine Directed by Thiolate Templates Through Ligand Metathesis. *Aggregate* **6**, e70173 (2025).

21. Jia W-W*, et al.* The Doping Effects on the NIR Emission Enhancement of Gold Nanoclusters and the Application in Efficient NIR-OLED. *Angew Chem, Int Ed* **64**,

e202508578 (2025).

22. Zeng XC, Wang JY, Xu L-J, Wen H-M, Chen Z-N. Solution-processed OLEDs based on phosphorescent $PtAu_2$ complexes with phenothiazine-functionalized acetylides. *J Mater Chem C* **4**, 6096-6103 (2016).

23. Natarajan N*, et al.* PtAu3 cluster complexes with narrow-band emissions for solution-processed organic light emitting diodes. *J Mater Chem C* **7**, 2604-2614 (2019).

24. Xu LJ, Zhang X, Wang J-Y, Chen Z-N. High-efficiency solution-processed OLEDs based on cationic Ag_6Cu heteroheptanuclear cluster complexes with aromatic acetylides. *J Mater Chem C* **4**, 1787-1794 (2016).

25. Rival JV*, et al.* Bimetallic Nanocluster-Based Light-Emitting Diodes With High External Quantum Efficiency and Saturated Red Emission. *Adv Mater* **37**, 2507893 (2025).

26. Ding XY, Shi LX, Wang JY, Xu L-J, Zhang L-Y, Chen Z-N. Doping Copper(I) in Ag_7 Cluster for Circularly Polarized OLEDs with External Quantum Efficiency of 26.7 %. *Angew Chem, Int Ed* **64**, e202417934 (2025).

27. Olaru M*, et al.* A Small Cationic Organo-Copper Cluster as Thermally Robust Highly Photo- and Electroluminescent Material. *J Am Chem Soc* **142**, 373-381 (2020).

28. Lin YY*, et al.* Lantern-like Cu_8 cluster for high-efficiency electrically driven light-emitting diodes. *Dalton Trans* **54**, 13483-13491 (2025).

29. Ma X-H*, et al.* Carbene-stabilized enantiopure heterometallic clusters featuring EQE of 20.8% in circularly-polarized OLED. *Nat Commun* **14**, 4121-4132 (2023).

30. Hamze R*, et al.* Eliminating nonradiative decay in Cu(I) emitters: >99% quantum efficiency and microsecond lifetime. *Science* **363**, 601-606 (2019).

31. Ford PC, Vogler A. Photochemical and photophysical properties of tetranuclear and hexanuclear clusters of metals with d10 and s2 electronic configurations. *Acc Chem Res* **26**, 220-226 (1993).

32. Wang J-J*, et al.* High efficiency warm-white light-emitting diodes based on copper–iodide clusters. *Nat Photonics* **18**, 200-206 (2024).

33. Czerwieniec R, Leitl MJ, Homeier HHH, Yersin H. Cu(I) complexes-Thermally activated delayed fluorescence. Photophysical approach and material design. *Coord Chem Rev* **325**, 2-28 (2016).

34. Zink DM*, et al.* Heteroleptic, Dinuclear Copper(I) Complexes for Application in Organic Light-Emitting Diodes. *Chem Mater* **25**, 4471-4486 (2013).

35. Zhang QS, Komino T, Huang S, Matsunami S, Goushi K, Adachi C. Triplet Exciton Confinement in Green Organic Light-Emitting Diodes Containing Luminescent Charge-Transfer Cu(I) Complexes. *Adv Funct Mater* **22**, 2327-2336 (2012).

36. Li XY*, et al.* Bluish-Green Cu(I) Dimers Chelated with Thiophene Ring-Introduced Diphosphine Ligands for Both Singlet and Triplet Harvesting in OLEDs. *ACS Appl Mater Interfaces* **11**, 3262-3270 (2019).

37. Hofbeck T, Monkowius U, Yersin H. Highly Efficient Luminescence of Cu(I) Compounds: Thermally Activated Delayed Fluorescence Combined with Short-Lived Phosphorescence. *J Am Chem Soc* **137**, 399-404 (2015).

38. Baranov AY*, et al.* New Cu(i) halide complexes showing TADF combined with room temperature phosphorescence: the balance tuned by halogens. *Dalton Trans* **49**, 3155-3163 (2020).

39. Sun C*, et al.* Nuclearity Control for Efficient Thermally Activated Delayed Fluorescence in a CuI Complex and its Halogen-Bridged Dimer. *Chem Mater* **33**, 6383-6393 (2021).

40. Chatterjee J*, et al.* Activation of TADF in Photon Upconverting Crystals of Dinuclear Cu(I)-Iodide Complexes by Ligand Engineering. *J Phys Chem Lett* **15**, 6069-6080 (2024).

41. Lin S*, et al.* Efficient non-doped cluster light-emitting diodes based on semiconducting copper iodide hybrids. *J Lumin* **271**, 120609 (2024).

42. Zhang D*, et al.* Efficient and bright broadband electroluminescence based on environment-friendly metal halide nanoclusters. *Light Sci Appl* **13**, 82 (2024).

43. Martin A*, et al.* Mechano-Stimulus and Environment-Dependent Circularly Polarized TADF in Chiral Copper(I) Complexes and Their Application in OLEDs. *J Am Chem Soc* **145**, 4438–4449 (2023).

44. Zhang Q*, et al.* Harnessing of Cooperative Cu···H Interactions for Luminescent Low-Coordinate Copper(I) Complexes towards Stable OLEDs. *Angew Chem, Int Ed* **64**, e202419290 (2024).

45. Niu J*, et al.* Equalized Dual Emissions from Copper Complexes via Multichannel Balanced Intersystem Crossing: Toward 100% Quantum Efficiencies. *Angew Chem Int Ed* **64**,

e202508667 (2025).

46. Xie M, Han C, Zhang J, Xie G, Xu H. White Electroluminescent Phosphine-Chelated Copper Iodide Nanoclusters. *Chem Mater* **29**, 6606-6610 (2017).

47. Zhang L, Li B. Room-Temperature Pure Blue-Emitting Phosphorescent Multinuclear Cu(I)-Based Emitters. *J Electrochem Soc* **156**, J174-J178 (2009).

48. Lu X, *et al.* Efficient doped and non-doped light-emitting diodes based on a TADF-emitting Cu4Br4 cluster. *Inorg Chem Front* **11**, 2775-2783 (2024).

49. Bizzarri C, Spuling E, Knoll DM, Volz D, Bräse S. Sustainable metal complexes for organic light-emitting diodes (OLEDs). *Coord Chem Rev* **373**, 49-82 (2018).

50. Liu Y, Yiu S-C, Ho C-L, Wong W-Y. Recent advances in copper complexes for electrical/light energy conversion. *Coord Chem Rev* **375**, 514-557 (2018).

51. Zhang Q-C, Xiao H, Zhang X, Xu L-J, Chen Z-N. Luminescent oligonuclear metal complexes and the use in organic light-emitting diodes. *Coord Chem Rev* **378**, 121-133 (2019).

52. Xie M, Han C, Liang Q, Zhang J, Xie G, Xu H. Highly efficient sky blue electroluminescence from ligand-activated copper iodide clusters: Overcoming the limitations of cluster light-emitting diodes. *Sci Adv* **5**, eaav9857. (2019).

53. Zhang N, *et al.* Overcoming Efficiency Limitation of Cluster Light-Emitting Diodes with Asymmetrically Functionalized Biphosphine Cu4I4 Cubes. *J Am Chem Soc* **144**, 6551-6557 (2022).

54. Sun JN, *et al.* Ligand-mediate exciton allocation enables efficient cluster-based white light-emitting diodes via single and heavy doping. *Nat Commun* **15**, 4997 (2024).

55. Zhang N, *et al.* Sky Blue and Yellow Cluster Light-Emitting Diodes Based on Asymmetric Cu_4I_4 Nanocubes. *Research* **2022**, 0005 (2022).

56. Meng YS, *et al.* Excited state reversal in copper iodide clusters enables 100% exciton radiation. *Nat Commun* **17**, 933 (2025).

57. Hung WY, Wang TC, Chiang PY, Peng BJ, Wong KT. Remote Steric Effect as a Facile Strategy for Improving the Efficiency of Exciplex-Based OLEDs. *ACS Appl Mater Interf* **9**, 7355-7361 (2017).

58. Müller C, Broeckx LEE, de Krom I, Weemers JJM. Developments in the Coordination

Chemistry of Phosphinines. *Eur J Inorg Chem* **2013**, 187-202 (2013).

59. Wei F, Liu X, Liu Z, Bian Z, Zhao Y, Huang C. Structural and photophysical study of copper iodide complex with P^N or P^N^P ligand. *CrystEngComm* **16**, 5338-5344 (2014).

60. Perruchas S, *et al.* Mechanochromic and Thermochromic Luminescence of a Copper Iodide Cluster. *J Am Chem Soc* **132**, 10967-10969 (2010).

61. Ma Y, *et al.* Allochroic cluster light-emitting diodes based on unique μ_3-tetraphosphine Cu_3X_3 crowns with tunable excited states. *Sci Adv* **10**, eadk3983 (2024).

62. Lu J, *et al.* High-Efficiency Circularly Polarized Light-Emitting Diodes Based on Chiral Metal Nanoclusters. *J Am Chem Soc* **146**, 4144-4152 (2024).

63. Ge L, *et al.* Efficient and Stable Narrowband Pure-Red Light-Emitting Diodes with Electroluminescence Efficiencies Exceeding 43%. *J Am Chem Soc* **146**, 32826-32836 (2024)."

Reviewer #3:

General comment: In this manuscript, Dr. Xu and coworkers report three novel Cu_4I_4 cluster emitters based on D-A N,P ligands, with particular emphasis on $[PXZDPPQ]_2Cu_4I_4$. By ligand engineering, the authors aim to regulate the spatial distribution and energetic ordering of different excited states. The optimized compound demonstrates a high PLQY of 93.6% in doped films and enables solution-processed red CLEDs with a reported maximum EQE of 43.7%, which is impressive for planar red electroluminescent devices. The work is potentially significant, as it combines molecular/cluster design, excited-state engineering, and high-performance red electroluminescence, supported by various characterizations and comprehensive analysis. I therefore recommend its publication in Nat. Commun. after following issues are addressed.

Response: Reviewer's approval and accurate brief summary on our work are highly appreciated! Thanks a lot!

Major comments:

1. For the kinetic analysis on page 7 and Fig. 2d, it appears to be organized mainly in terms of PF and DF quantities, while the triplet states seem to contribute only through nonradiative decay according to SI page S18. However, these emitters show dual emissive behavior with substantial phosphorescence contributions, and report PH/TADF ratios of 67/33, 57/43 and 51/49. It is therefore questionable about the rate constants extracted from that analysis. Since PH is not a minor contribution in these systems, the authors are suggested to revise the kinetic model to explicitly include the triplet radiative channel. In addition, the separation of PF, DF and PH is based on fixed time windows of <1 μs, 1-100 μs and >150 μs (SI page S17, lines 186-187). The authors should justify this slicing method and discuss the sensitivity of the extracted rates to the time boundaries.

Response: Thanks a lot for this constructive comment! We appreciate and agree with reviewer's insightful suggestion. Considering the dual-emission features of the clusters, it is more rational to simultaneously evaluate their TADF and PH properties. Actually, since the first triplet (T_1) state is simultaneously involved in multiple radiative and non-radiative transitions, it is difficult to accurately evaluate TADF and PH transition characteristics at the

same time. Therefore, in the original manuscript, we only evaluated the transition characteristics of TADF processes, and used the results to estimate the excited-state conversion processes. We think that although this simplification ignored PH processes, it can reflect the radiative and nonradiative characteristics of the clusters in some degrees. Nonetheless, as reviewer pointed out, the evaluation of PH properties is important for comprehensively understanding the structure-properties relationship of such kind of dual-emissive cluster emitters.

Accordingly, we involved in the quantum efficiencies and lifetimes of PH components to simultaneously evaluate TADF and PH properties. These two processes were separated and respectively evaluated. This simplification can avert the interferences from complicated and mutually interactive transitions, and give information useful and rational enough for determining structure-property relationships, especially the relative variations. Actually, regarding to simultaneously considering TADF and PH processes in excited-state dynamics, to the best of our knowledge, nearly all the reports adopted the same simplification method, namely separately estimating rate constants and efficiencies of TADF and PH processes (*Chem. Sci.* **2024**, *15*, 8974-981). With respect to transition processes, TADF proceeds through RISC-mediated triplet-to-singlet upconversion, whereas PH is direct radiative decay from a triplet excited state to the ground state, corresponding to an independent triplet-radiative contribution, rather than parts of TADF processes (*Adv. Sci.* **2025**, *12*, e202503175).

Consequently, we can further clarify the differences in PH radiation between these clusters by involving in $k_r^T = \eta_{PH}/\tau_{PH}$. Through roughly estimating the contributions of PF, DF and PH to total photons in time decays (*Angew. Chem. Int. Ed.* **2014**, *53*, 6402-6406), the rate constants and quantum efficiencies of the key radiative and non-radiative transisions can be estimated. The data were updated in Supplementary Table 3 and Figure 2d. It can be noted that despite the different values, the tendencies are unchanged. Compared to $[DPPQ]_2Cu_4I_4$, the rate constants of singlet (k_r^S) and triplet (k_r^T) radiations, ISC (k_{ISC}) and RISC (k_{RISC}) of $[CzDPPQ]_2Cu_4I_4$ and $[PXZDPPQ]_2Cu_4I_4$ doped films increase by more than about 40, 8, 6 and 2 folds. Simultaneously, singlet (k_nr^S) and triplet (k_nr^T) nonradiative rate constants of $[DPPQ]_2Cu_4I_4$ doped films are remarkably larger than their k_r^S and k_r^T values, indicating

non-radiative predominance. In opposite, the $k_{\mathrm{r}}^{\mathrm{S}}$-to-$k_{\mathrm{nr}}^{\mathrm{S}}$ and $k_{\mathrm{r}}^{\mathrm{T}}$-to-$k_{\mathrm{nr}}^{\mathrm{T}}$ ratios of $[CzDPPQ]_2Cu_4I_4$ and $[PXZDPPQ]_2Cu_4I_4$ doped films are markedly increased by at least one order of magnitude. Furthermore, $k_{\mathrm{r}}^{\mathrm{S}}$, $k_{\mathrm{r}}^{\mathrm{T}}$ and $k_{\mathrm{r}}^{\mathrm{S}}$ / $k_{\mathrm{nr}}^{\mathrm{S}}$ ratio and of CBP:15% $[PXZDPPQ]_2Cu_4I_4$ are markedly larger than those of CBP:30% $[CzDPPQ]_2Cu_4I_4$, accompanied by markedly smaller $k_{\mathrm{nr}}^{\mathrm{T}}$, indicating the superiority of the former in singlet and triplet radiation enhancement and quenching suppression. Furthermore, the ISC and RISC rate constants and quantum efficienceis of CBP:15% $[PXZDPPQ]_2Cu_4I_4$ are also the largest among its congeners. These results demonstrate the merits of $[PXZDPPQ]_2Cu_4I_4$ in efficient and balanced dual-emission characteristics.

Regarding to the extraction of PF, DF and PH spectra from time resolved emission spetra from the time ranges of <1 μs, 1-100 μs and >150 μs, we should apologize for our unclear expressions inducing this misunderstanding. Actually, despite their different lifetimes, it is difficult to completely separate PF, DF and PH components. Nonetheless, according to transition characteristics, PF components should be predominant in nanosecond range, namely <1 μs; while, the interference of PF in DF and PH spectra can be negligible in microsecond range (*Chem. Soc. Rev.* **2021**, *50*, 7587-7680; J. *Mater. Chem. C* **2021**, *9*, 127-135; *ACS Appl. Mater. Interfaces* **2018**, *10*, 40001-40007). Furthermore, PH lifetimes should be longer than DF lifetimes. Therefore, the emission components in the longer time range would mainly reflect the PH profile. Then, it is rational that the emission components in the intermediate time range would be mainly contributed by DF. Furthermore, it can be noted that the lifetimes of PF, DF and PH components for all these three clusters are comparable. So, we chose these time ranges to extract PF, DF and PH spectra. We further extracted emission spectra of CBP:15% $[PXZDPPQ]_2Cu_4I_4$ in different time ranges (Supplementary Fig. 23), which show that the spectra in the time ranges of <1 μs, 1-100 μs and >150 μs are just red shifted gradually, corrresponding to PF, DF and PH predominant emissions.

The discussions and explanations were added in the relative sections. The equations for transition parameter estimation were added. Supplementary Table 3 and Figure 2d were updated. Thanks a lot!

Revision:

Descriptions and discussions on excited-state dynamics were updated:

"Thermally activated delayed fluorescence (TADF) and PH ratios were used to determine the fractions of TADF (η_{TADF}) and PH (η_{PH}) quantum efficiencies in total η_{PL}. The integrals of time decay curves give the total photon numbers contributed by TADF and PH. After subtracting PH contributions, PF and DF proportions in the rest TADF component can be further estimated by the ratios of photon numbers for PF and DF, according to their different lifetime ranges.[4] Then, PF (η_{PF}) and DF (η_{DF}) quantum efficiencies can be roughly estimated."

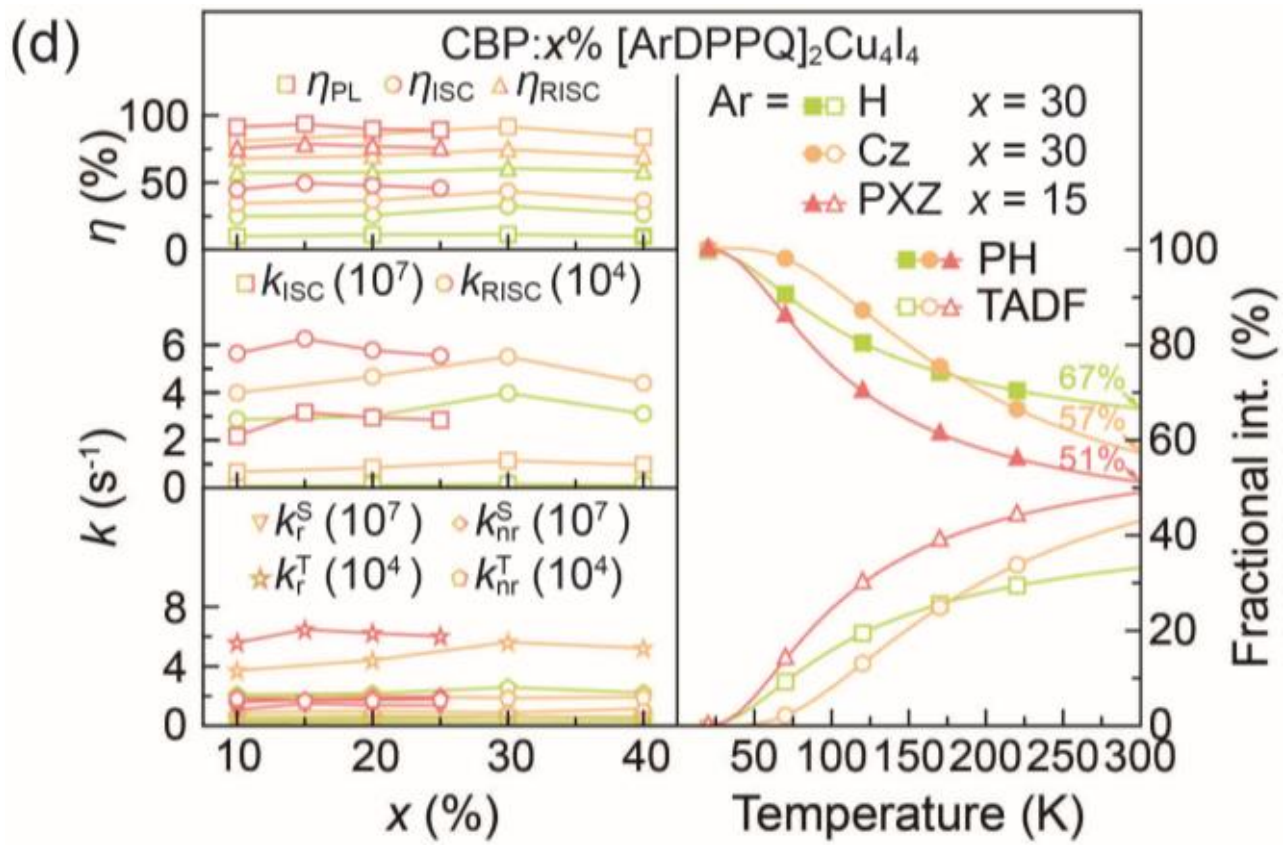

Fig. 2. (d) Dependences of transition rate constants (k) and quantum efficiencies (η) on doping concentrations for CBP:x% $[ArDPPQ]_2Cu_4I_4$ films in the ranges of x = 10-40, and temperature dependences of fractional intensities for phosphorescence (PH) and thermally activated delayed fluorescence (TADF) components of the films with optimal x% in the range of 20-300 K. PH proportions are presented and marked with arrows. Superscripts of "S" and "T" refer to singlet and triplet, respectively. Subscripts of "r" and "nr" refer to radiation and non-radiation, respectively.

"Nonetheless, compared to $[DPPQ]_2Cu_4I_4$, donor functionalization comprehensively improves the rate constants of singlet (k_r^S) and triplet (k_r^T) radiations, ISC (k_{ISC}) and RISC (k_{RISC}) by more than about 40, 8, 6 and 2 folds for $[CzDPPQ]_2Cu_4I_4$ and $[PXZDPPQ]_2Cu_4I_4$ doped films. Simultaneously, in opposite to nonradiative predominance for $[DPPQ]_2Cu_4I_4$, the ratios of k_r^S to singlet nonradiative rate constant (k_{nr}^S) and k_r^T to triplet nonradiative rate constant (k_{nr}^T) are markedly increased by at least one order of magnitude after donor modificaton (**Figure 2d** and **Supplementary Table 3**). Compared with CBP:30%

$[CzDPPQ]_2Cu_4I_4$, the markedly larger k_r^S, k_r^T and k_r^S/k_{nr}^S ratio and smaller k_{nr}^T of CBP:15% $[PXZDPPQ]_2Cu_4I_4$ manifest the combined radiation enhancement and quenching suppression. Furthermore, the rate constants and quantum efficienceis of both ISC and RISC transitions for CBP:15% $[PXZDPPQ]_2Cu_4I_4$ are simultaneously the largest among its congeners. Obviously, $[PXZDPPQ]_2Cu_4I_4$ endows its films with more efficient and balanced dual-emission characteristics."

"Spectra of prompt fluorescence (PF), delayed fluorescence (DF) and phosphorescence (PH) were sliced from time-resolved emission spectra (TRES) in the time ranges of <1 μs, 1-100 μs and >150 μs, respectively, by considering the predominance of PF, DF and PH components in the corresponding lifetime ranges."

"$k_r^T = \frac{\eta_{PH}}{\tau_{PH}}$ (Eq. S4)"

"Singlet-triplet splitting energies (ΔE_{ST}) of all the clusters are within 0.1 eV, which are less enough for RISC and subsequent TADF (**Supplementary Fig. 23** and **Table 2**)."

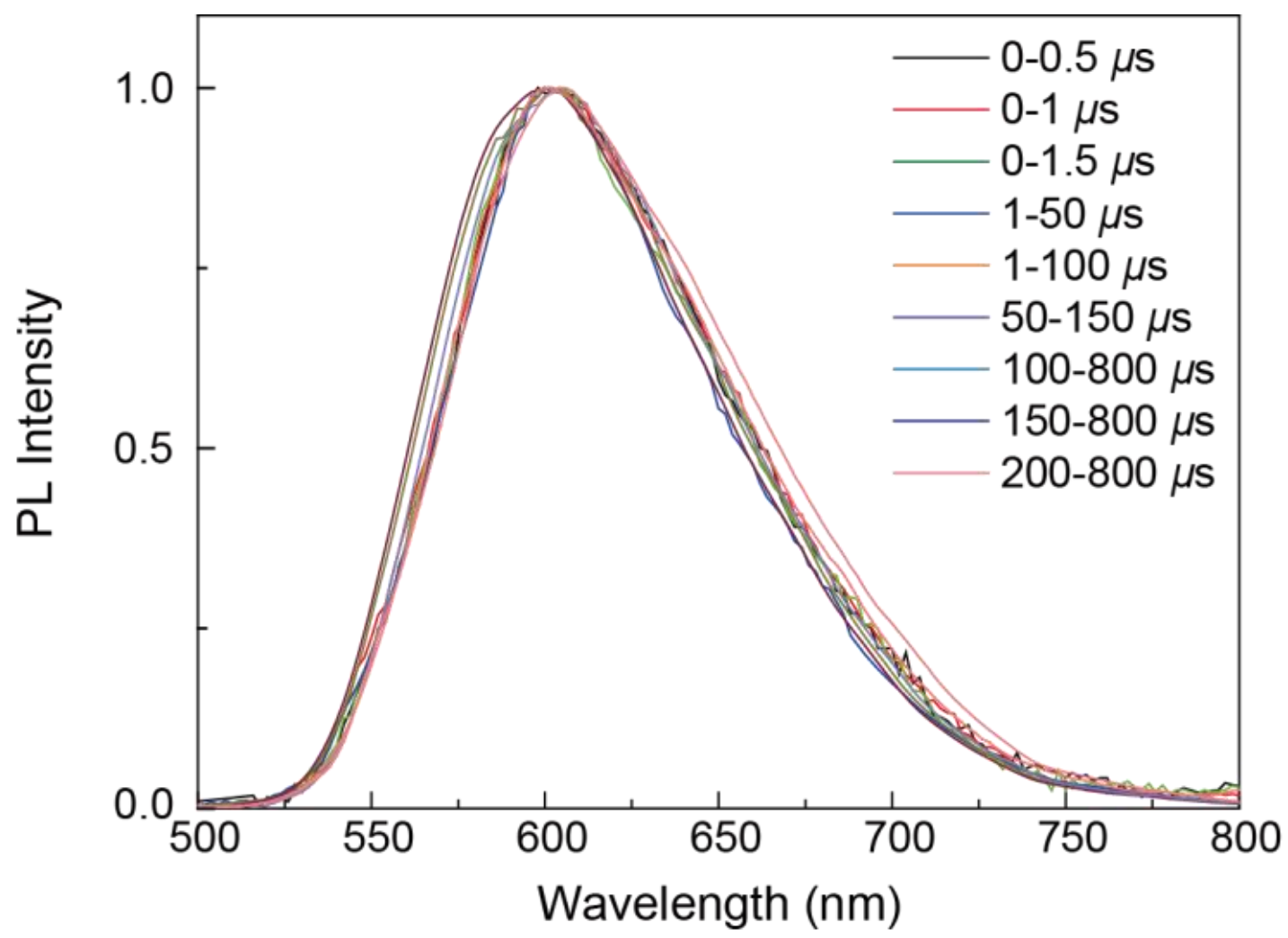

Supplementary Fig. 23 | PL spectra of CBP:15% $[PXZDPPQ]_2Cu_4I_4$ film at room temperature measured in the time ranges of 0-0.5, 0-1.0, 0-1.5, 1-50, 1-100, 50-150, 100-800, 150-800 and 200-800 μs, respectively.

"4. Lee SY, Yasuda T, Yang YS, Zhang Q, Adachi C. Luminous Butterflies: Efficient Exciton Harvesting by Benzophenone Derivatives for Full-Color Delayed Fluorescence OLEDs. *Angew Chem Int Ed* **53**, 6402-6406 (2014)."

Supplementary Table 3 | Key transition parameters of CBP:*x*% cluster films.

Cluster	x	η_{PL}[a] (%)	λ_{Em} (nm)	τ_{PF}[b] (ns)	τ_{DF}[c] (μs)	τ_{PH}[d] (μs)	η_{PF}[e] (%)	η_{DF}[f] (%)	η_{PH}[g] (%)	k_{PF}[h] (10^7 s^{-1})	k_{DF}[i] (10^4 s^{-1})	k_{ISC}[j] ($10^7 s^{-1}$)	k_{RISC}[k] (10^4 s^{-1})	k_r^S[l] (10^7 s^{-1})	k_r^T[m] (10^4 s^{-1})	k_{nr}^S[n] ($10^7 s^{-1}$)	k_{nr}^T[o] (10^4 s^{-1})	η_{ISC}[p] (%)	η_{RISC}[q] (%)
$[DPPQ]_2Cu_4I_4$	10	10.12	553	6.63	0.29	13.29	1.89	0.63	7.60	0.29	2.16	0.07	2.87	0.01	0.57	0.21	2.10	24.86	57.72
	20	11.21	555	5.65	0.33	12.89	2.14	0.74	8.34	0.38	2.23	0.10	3.00	0.01	0.65	0.27	2.17	25.64	58.06
	30	11.45	556	5.63	0.45	12.41	2.51	1.21	7.73	0.45	2.69	0.15	3.98	0.01	0.62	0.29	2.59	32.51	60.61
	40	9.98	558	4.56	0.31	12.47	1.95	0.71	7.32	0.43	2.28	0.11	3.11	0.01	0.59	0.31	2.22	26.61	58.33
$[CzDPPQ]_2Cu_4I_4$	10	80.7	597	9.43	3.69	14.19	18.25	9.64	52.80	1.94	2.61	0.67	3.99	0.35	3.72	0.91	1.88	34.57	67.95
	20	85.95	600	8.71	3.92	12.32	19.97	11.59	54.39	2.29	2.96	0.84	4.67	0.46	4.41	0.99	2.02	36.72	69.78
	30	91.61	603	8.60	5.57	9.21	22.52	17.33	51.76	2.62	3.11	1.14	5.51	0.59	5.62	0.89	1.87	43.50	74.64
	40	83.81	606	7.43	4.02	10.19	19.45	11.22	53.14	2.62	2.79	0.96	4.40	0.51	5.22	1.15	1.94	36.59	69.46
$[PXZDPPQ]_2Cu_4I_4$	10	91.37	601	4.63	5.86	9.03	22.59	18.29	50.49	4.88	3.12	2.18	5.65	1.10	5.59	1.59	1.85	44.74	75.37
	15	93.57	602	3.65	7.25	7.29	23.39	22.93	47.25	6.41	3.16	3.17	6.26	1.50	6.48	1.74	1.70	49.50	78.67
	20	89.76	614	3.63	6.78	7.53	22.40	20.48	46.88	6.17	3.02	2.95	5.78	1.38	6.23	1.84	1.73	47.77	77.02
	25	89.16	621	3.56	6.23	8.02	22.14	18.74	48.29	6.22	3.01	2.85	5.55	1.38	6.02	1.99	1.78	45.84	75.74

[a] PL quantum yield (PLQY) measured with integrating sphere; lifetimes of [b] prompt fluorescence (PF), [c] delayed fluorescence (DF) and [d] phosphorescence (PH); efficiencies of [e] PF, [f] DF and [g] PH; rate constants of [h] PF, [i] DF, [j] intersystem crossing (ISC), [k] reverse ISC (RISC), [l] singlet radiation, [m] triplet radiation, [n] singlet and [o] triplet non-radiative transitions; efficiencies of [p] ISC and [q] RISC.

2. The proposed cascade- and cross-transition mechanisms are interesting and reasonably supported by the TD-DFT/NTO analysis. As I understand it, the essential difference between CZ and PXZ appears to be the spatial localization and energetic ordering of the LCT and MLCT featured excited states, and this spatial and energetic arrangement may indeed help rationalize the different PL/EL behavior and TPQ tendency. The authors should clarify whether the improved performance is primarily attributed to this spatial/energetic arrangement or to a specific kinetic pathway. If the latter is emphasized, additional evidence for the merit of cross-transitions would be needed, since both the proposed cascade and cross pathways involve multiple IC, ISC and RISC processes, and the observed lifetimes appear to be comparable.

Response: Thanks a lot for this constructive comment! Exactly as reviewer pointed out, the spatial distribution and energetic ordering of the LCT and MLCT states directly influence the PL and EL properties of the clusters, which should be predominant in radiation facilitation and quenching suppression. Simutaneously, the cascade- and cross-transitions processes match with the different spatial and energetic distributions of the excited states for $[ArDPPQ]_2Cu_4I_4$, thereby facilitating excited-state evolution and radiative transitions. In this sense, as reviewer mentioned, excited-state distributions of the clusters mainly determine their PL and EL performances.

According to the NTO analysis, the lowest excited states of $[PXZDPPQ]_2Cu_4I_4$ are dominated by ligand-centered charge-transfer character, making its LCT states be the lowest excited states and the main radiative channels; whereas, its MLCT states contribute to the high-lying S_3 and T_3 states. It can be noted that the wave functions of the S_1 and T_1 states for $[PXZDPPQ]_2Cu_4I_4$ are similar, which is against the El-Sayed's rule, inducing spin-orbital coupling (SOC) vanished (*Nat. Commun.* **2021**, *12*, 4532; *Adv. Sci.* **2016**, *3*, 1600080). In this case, its LCT-featured S_1 and T_1 states can establish effective ISC and RISC channels with MLCT-involved high-lying excited states through cross-transitions, owing to their different wave functions and enhanced SOC. Obviously, such kind of cross-transitions accelerates the conversion from high-lying but less radiative MLCT to low-lying and highly radiative LCT states. In contrast, the S_1 and T_1 states of $[CzDPPQ]_2Cu_4I_4$ reveal MLCT-predominant character; while, its LCT-contributed high-lying T_9 state establishes a cascade-transition

pathway to MLCT-featured S_1 and T_1 states, when carrier capture and recombination firstly occur on peripheral CzDPPQ ligands.

Therefore, as reviewer pointed out, compared to its congeners, the optimized spatial and energetic distributions of excited states for $[PXZDPPQ]_2Cu_4I_4$ by introducing strong electron-donating PXZ groups not only improve its single-molecule radiation, but also influence intermolecular interactions on quenching effects: (i) the LCT-featured S_1 and T_1 states for enhancing the predominance and efficiency of radiative transitions; (ii) cross-transitions between high-lying MLCT states and low-lying LCT states for utilizing all excited energy and further balancing TADF and PH; (iii) the combination of carrier capture, recombination and radiation on peripheral ligands-centered LCT states to alleviate TPQ and simultaneously prevent high-lying and inner MLCT states from interaction induced quenching. It can be noted that the last three advantages actually originate from the first one, namely optimal spatial and energetic distributions of excited states in $[PXZDPPQ]_2Cu_4I_4$.

According to reviewer's suggestion, we further calculated the spin-orbital coupling (SOC) constants between the first ten singlet and triplet states of the clusters (Supplementary Fig. 17). It shows that for $[CzDPPQ]_2Cu_4I_4$, the SOC constants between nearly all of its first ten singlet and triplet states are high enough to support efficient ISC and RISC, which is beneficial to cascade type transitions. In contrast, the high SOC constants for $[PXZDPPQ]_2Cu_4I_4$ are respectively contributed by the first three and last three singlet and triplet excited states, indicating more concentrated ISC and RISC channels beneficial to cross-type transitions.

The related discussions were revised to improve clarity. The SOC constants between the first ten singelt and triplet excited states of the clusters were provided in Supplementary Fig. 17. Thanks a lot!

Revision:

<u>The transition modes of the clusters were discussed more appropriately:</u>

"The cascade transition mode of $[CzDPPQ]_2Cu_4I_4$ can further involve in more high-lying singlet and triplet states, making peripheral ligands incorporated into exciton formation and conversion (**Supplementary Fig. 17**). In contrast, owing to their appropriate energy gaps of ~0.2 eV and different wave functions, the S_3/T_3 and S_1/T_1 states of $[PXZDPPQ]_2Cu_4I_4$

establish the unique cross transition mode based on mutually internal conversion, vibrational coupling and three complementary ISC/RISC circles for energy convergence and population balance to its S_1 and T_1 states. Obviously, the transition modes are determined by spatial and energetic distributions of excited states.”

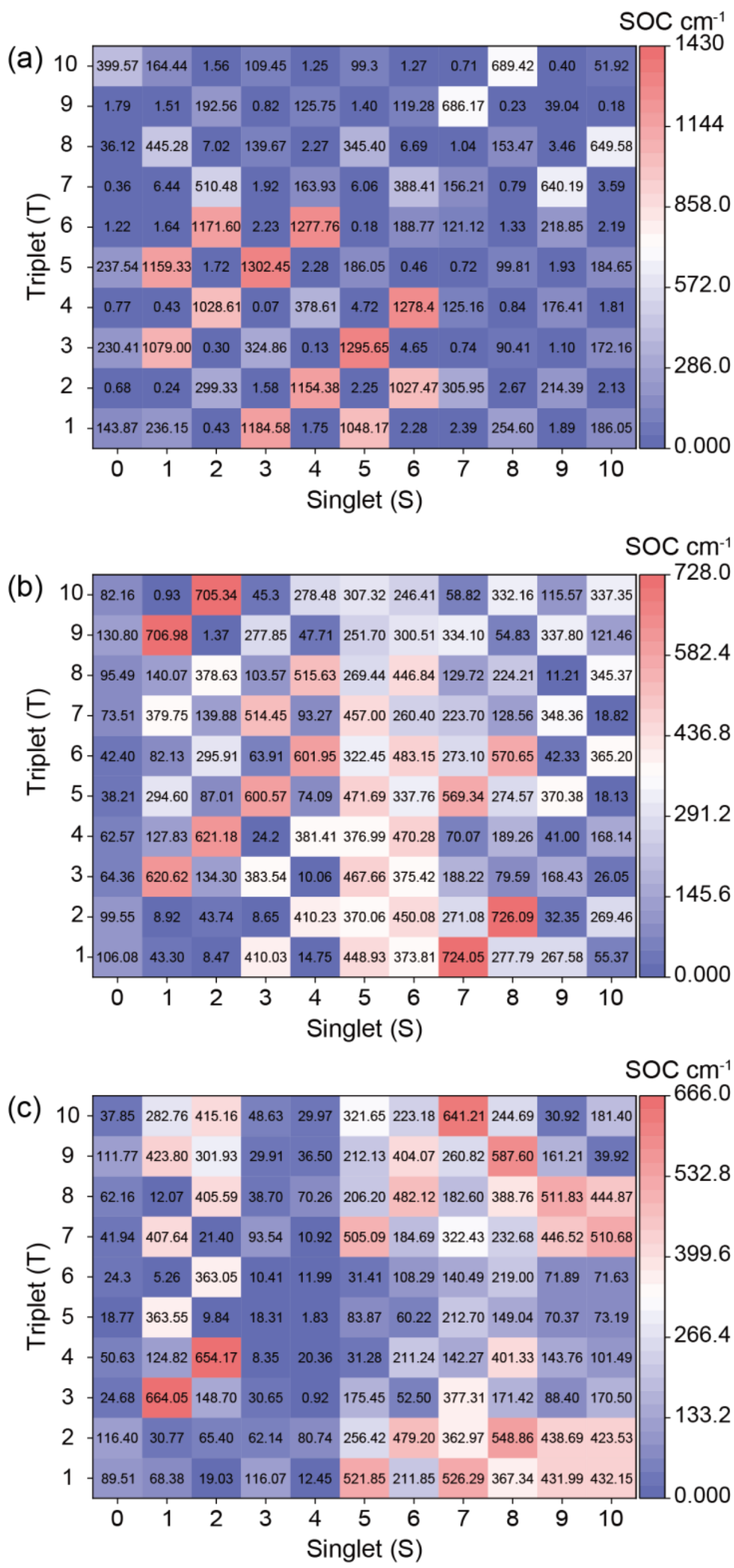

Supplementary Fig. 17 | Simulated spin-orbital coupling constants between triplet states T_m and singlet states S_n (m, n = 1-10) of (a) $[DPPQ]_2Cu_4I_4$, (b) $[CzDPPQ]_2Cu_4I_4$, and (c) $[PXZDPPQ]_2Cu_4I_4$. The color scale represents the SOC values in cm^{-1}.

Minor comments:

3. The reported maximum EQE of 43.7% for the PXZ CLED is very impressive. To better support the reliability of this value, the authors are recommended to provide reproducibility data, such as average and maximum EQE values, standard deviations or error bars. Such statistical information would make the device performance claim more convincing.

Response: Thanks a lot for this constructive comment! We should apologize for our carelessness, since we forgot to provide the data of EL performance distributions for the devices. Actually, for each device structure, 20 devices were fabricated and measured to confirm the repeatability and reliability of the EL performances. The data provided in the paper were the closest to the average values of the devices.

The description about repeating experiments was added. Supplementary Fig. 37 was provided to indicate the distribution of the maximum EQE

Revision:

Statistic analysis of the repeatability for the maximum EQE values was added:

"More Inspiringly, besides current (CE, η_{CE}) and power efficiency (PE, η_{PE}) respectively up to 87.4 cd A^{-1} and 65.3 lm W^{-1}, CBP:15% $[PXZDPPQ]_2Cu_4I_4$ endowed its device with the state-of-the-art η_{EQE} as high as 43.7% (**Figure 3d**, **Supplementary Figs 31** and **32** and **Table 4**)."

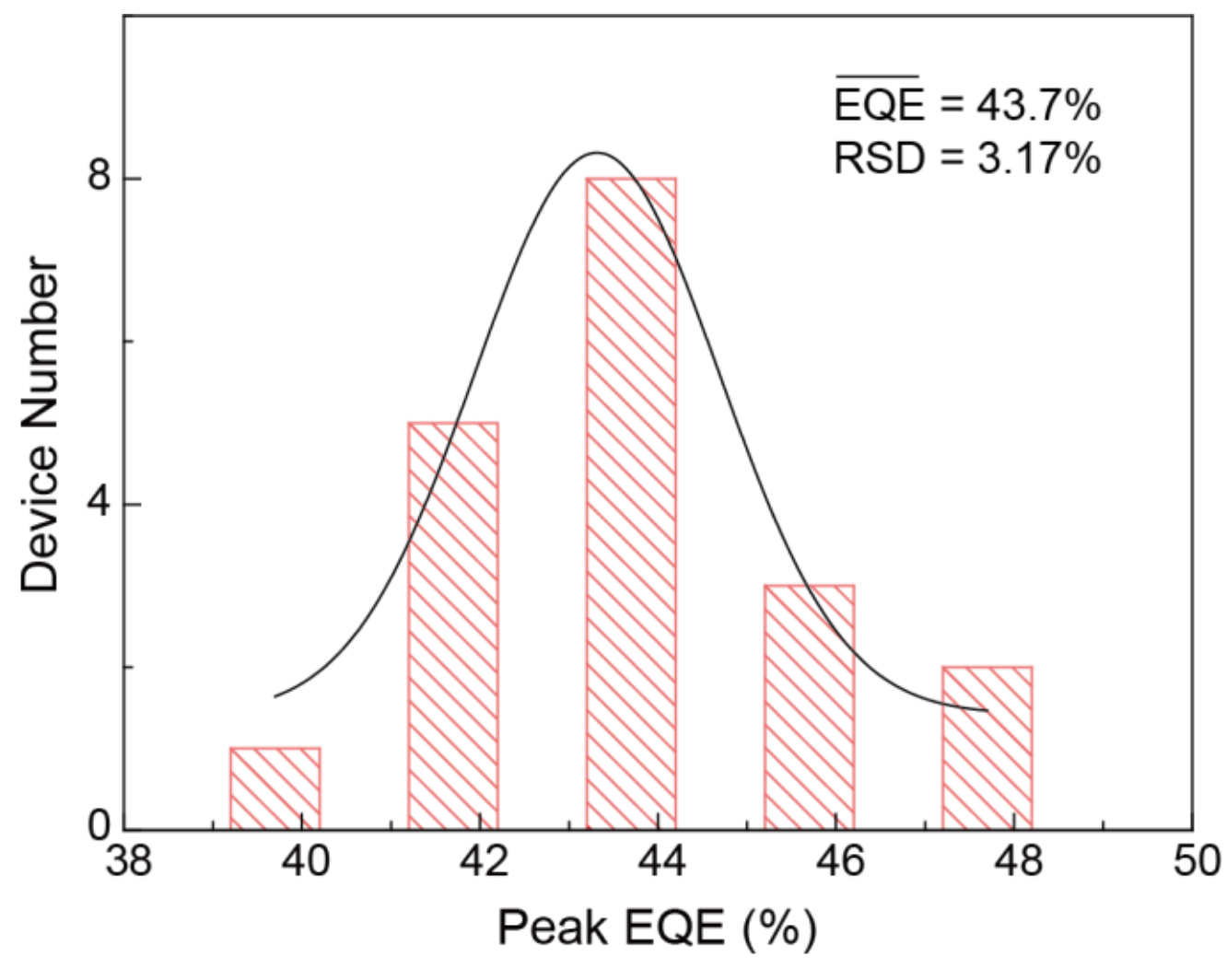

Supplementary Fig. 32 | Statistic analysis of the repeatability for the maximum EQE values of CBP:15% $[PXZDPPQ]_2Cu_4I_4$ based devices with the same structures. Device number is 20.

RSD refers relative standard deviation. The EQE variation followed normal distribution with a small RSD less than 5%.

4. Since the manuscript discusses the potential of these emitters for display applications, preliminary operational stability data of the optimized red CLEDs would be useful.

Response: Thanks a lot for this constructive comment! We agree with reviewer's opinion that the device duration is important for real application. Since the larger differences between applied electrical energy and photon energy, red light-emitting devices commonly suffered from more serious Joule heat during operation, remarkably accelerating device aging. Furthermore, in order to take use of the solution processability for the cluster emitters, the devices were fabricated with spin coating. In this case, the device structure can not be systematically optimized, especially the interfaces between the layers, since we did not use any cross-linkable carrier transporting materials. So, the carrier flux unbalance and Joule heat during operation can be hardly eliminated. Furthermore, although we used UV glue to encapsulate the devices in glovebox, the influences of moisture and oxygen can not be excluded completely. As a result, due to the technology limitation, we can not realize the device duration reaching the industrial standard for the commercial applications.

Nevertheless, we measured the device aging characteristics of $[PXZDPPQ]_2Cu_4I_4$ based devices under constant current mode (Supplementary Fig. 38). A commercial red phosphor $Ir(piq)_2acac$ was used as a control. It shows that although the aging of CBP:15% $[PXZDPPQ]_2Cu_4I_4$ based devices was faster, its lifetime at half of initial luminance was 18 minutes, which is six folds of that for $Ir(piq)_2acac$ based analogs with the same devices structure. In this case, it should be rational that under the industrial conditions, red CLEDs based on $[PXZDPPQ]_2Cu_4I_4$ have the potential as the next-generation display and lighting technologies.

The aging curves were provided in Supplementary Fig. 38. The related discussion was also added. Thanks a lot!

Revision:

Operational lifetime measurement of the OLED device was added:

"The duration of CBP:15% $[PXZDPPQ]_2Cu_4I_4$ based devices was sixfold longer than that of commercial Ir^{3+} phosphor, further verifying the potential for practical applications (**Supplementary Fig. 38**). The competence of Cu_4I_4 clusters for deep-red devices with higher color purity was also demonstrated by CBP:40% $[PXZDPPQ]_2Cu_4I_4$ with electroluminescent peak wavelength beyond 630 nm (**Figure 4f**)."

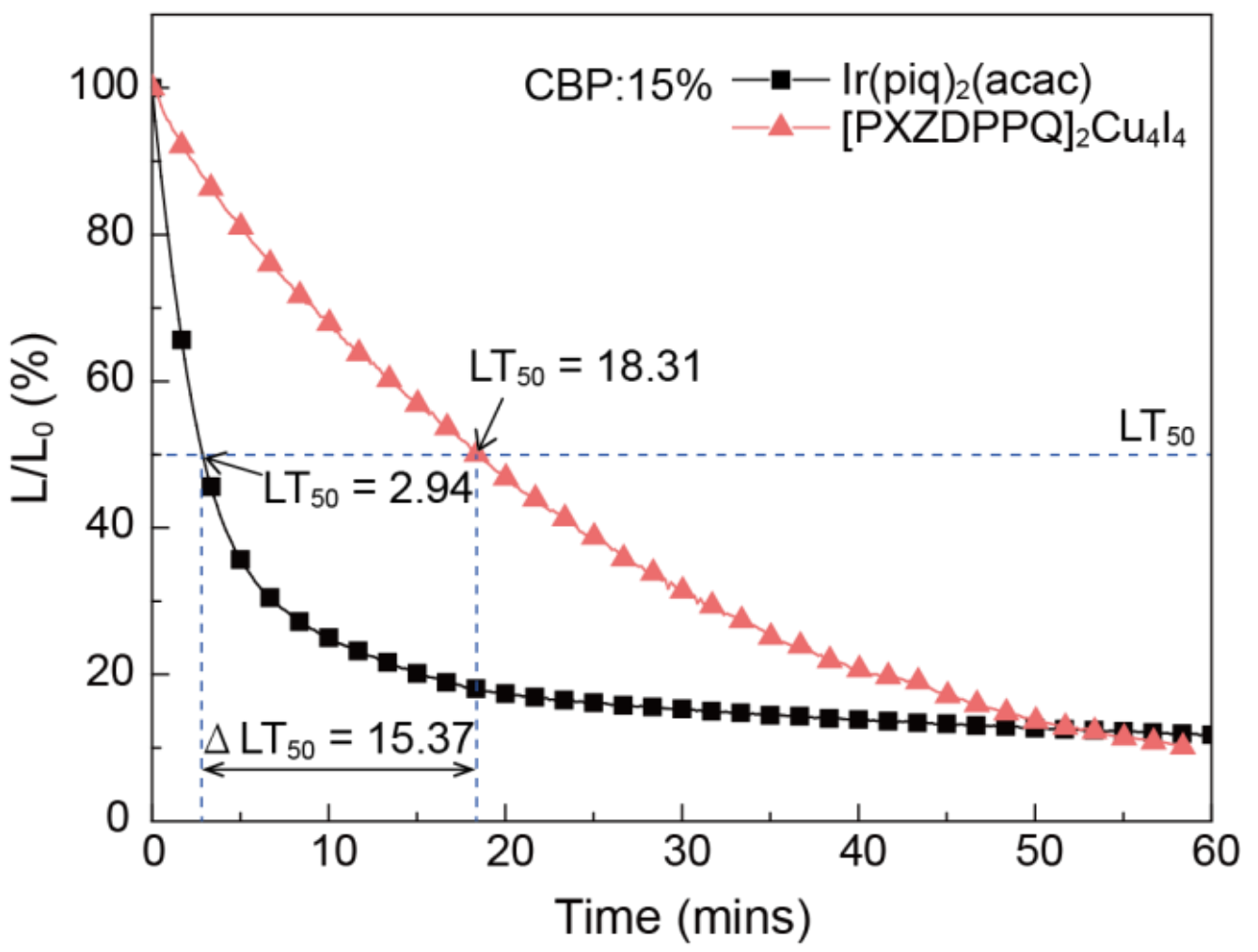

Supplementary Fig. 38 | Aging curves of the devices based on $[PXZDPPQ]_2Cu_4I_4$ under constant-current operation. LT_{50} represents the time at which the luminescence decreased to 50% of the initial value. $Ir(piq)_2acac$ (piq = 1-phenyl-isoquinoline, acac = acetylacetone) is a commercial Ir^{3+} phosphor for red-emitting OLEDs.

5. Several structure-property statements should be clarified or toned down. For example, the "remote steric effects" of CZ/PXZ (page 4, line 125) appear speculative. The phrase "doubles dihedral angles of coordination geometry" (page 5, line 146) is also unclear, please specify which dihedral angles are meant, provide the numerical values, and explain how they support enhanced rigidity.

Response: Thanks a lot for this constructive comment! We should apologize for our inappropriate expressions decreasing the clarity of the paper. As reviewer pointed out, it is not necessary to use too many rare terms, like "remote steric effect". Actually, Wong et al. reported "remote steric effect" to describe the situation of steric hindrance for groups on one side of the molecule influences intermolecular interactions on the other side (*ACS Appl. Mater. Interf.* **2017**, *9*, 7355-7361; *J. Am. Chem. Soc.* **2022**, *144*, 10186-10192). This is identical to

the situations of [CzDPPQ]$_2$Cu$_4$I$_4$ and [PXZDPPQ]$_2$Cu$_4$I$_4$ that despite large steric hindrance of DPPQ inducing linear Cu$_4$ structures in [DPPQ]$_2$Cu$_4$I$_4$, Cz and PXZ groups on the opposite sides of coordination sites provide large enough steric hindrances to counteract that of DPPQ, leading to the formation of distorted bipyramids Cu$_4$I$_4$ cores. Without introducing the term of "remote steric effect", the roles of Cz and PXZ can also be described clearly. So, this term was displaced with a sentence of clear description.

We should apologize for our unclear description. In the expression of "doubles dihedral angles of coordination geometry", the dihedral angle corresponds to the torsion angle τ(N-Cu-Cu-P) in coordination geometries of the clusters. Compared to τ(N-Cu-Cu-P) of 6.5° for [CzDPPQ]$_2$Cu$_4$I$_4$, [PXZDPPQ]$_2$Cu$_4$I$_4$ reveals a more twisted coordination geometry with doubled τ(N-Cu-Cu-P) of 12.5°, owing to its more distorted ligand configuration. As a result, in contrast to nearly coplanar structure of [CzDPPQ]$_2$Cu$_4$I$_4$, the configuration of [PXZDPPQ]$_2$Cu$_4$I$_4$ is markedly twisted. It is known that compared to the molecules with flat structures, the distorted configurations are commonly generated by large steric hindrances in the molecules, which undoubtedly significantly enhance the structural rigidity to prevent structural relaxation and suppress non-radiation of excited states (*J. Am. Chem. Soc.* **2003**, *125*, 7022; *Inorg. Chem.* **2004**, 43, 8282; *Polyhedron* **2017**, *124*, 166).

The torsion angles τ(N-Cu-Cu-P) were added in Supplementary Table 1. The related descriptions and discussions were revised to improve clarity. Thanks a lot!

Revision:

The descriptions and discussions on steric effects of the ligands and coordination geometry distortion were revised:

"The single crystal structures of [CzDPPQ]$_2$Cu$_4$I$_4$ and [PXZDPPQ]$_2$Cu$_4$I$_4$ are similar, in which four Cu^+ ions of Cu$_4$I$_4$ cores form a rhomboid, owing to the steric hindrances on the sides of Cz and PXZ groups counteracting that of quinoline (**Figure 1c** and **Supplementary Figs 10** and **11**)"

"In addition, more distorted ligand configuration doubles torsion angle τ(N-Cu-Cu-P) of [PXZDPPQ]$_2$Cu$_4$I$_4$ to 12.5° (**Figure 1d** and **Supplementary Table 1**). In contrast to nearly coplanar coordination geometry of [CzDPPQ]$_2$Cu$_4$I$_4$ with τ(N-Cu-Cu-P) of 6.5°, the markedly

twisted configuration of $[PXZDPPQ]_2Cu_4I_4$ undoubtedly enhances its structural rigidity and suppress non-radiative excited-state deactivation by structural relaxation."

Supplementary Table 1 | The selected bond lengths and angles of $[DPPQ]_2Cu_4I_4$, $[CzDPPQ]_2Cu_4I_4$ and $[PXZDPPQ]_2Cu_4I_4$.

Cluster	Angles (°)	
$[CzPPQ]_2Cu_4I_4$	P2−Cu2−Cu4−N2[a]	6.5
$[PXZDPPQ]_2Cu_4I_4$	P2−Cu2−Cu4−N2[a]	12.5

[a] Torsion angle of coordination geometry.

6. More generally, terms such as "less radiative ^{n}MLCT states and highly radiative ^{n}LCT states" (page 6, lines 168-169), should be used more cautiously or supported by quantitative evidence/references, these expressions imply general photophysical advantages that are not self-evident from the MLCT/LCT assignments alone. Similarly, the term "ideal PH/TADF ratio" (page 8, line 249) requires clarification. The manuscript appears to imply that a balanced PH/TADF ratio is beneficial, but it is not clearly explained why a nearly 1:1 PH/TADF contribution should be advantageous, especially compared with a TADF-dominant system.

Response: Thanks a lot for this constructive comment! We should apologize for such less rigorous expressions. As reviewer pointed out, the terms of "less radiative ^{n}MLCT states" and "highly radiative ^{n}LCT states" are not universal rules, but only applicable for $[PXZDPPQ]_2Cu_4I_4$. Actually, the radiative capability can be evaluated with oscillator strength. Figure 1d shows that the oscillator strength of the LCT-featured S_1 state for $[PXZDPPQ]_2Cu_4I_4$ is more than one order of magnitude larger than that of its MLCT-featured S_3 state. So, we think this expression would be rational in the case of $[PXZDPPQ]_2Cu_4I_4$. MLCT featured may sound too general if they are only based on the MLCT/LCT assignments. In the revision, we have limited these statements within the cases of $[PXZDPPQ]_2Cu_4I_4$.

We should apologize for our insufficient explanation on the advantage of dual emission, leading to reviewer's misunderstanding. The first three generations of molecular electroluminescence mechanisms are fluorescence (FL), phosphorescence (PH) and thermally

activated delayed fluorescence (TADF), all of which are based on single radiation channels through radiations from either the S_1 states for FL and TADF or the T_1 states for PH. Obviously, in these mechanisms, electro-generated excitons should converge into the single radiation channels, therefore worsening exciton accumulation and concentration quenching, which can be solved by using both the S_1 and T_1 states for radiation, namely dual emissions containing TADF and PH.

Regarding to dual emission, on account of initial singlet/triplet ratio as 1/3 for electroluminescence, there are two cases can effectively alleviate electro-generated exciton utilization, which are (i) 50% triplet excitons converted to singlet excitons, rendering singlet/triplet ratio of 62/38 (*Adv. Mater.* **2016**, *28*, 5975-5979); (ii) 25% triplet excitons converted to singlet excitons, resulting in singlet/triplet ration of 50/50 (*Angew. Chem. Int. Ed.* **2025**, *64*, e202508667). Obviously, the first situation can more effectively alleviate triplet quenching effects; while, the second situation can be simultaneously alleviate both singlet and triplet quenching effects. In this work, owing to the cross transitions between its singlet and triplet states and multiple ISC-RISC circles, $[PXZDPPQ]_2Cu$ realizes the TADF/PH ratio of 49/51, corresponding to balanced dual emission characteristics with the "ideal" singlet/triplet ratio.

The related descriptions and discussions were revised, accompanied by reference citation. Thanks a lot!

Revision:

Descriptions and discussions on excited-state characteristics and dual emission were revised:

"Consequently, in the case of $[PXZDPPQ]_2Cu_4I_4$, its high-lying but less radiative ^{n}MLCT states are embedded and therefore protected by its lowest and highly radiative ^{n}LCT states, which are opposite to the situation of $[CzDPPQ]_2Cu_4I_4$."

"Actually, moderate SOC endows copper iodide emitters30-32 with both thermally activated delayed fluorescence (TADF) and phosphorescence (PH) from their first singlet (S_1) and triplet (T_1) states.33-36 This establishes the basis for constructing dual emissive materials with suitable TADF/PH ratios.37-42 Dual emission with doubled radiation channels can

undoubtedly alleviate exciton accumulation-induced quenching, corresponding to an “ideal” TADF/PH ratio of 50/50.”
